# Long-Term Physical Activity Patterns and Risk of Future Frailty: A Systematic Review and Age-Stratified Meta-Analysis

**DOI:** 10.3390/geriatrics11040104

**Published:** 2026-08-11

**Authors:** Danai Sgouropoulou, Maria-Iosifina Kasdagli, Demosthenes B. Panagiotakos, Petros P. Sfikakis, Evrydiki Kravvariti

**Affiliations:** 1 First Department of Propaedeutic Internal Medicine, Laikon General Hospital, School of Medicine, National and Kapodistrian University of Athens, 11527 Athens, Greece; danaisg@yahoo.gr (D.S.); evkrav@med.uoa.gr (E.K.); 2 Department of Hygiene, Epidemiology and Medical Statistics, School of Medicine, National and Kapodistrian University of Athens, 11527 Athens, Greece; kasdaglimar@gmail.com; 3 Department of Nutrition–Dietetics, School of Health Sciences and Education, Harokopio University, 17676 Athens, Greece; dbpanag@hua.gr

**Keywords:** physical activity, frailty prevention, healthy aging, longevity

## Abstract

**Background/Objectives**: Physical activity (PA) interventions have the potential to delay or reverse frailty in older people, and previous meta-analyses have shown that PA reduces frailty risk. However, introduction and maintenance of PA during midlife might be more impactful as a public health intervention to reduce frailty at the population level. This study aimed to evaluate the impact of longitudinal PA patterns on future frailty risk and to examine whether it is modified by age of onset before vs. after 60 years. **Methods**: We conducted a systematic review and meta-analysis of prospective population-based cohort studies evaluating the effect of longitudinal PA trajectories on frailty risk of community-dwelling adults below and above 60 years at baseline. Risk ratios (RRs) from studies eligible for quantitative synthesis were pooled using random-effects meta-analysis. Results: Ten studies involving 111,714 participants from Europe, Asia, and North America were included in the systematic review and six in the meta-analysis. Relative to long-term physical inactivity, persistent PA trajectory was associated with 44% reduced risk of frailty (RR = 0.56, 95% CI:0.42–0.76) with moderate certainty, while inclining trajectories were associated with a 38% reduced risk (RR = 0.62, 95% CI: 0.57–0.68) with low certainty. **Results**: were similar for populations > 60 and <60 years at baseline. **Conclusions**: Sustained or increasing PA patterns over time are associated with lower risk of future frailty. These findings warrant further implementation studies to quantify the impact of public health policies promoting physically active lifestyles in midlife on the burden of frailty in aging populations.

## 1. Introduction

Over the past few decades, population aging has become one of the most significant demographic shifts worldwide, underscoring the growing need to extend not only life expectancy but also the number of years lived in good health. As longevity becomes the norm, age-related declines in physiological reserve and functional capacity represent a major public health challenge. Frailty is a multidimensional geriatric syndrome reflecting reduced physiological reserve and heightened vulnerability to stressors, with strong links to disability, loss of independence, hospitalization, and mortality [1,2]. Importantly, age-related frailty is not considered a fixed or inevitable condition but rather a dynamic and potentially reversible state, often preceded by a transitional stage referred to as pre-frailty, during which physiological vulnerability begins to emerge yet remains amenable to intervention [3].

Within a preventive context, lifestyle factors have received increasing attention, with a plethora of evidence supporting PA as a behavior as a pillar of healthy aging: Sustained engagement in moderate-to-vigorous physical across adulthood has been associated with lower cardiovascular disease in older age [4], as well as lower all-cause mortality [5]. Furthermore, PA plays a central role in sustaining physiological reserve [6], a key component in the development and progression of frailty with advancing age [7]. Evidence from interventional studies supports the benefits of structured exercise interventions on frailty-related outcomes, with systematic reviews and meta-analyses in older adults indicating that multicomponent and resistance-training-focused interventions, often incorporating aerobic, balance and flexibility components, can improve physical function and reduce frailty incidence or delay progression [8,9,10,11]. More recently, a systematic review focusing on middle-aged populations reported that multicomponent exercise interventions and resistance training may also yield beneficial effects on frailty-related outcomes in this age group [12].

In recent years, epidemiological studies have begun to investigate whether engagement in regular exercise during midlife and late midlife is associated with subsequent frailty risk in older age or may slow frailty progression and, in some cases, reverse frailty among older adults. Rogers et al. reported that moderate and vigorous PA was associated with slower frailty progression over a 10-year period in adults aged 50+, based on data from the English Longitudinal Study of Ageing [13]. Another longitudinal study reported that engagement in healthy lifestyle behaviors, including exercise, was associated with a lower risk of incident frailty at the 5-year follow-up. Among participants who were frail at baseline, these behaviors were also associated with transitions to a non-frail status [14]. Similar findings were reported by Zhang et al. in a multicenter European cohort of adults aged 70 years and older, where maintaining or increasing the frequency of moderate PA over 12 months was associated with changes in physical, psychological, and social frailty assessed over the same period [15]. Congruent with these findings, previous systematic reviews and meta-analyses of epidemiological studies have consistently reported an inverse association between physical activity and frailty risk [16,17]. However, these reviews primarily synthesized evidence according to PA volume rather than longitudinal physical activity patterns across adulthood. Furthermore, to our knowledge, no previous meta-analysis has examined whether the age window during which long-term PA patterns were adopted modifies their association with subsequent frailty outcomes. It therefore remains unclear whether initiating and sustaining PA earlier in adulthood provides greater long-term protection against frailty than activity adopted later in life. To address this knowledge gap, we conducted a systematic review to synthesize the available literature on the impact of longitudinal PA patterns in midlife, early old age and middle old age on the risk of frailty in later life.

## 2. Methods

### 2.1. Protocol and Registration

This systematic review was conducted and reported in accordance with the Preferred Reporting Items for Systematic Reviews and Meta-Analyses (PRISMA) 2020 guidelines [18] (Supplementary Material File S1/Supplementary Table S1) and was registered in PROSPERO (registration number: CRD420251168483) prior to application of the search strategy.

### 2.2. Search Strategy and Selection of Sources of Evidence

A comprehensive literature search was conducted in PubMed, Embase, and Web of Science from inception to June 2025. The search strategy was developed to identify observational studies evaluating the association between longitudinal PA patterns and frailty. Controlled vocabulary (MESH terms, where applicable) was combined with free-text terms related to three predefined concepts: physical activity, long-term exposure, and frailty. The keywords used for PubMed were as follows (equivalent terms used for Embase and Web of Science appear in the Supplementary Material File S1/Supplementary Table S2):

(“Exercise” [MeSH] OR “Motor Activity” [MeSH] OR “physical activity” [TIAB] OR “Exercise” [TIAB] OR “aerobic exercise” [TIAB] OR “resistance exercise” [TIAB] OR “strength exercise” [TIAB] OR “isometric exercises” [TIAB] OR “daily activity” [TIAB] OR “daily physical activity” [TIAB] OR “leisure activity” [TIAB] OR “leisure-time physical activity” [TIAB] OR “activity level*” [TIAB]) AND (“Trajectories” [TIAB] OR “Patterns” [TIAB] OR “Long-Term” [TIAB] OR “Longitudinal” [TIAB] OR “incidence” [TIAB] OR “growth curve*” [TIAB] OR “follow-up” [TIAB] OR “prospective” [TIAB] OR “cohort” [TIAB]) AND (“Frailty” [MeSH] OR “Frail Elderly” [MeSH] OR “Frailty Syndrome” [TIAB] OR “frailty risk” [TIAB] OR “frailty incidence” [TIAB]).

The search was restricted to studies published in English. All retrieved records were imported into a citation management application, and duplicates were removed prior to screening. No restrictions were applied on study setting or geographic region. The complete database-specific search strategy, including search fields and query combinations, is provided in Supplementary Material File S1/Supplementary Table S3.

**Figure 1:**
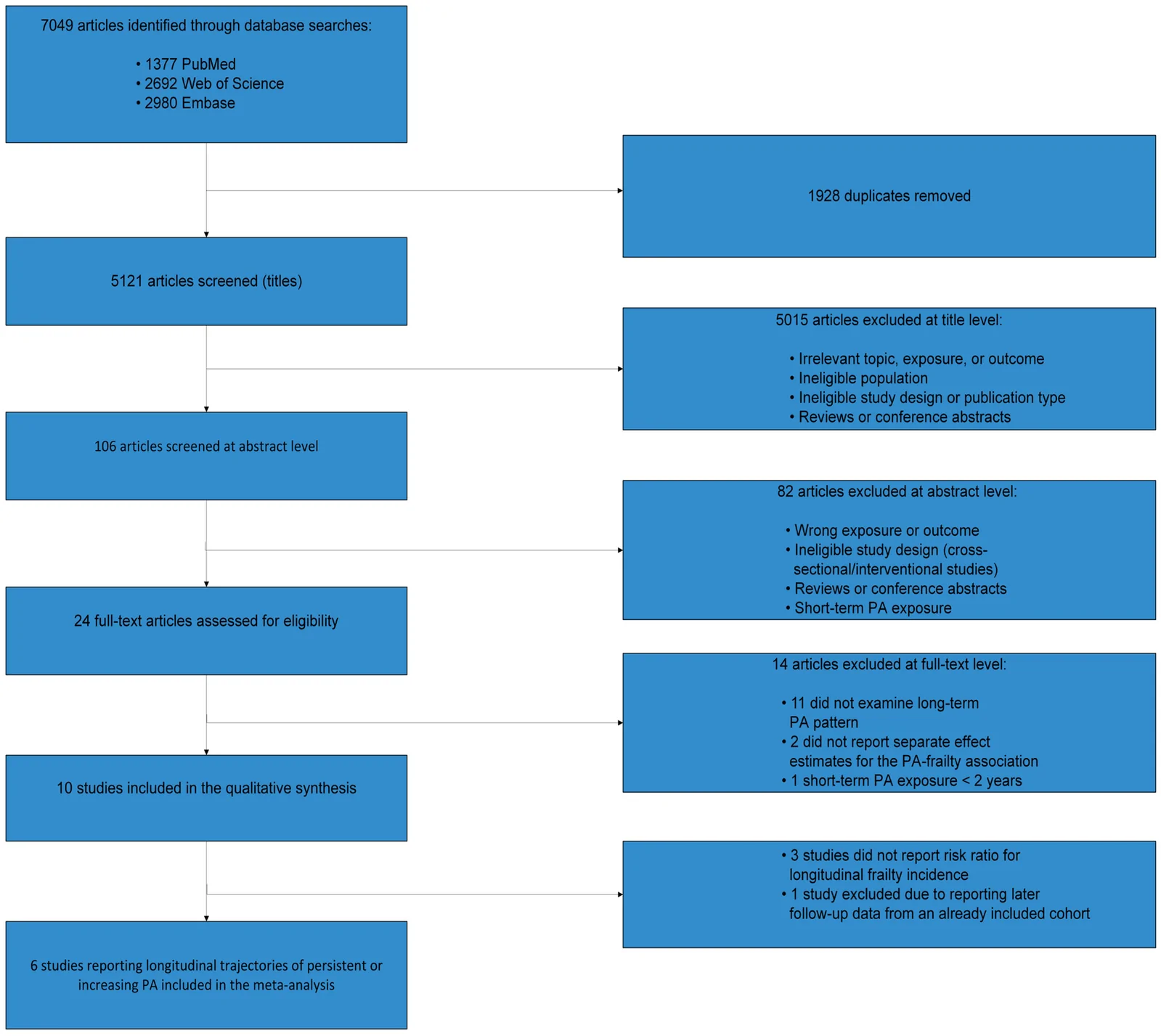
Flow chart of the study selection process.

Titles, abstracts, and full-text articles were independently screened for relevance by two researchers (D.S. and E.K.) according to the predefined eligibility criteria. Any disagreements were resolved through discussion and consensus with a third researcher (P.P.S.) if needed. During title and abstract screening, records were excluded using predefined exclusion categories (Supplementary Material File S1/Supplementary Tables S3 and S4). When multiple exclusion criteria applied, each record was assigned a single primary reason for exclusion to avoid double counting. The overall study selection process is presented in the PRISMA 2020 flow diagram (Figure 1).

### 2.3. Eligibility Criteria

We included observational population-based studies that (a) examined the association between PA and frailty, (b) assessed PA in midlife (mean age ~45–60 years) or older age (mean age > 60 years), (c) evaluated PA as a longitudinal pattern or trajectory (rather than only a single baseline measure), and (d) reported frailty either as a binary outcome or a continuous score based on a validated definition (e.g., Fried phenotype, Frail scale, Frailty Index, Kihon Checklist).

We excluded studies that focused exclusively on cross-sectional associations, lacked a clear operationalization of frailty, or were conducted in populations with specific chronic diseases (e.g., cancer, Parkinson’s disease). We also excluded studies that involved institutionalized individuals, such as nursing home residents or hospitalized patients. Furthermore, interventional studies (including randomized and non-randomized controlled trials, clinical trials, pilot intervention studies, and other experimental intervention studies) in which PA was administered as a therapeutic treatment to reverse or manage existing frailty were not eligible, as our aim was to capture naturalistic, observational PA patterns prior to or during frailty development. Studies in which PA was not assessed as an independent exposure of interest, or those where PA was only included as one component of a broader lifestyle index without separate longitudinal evaluation, were excluded. Studies were eligible only if PA was assessed longitudinally, defined as including at least one of the following: repeated measurements over time, trajectory-based analyses, or explicit characterization of long-term engagement patterns. This ensured conceptual consistency across studies and permitted the focus on longitudinal or sustained patterns of physical activity in relation to frailty development. Reviews, conference abstracts, study protocols, case reports and animal studies were also excluded.

### 2.4. Data Charting Process

A standardized data extraction form was developed and applied to all included studies. One reviewer extracted the data, which were then cross-checked independently by a second reviewer. Extracted information included study characteristics (first author, year, country, sample size), population details (mean age at baseline, follow-up duration), physical activity exposure (assessment method, number and timing of measurements, trajectory or longitudinal pattern classification), frailty definition and assessment method, and key findings.

To ensure consistency and comparability across studies, physical activity exposures were harmonized into comparable longitudinal patterns before data synthesis. Specifically, we extracted risk ratios of persistent vs. low physical activity throughout follow-up, and, where available, we extracted inclining (baseline low to higher on follow-up) physical activity vs. always-low physical activity trajectories. When stratified estimates of longitudinal PA patterns were reported for different PA levels, we selected the estimate for the highest PA level (i.e., vigorous over moderate). When multiple follow-up periods were reported, we selected the longest. To allow for comparison of the effects of different physical activity trajectories on different age groups, we categorized cohorts according to participant age range at baseline (45–60, 61–75, >75); when age range was not reported, we used the mean participant age to categorize cohorts. Sub-cohorts with overlapping age ranges not reporting mean age were excluded from the age-stratified meta-analysis. When more than one analytical model was presented, multivariable-adjusted estimates for incident frailty were extracted rather than crude estimates.

### 2.5. Risk of Bias Assessment and Certainty of Evidence Grading

The risk of bias of the included studies was assessed independently by two researchers (D.S., E.K.) using the Risk Of Bias In Non-randomized Studies of Exposures (ROBINS-E) tool [19]. ROBINS-E evaluates the risk of bias arising from confounding, participant selection, exposure classification, departures from intended exposures, missing data, outcome measurement, and selection of the reported result. Each domain, as well as the overall risk of bias, was categorized as” low risk”, “some concerns”, “high risk” or “very high risk”.

The GRADE approach was used to grade the certainty of evidence derived from the meta-analysis, starting from low level of certainty as the baseline, due to the observational design of the included studies [20].

### 2.6. Statistical Analysis

We used relative risks (RRs) as the common measure of association. Hazard ratios (HRs) were considered equivalent to RRs, and odds ratios (ORs) were also treated as equivalent to RRs because the incidence of frailty in ambulatory older adults in community settings usually is below 15%, allowing ORs to approximate RRs under the rare outcome assumption. To ensure robustness of our findings under this assumption, we also performed an exploratory subgroup analysis examining estimates from studies reporting ORs vs. studies reporting HRs. Random-effects meta-analysis using the restricted maximum likelihood (REML) method was used to estimate the pooled RRs, and we assessed heterogeneity with the Cochran’s Q test and the I^2^ statistic. To explore expected sources of heterogeneity, we performed subgroup analyses by study length of follow-up (shorter vs. longer than the mean follow-up time) and by participant age group at baseline (above vs. below 60 years of age); study subgroups spanning both age categories (i.e., 55–65 years old) not reporting mean age were not included in the age-stratified analysis. To avoid unit-of-analysis bias, in studies reporting multiple age subgroup estimates we only included one subgroup below and one above 60 years in a symmetric age distribution around age 60. Publication bias was assessed by funnel plots. All analysis was performed using the “meta” package (version 8.2-0) in RStudio (version 2024.04.2+764).

## 3. Results

The study selection procedure is shown in Figure 1. A total of 7049 articles were identified through electronic database searches. After removing 1928 duplicates, 5121 records underwent title screening. Of these, 5015 were excluded, primarily because they addressed irrelevant research questions or ineligible exposures or outcomes, included disease-specific or institutionalized populations, evaluated interventional or other ineligible study designs, or represented reviews, conference abstracts, animal studies, case reports, or other ineligible publication types. As some records fulfilled more than one exclusion criterion, each record was assigned to a single exclusion category according to the reason considered most relevant by the reviewers, ensuring that each record was counted only once. The remaining 106 records underwent abstract screening, during which 82 studies were excluded mainly because of ineligible study designs (including cross-sectional and interventional studies), wrong exposure or outcome, disease-specific populations, conference abstracts, reviews, or assessment of only short-term physical activity exposure (Supplementary Table S4). Twenty-four full-text articles were subsequently assessed for eligibility, and 14 were excluded for the following reasons: no long-term patterns of PA examined (*n* = 11) [13,14,21,22,23,24,25,26,27,28,29], PA not evaluated or reported separately for the PA–frailty association (*n* = 2) [30,31], and short-term PA exposure (<2 years) (*n* = 1) [15]. Ten studies met the inclusion criteria and were included in this review. Results were summarized and compared across studies within predefined age categories at physical activity assessment (midlife, early old age, and middle old age), with particular emphasis on consistency of associations and patterns of long-term engagement in PA.

The included studies reported on a total of 111,714 participants across 10 prospective cohort studies. The sample size of individual studies ranged from 514 to 69,642 participants and spanned a wide age range, from midlife (45–60 years) through older age (>60 years), allowing for examination of physical activity patterns across critical stages of the aging process. All studies were conducted in community-dwelling populations and originated in Europe, Asia, and North America, including cohorts from Finland [32,33] the United Kingdom [34,35], Mexico [36], China [37], Taiwan [38], Japan [39], South Korea [40], and the United States [41]. The majority of studies included both male and female participants in roughly equal proportions; one included only males [33], and one only females [41]. The median follow-up ranged from 2.35 to 26 years.

Frailty was evaluated using a modified Fried phenotype in one study [33], Frailty Index-based approaches in seven studies [32,34,35,36,37,38,40], the FRAIL scale in one study [41], and the Kihon Checklist in one study [39]. PA was evaluated using longitudinal assessments based on repeated self-report measures in seven studies [33,34,35,36,37,38,40], MET-based calculations in two studies [32,41] and structured program participation in one study [39]. The longitudinal patterns of PA were operationalized using different approaches across studies. Two studies applied formal group-based trajectory modelling [34,38], five relied on repeated longitudinal physical activity assessments to characterize exposure over time [32,33,35,39,41], and three classified participants into predefined longitudinal change or sustained engagement categories [36,37,40]. Six studies reported multivariable RRs for the effect of longitudinal PA on frailty outcome measures, adjusted for age and sex, as well as variable other confounders, including educational level, comorbidities, body mass index (BMI), smoking, alcohol, and marital status [34,36,37,38,40,41]; these were included in the meta-analysis. Key characteristics of the included studies, including population features, PA assessment, frailty measurement, frailty cases and outcome definitions, are summarized in Table 1. A detailed overview of the longitudinal physical activity exposure, including assessment time points, exposure windows, activity definitions, reference groups, and the use of formal trajectory modelling, is provided in Supplementary Table S5, to facilitate interpretation of the pooled analyses.

Studies assessing PA during midlife (baseline ages ≈ 47–60 years) consistently reported lower subsequent risk of frailty in older age with persistent vs. low PA longitudinal patterns [33,34]. In the Helsinki Businessmen Study [33], high midlife PA was associated with approximately 80% lower risk of frailty and 47% lower risk of pre-frailty over a 26-year follow-up. The study also examined long-term changes in PA from midlife to older age over a 26-year period. Men who maintained high activity had no frailty cases (0%), while those who increased activity showed a frailty prevalence of 4.2%. In contrast, decreasing activity was associated with higher frailty prevalence (16.6%), and persistent inactivity with the highest prevalence (40.7%). Evidence from the English Longitudinal Study on Aging (ELSA) cohort [34] also indicated that midlife-to-late-midlife activity patterns were linked to incident frailty assessed approximately 10 years later, providing a clear temporal separation between exposure and outcome. Maintaining moderate and high activity over 6 years was associated with 30% and 58% lower risk, respectively. Additionally, increasing activity is correlated with 40% lower risk, compared with the persistently low-active trajectory, while the declining trajectory did not show a significant relationship with the risk of developing frailty.

Studies assessing PA during early old age (baseline ages ≈ 60–75 years) reported associations between inclining or persistent PA and lower risk of incident frailty, whereas declines in activity were associated with higher frailty risk [32,35,36,38,40,41]. In Borda et al. [36], involving Mexican adults aged ≥60 years, regular physical activity—defined as ≥3 sessions per week over the preceding 2 years—was associated with a 30% reduction in the 3-year incidence of frailty. In the Nurses’ Health Study [41], among women in the United States aged ≥60 years at baseline, higher vs. lowest total PA levels—assessed repeatedly over a 20-year-period using cumulative averages—were associated with a 52% lower risk of incident frailty over up to 24 years of follow-up, with moderate activity conferring a 49% lower risk, and vigorous activity a 25% lower risk. Changes in activity over time were also examined: Over 8-year periods, large reductions (≤10 MET-hours/week) in total activity were associated with a 19% higher frailty risk, whereas large increases (≥10 MET-hours/week) were associated with 34% lower risk. Lin et al. [38], using 20-year PA trajectories from the Taiwan Longitudinal Study on Aging (TLSA), reported that consistently active adults aged ≥60 years had a 63% lower risk of frailty, and those with increasing activity had a 55% lower risk, compared with consistently inactive individuals. In contrast, declining activity trajectories were associated with increasing frailty rates over time. Although including adults aged ≥45 years from the Korean Longitudinal Study of Aging (2006–2022), the study by Jung et al. [40] was classified in the late-midlife category because the mean age of the cohort, estimated from the reported age distribution, was in the mid-60s. The study reported a dose–response relationship, with persistently active individuals showing 55% lower risk of incident frailty and those increasing activity showing 43% lower risk. Declining PA was associated with higher frailty risk, with particularly strong associations observed among participants aged 45–54 years. The same study also examined exercise characteristics and reported that longer exercise sessions (≥30 min) were more strongly associated with lower frailty risk than exercise frequency alone. Haapanen et al. [32], including both non-frail and frail participants at baseline in the Helsinki Birth Cohort Study (HBCS), examined frailty progression rather than incidence over a 17-year period. Discontinuation of regular PA was associated with faster increases in frailty, whereas consistent PA and resolution of sleep disturbances were associated with slower frailty progression. Finally, using data from the English Longitudinal Study of Ageing (ELSA), Huang et al. [35] investigated frailty transitions among adults aged ≥50 years, with a median age of 67 years, over a mean follow-up of 3.5 years. Among participants who were non-frail at baseline, increasing vigorous and moderate physical activity was associated with a 65% and 64% lower risk of frailty worsening, respectively, whereas persistently vigorous and moderate physical activity was associated with an 80% and 87% lower risk, respectively. Conversely, participants with declining vigorous and moderate physical activity had a 35% and 36% lower risk of frailty worsening, respectively, compared with those who remained inactive.

Two studies examined the association between PA and frailty among adults in middle old age (≥74 years) [37,39]. In the Japanese cohort by Hayashi et al. [39], longer-term participation in resistance exercise was associated with significantly lower frailty scores and slower frailty progression over time, compared with short-term participation. In the Chinese CLHLS cohort [37], Zhang et al. reported that persistent aerobic exercise was associated with a 26% lower risk of incident frailty over a median follow-up of 3.1 years. The protective association was even stronger in the oldest old age group (≥90 years), among whom persistent exercise was linked to a 28% lower risk. The study further evaluated frailty incidence among individuals with higher genetic predisposition to frailty and found that persistent aerobic exercise attenuated the excess risk.

The findings summarized above derive from studies with diverse designs, follow-up durations, and approaches to assessing PA and frailty, which differ in the strength of temporal and inferential evidence they provide. To aid interpretation of the evidence, the main methodological strengths and limitations across included studies are summarized in Table 2. One study [32] was judged to be at low risk of bias, seven studies [33,34,35,38,39,40,41] were judged to have some concerns, and two studies [36,37] were judged to be at high risk of bias. The most common sources of concern were residual confounding, self-reported physical activity assessment, and missing data. A detailed study-level ROBINS-E assessments is provided in Supplementary Material File S1 (Supplementary Material File S1/Supplementary Table S6).

### Pooled Estimates of PA Impact on Frailty Prevention

Of the studies included in the systematic review, 6 studies involving 99,234 individuals reported outcomes that could be synthesized in the meta-analysis: Of those, two studies reported risk ratios only for persistently active vs. low PA trajectories [36,37], and another two reported risk ratios only for inclining activity vs. low PA trajectories [34,41]; the remaining two studies reported risk ratios for both exposures [38,40], of which data from Jung et al.’s study [40] were incorporated using age-specific subgroup analyses and were included as two separate entries in the meta-analysis (one entry for the subgroup of participants 45–55 years old at baseline, and one for the subgroup of participants 65–75 years old at baseline). Care was taken to ensure that no study participants were double-counted, maintaining the statistical independence of each data point; for this reason, the study of Huang and colleagues [35], reporting on data from waves 9–10 of the ELSA cohort, was not included in the meta-analysis, in favor of including data reported by Wang et al. from earlier waves (1–9) of the same cohort. According to the ROBINS-E scale, some concerns arose for several studies, and only two were considered at high risk of bias (Table 2 and Supplementary Material File S2).

**Figure 2:**
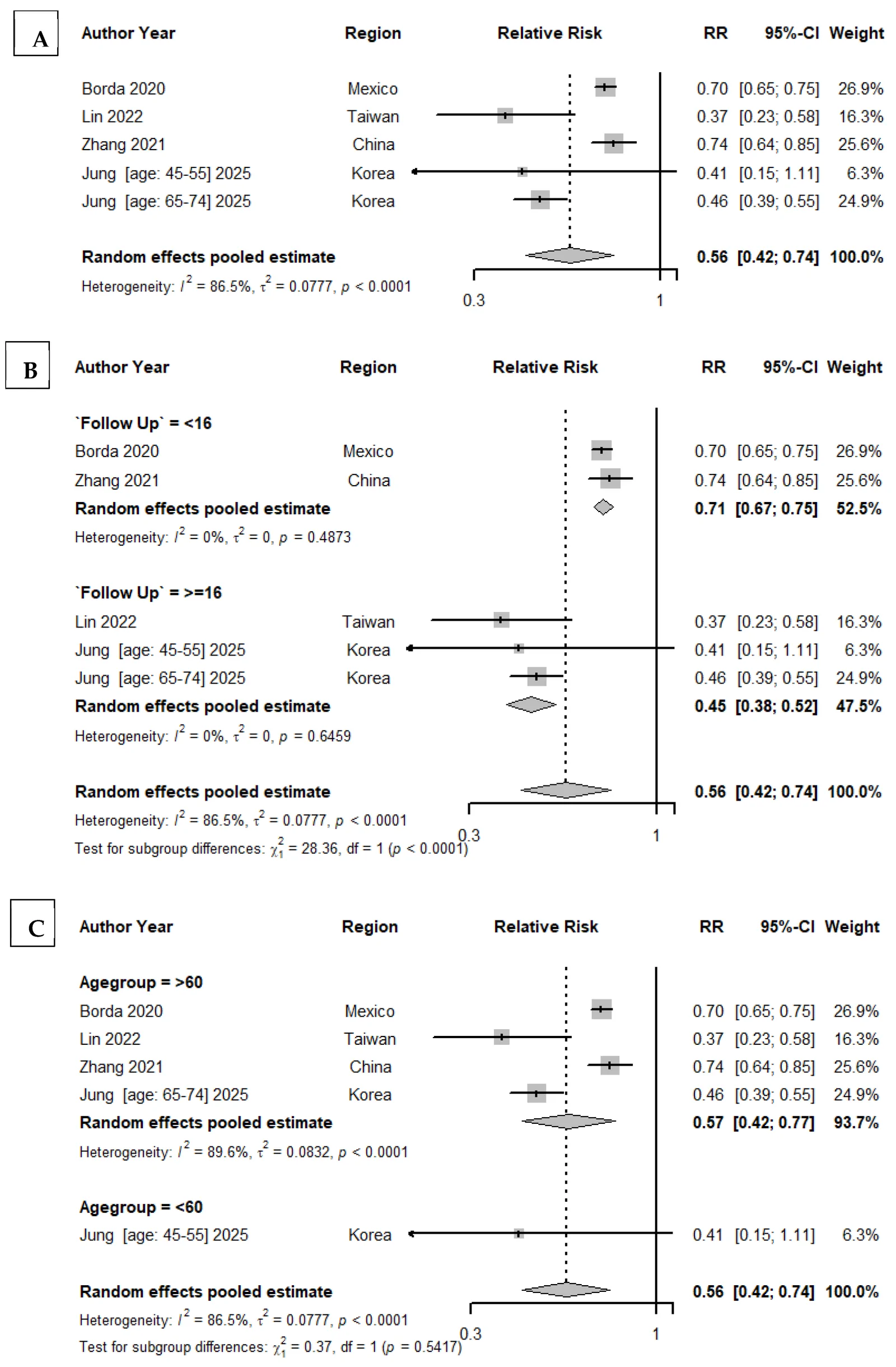
Forest plots of studies reporting risk ratios for the impact of persistently active vs. low PA trajectories: (**A**) overall, (**B**) stratified analysis by follow-up length (≤16 vs. >16 years), (**C**) stratified analysis by participant baseline mean age (>60 and <60 years) [36,37,38,40].

Aggregate results of the studies reporting on the impact of the persistently active trajectory are shown in Figure 2. The pooled estimate of all studies, including 23,813 patients, was RR = 0.56 (95% CI: 0.42–0.74) (Figure 2A), which was similar in studies of participants > 60 years, and not significantly different in the single study reporting on the impact of this exposure on future frailty risk in persons younger than 60 years (Figure 2C). Notably, in the latter study, the result was not statistically significant due to a wide confidence interval (RR = 0.41, 95% CI: 0.15–1.11). In the investigation of sources for the high heterogeneity via subgroup analyses, length of follow-up arose as a significant determinant, explaining heterogeneity between studies to a significant degree: Studies spanning < 16 years of follow-up demonstrated an aggregate 30% reduced risk of frailty development, on average, whereas studies spanning > 16 years demonstrated a 50% risk reduction (Figure 2B). Subgroup analysis according to type of estimate reported (OR vs. HR) was of limited value, as only one study reported HR for the impact of persistent PA on the risk of frailty (Supplementary Material File S1/Supplementary Figure S1).

**Figure 3:**
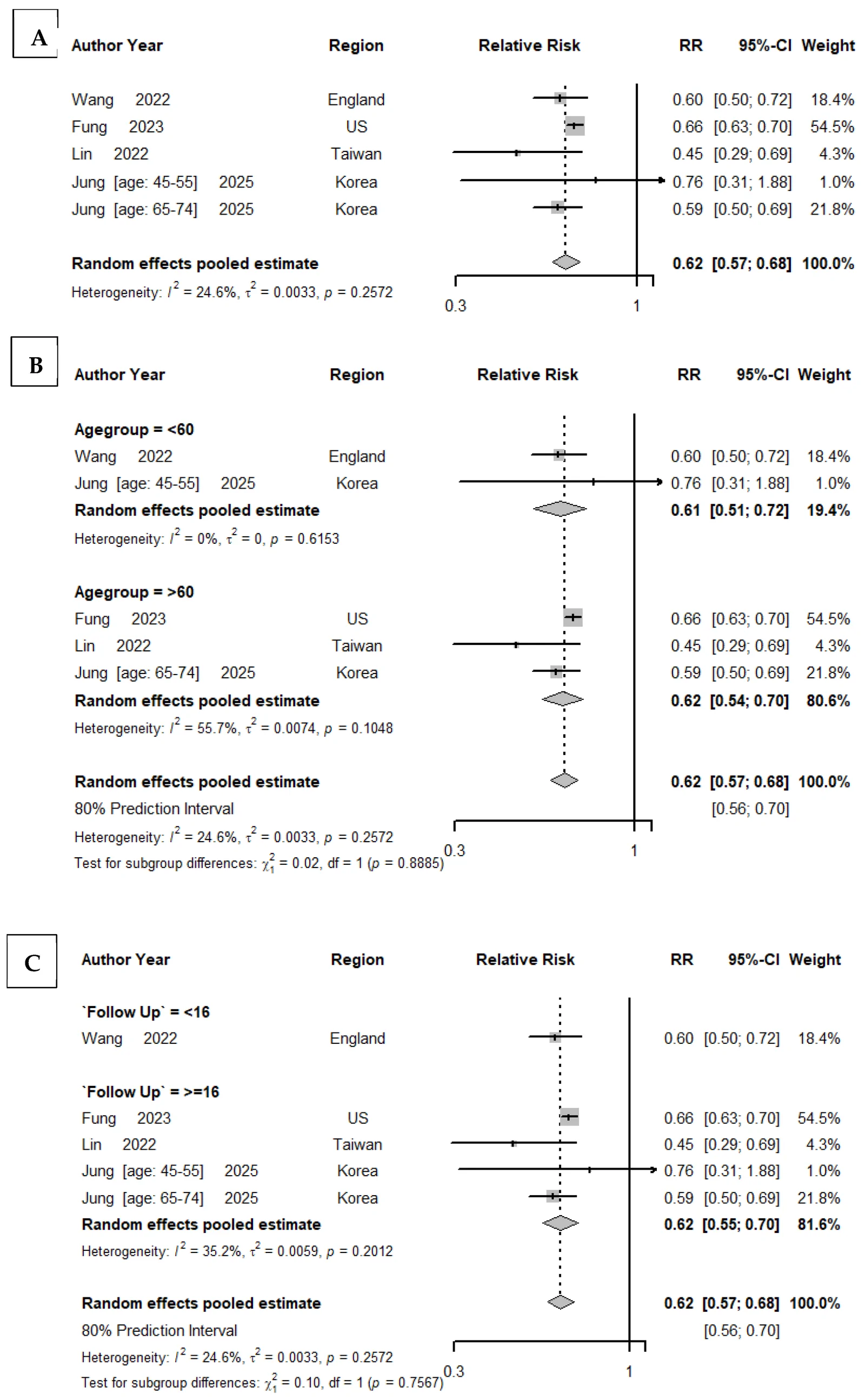
Forest plots of studies reporting risk ratios for the impact of inclining activity vs. low PA trajectories: (**A**) overall, (**B**) stratified analysis by follow-up length (≤16 vs. >16 years), (**C**) stratified analysis by participant baseline mean age (>60 and <60 years) [34,38,40,41].

Aggregate results of the studies reporting on the impact of inclining activity trajectory on frailty incidence, shown in Figure 3A, were also supportive of a statistically significant, albeit more modest, protective effect: The pooled estimate of all studies, including 88.594 patients, was 0.62 (95% CI: 0.57–0.68); again, significant heterogeneity was detected (I^2^ = 24.6%, *p* = 0.026), which was not explained by stratifying for follow-up length for this PA pattern (Figure 3B). The observed risk reduction was similar both in the three reports on populations aged older than 60 years (pooled RR = 0.62, 95% CI: 0.54–0.70) and in the two reports on populations younger than 60 (pooled RR = 0.61, 95% CI: 0.51–0.72) (Figure 3C). There was no significant difference in the pooled estimates of the two studies reporting HRs compared to the three studies reporting ORs (Supplementary Material File S1/Supplementary Figure S2).

As shown in Supplementary Material File S1, risk of bias by the ROBINS-E tool was low or moderate in most of the studies, and high in two studies; when stratifying estimates by risk of bias, it was shown that estimates of studies with high concern for bias were skewed towards the null (Supplementary Material File S1/Supplementary Figure S3). This observation, along with the large, nationally representative population samples in most studies, adequate control for confounding factors, and estimates bordering large effect sizes, led to an evidence-level upgrade from the default low for observational studies to moderate certainty for the protective effect of persistent PA throughout midlife on incident frailty in older age (Supplementary Material File S1/Supplementary Table S6). Evidence for the protective effect of inclining PA was assessed as low certainty due to unexplained heterogeneity (Supplementary Material File S1/Supplementary Table S7).

## 4. Discussion

This systematic review and meta-analysis synthesized evidence from ten longitudinal cohort studies that found that sustained engagement in PA across midlife is associated with a delayed onset of frailty and slower frailty progression in later life. The pooled meta-analysis indicated that persistently active and increasing activity trajectories were associated with approximately 44% and 38% lower risk of developing frailty through the life course, respectively, compared with persistently low PA. These protective effects were well supported for populations older than 60 years old on several continents, but were less robust for younger persons. These findings highlight the importance of long-term engagement in physical activity across adulthood for frailty prevention. The consistency of the associations across diverse populations suggests that maintaining or increasing activity levels over time may contribute to preserving physiological reserve and functional capacity with aging.

The observed association between PA and frailty is biologically plausible and supported by mechanistic evidence. Frailty is increasingly conceptualized as a state of multisystem physiological dysregulation involving musculoskeletal, neuromuscular, metabolic, inflammatory, neuroendocrine, endothelial and cardiovascular dysfunction, impaired mitochondrial function and chronic low-grade inflammation [7,37,42,43]. Notably, many of these biological pathways are directly modulated by regular aerobic exercise, which was shown to enhance mitochondrial biogenesis and oxidate capacity in skeletal muscle, and improve vascular and endothelial function [44,45,46]. These adaptations are also accompanied by anti-inflammatory effects, with aerobic exercise trials in midlife and older adults showing reductions in key inflammatory markers [47]. Conversely, a status of increased immune activation in midlife, by combining IL-6, TNF-α and fibrinogen concentrations in serum, may be associated with the risk of prefrailty/frailty after 22 years [48]. Collectively, these findings support the hypothesis that PA contributes to the preservation of multisystem physiological resilience rooted at the molecular level.

Within this framework, Seeman and colleagues, building on the allostatic load model originally proposed by McEwen, have shown that higher cumulative stress burden is associated with accelerated functional decline and less favorable aging trajectories [49,50]. Extending this conceptual model with longitudinal population data, Korpisaar et al. recently demonstrated that persistently inactive or decreasing PA trajectories from early adulthood to midlife were associated with higher allostatic load in midlife, reflecting greater multisystem physiological dysregulation. In contrast, individuals who maintained recommended levels of PA across adulthood exhibited lower cumulative stress burden, suggesting that PA may act as a modifier of long-term physiological stress exposure rather than a short-term protective factor alone [51].

Additional mechanistic evidence indicates that physical activity is associated with slower age-related cognitive decline and neurodegenerative processes, which intersect with frailty risk. Higher habitual activity has been associated with preserved hippocampal volume, reduced amyloid accumulation and better executive function in aging populations, suggesting a neuroprotective effect that may mitigate cognitive vulnerability, a recognized component of frailty [52,53,54]. These pathways may help explain why activity patterns established earlier in adulthood, when neuromuscular and neurocognitive systems remain more adaptable, are associated with stronger long-term protective effects.

While previous systematic reviews and meta-analyses have demonstrated a beneficial association between PA and incident frailty, they primarily focused on cumulative activity levels [16,17]. In contrast, the present review specifically synthesizes evidence on longitudinal physical activity patterns and, to our knowledge, is the first age-stratified meta-analysis in which the life stage during which long-term PA patterns were established was examined as a potential effect modifier of the association between PA and frailty. The present review extends prior work by providing an aggregate estimate of the impact of long-term engagement with PA throughout different phases of adulthood on future frailty development and trajectories across distinct stages of adulthood. Overall, the evidence indicates that sustained or increasing PA throughout adulthood was consistently associated with lower frailty risk. Similar effects were observed in studies including populations both younger and older than 60 years. It should be noted, however, that although there is an abundance of evidence for the protective effect of persistent or inclining PA trajectories against frailty development in older adults over age 60, there were very few studies to support such a potential for healthful PA trajectories in middle-aged adults. Notably, data from the ELSA cohort, one of the large population-based cohorts reporting on associations between longitudinal PA patterns and future frailty in middle-aged adults [34], could not be incorporated in the meta-analysis for the impact of persistent PA on future frailty, since separate risk ratios were reported for moderate persistent and vigorous persistent PA, while the rest of the cohorts reported an aggregate risk ratio for both patterns of persistent physical activity.

Within early-older-age cohorts, available evidence suggests that exercise duration may play a more influential role than frequency alone. In the KLoSA cohort, Jung et al. reported that longer exercise sessions (≥30 min per session) were associated with lower risk of incident frailty, whereas higher weekly frequency without sufficient duration conferred more limited benefits. This pattern is supported by independent evidence demonstrating a strong inverse association between exercise duration and frailty. In a recent cross-sectional community-based study, longer daily exercise duration (≥30–60 min/session) was associated with substantially lower frailty risk compared with very short sessions, reinforcing the importance of accumulated exercise volume in later life [55].

### Strengths and Limitations

This review has several notable strengths, including prospective registration in PROSPERO and adherence to the PRISMA 2020 reporting guidelines, with ROBINS-E risk-of-bias assessment and a GRADE approach to ascertain the level of evidence base, ensuring transparency and methodological rigor. It adopts a life-course organizational framework, grouping studies by baseline age to clarify how the timing and continuity of physical activity across adulthood relate to frailty development. Emphasis on long-term PA trajectories and repeated assessments allows sustained behavioral patterns to be examined rather than isolated time-point exposures. In addition, most included studies incorporated a temporal separation between physical activity assessment and frailty evaluation, reducing the likelihood of reverse causation. The inclusion of cohorts from multiple countries and geographic regions further enhances external validity. Moreover, this is the first attempt for an age-stratified meta-analysis comparing effects of PA patterns in those younger and older than 60 years.

Several methodological limitations should also be acknowledged. These can be categorized into limitations of the meta-analytical process, and limitations of the evidence base. In the latter category, it must be acknowledged that all included studies were observational in design, precluding causal inference, and attrition was substantial, especially in longer follow-up studies. The design of most studies does not account for survival bias, is not immune to residual confounding, and cannot exclude reverse causation, especially in studies with close temporal association between the final wave of PA recording and frailty assessment. PA was predominantly self-reported, often using broad or categorical measures that may not fully capture intensity, duration, or type of activity, potentially leading to exposure misclassification. Some studies model PA trajectories over time, while others classify participants as consistently active or inactive, without fully capturing changes in behavior. Although all included studies assessed PA longitudinally, the pooled estimates represent a single summary effect from each study rather than dynamic estimates reflecting changes in physical activity trajectories over time. Future meta-analyses of studies using harmonized longitudinal exposure definitions and formal trajectory modelling would enable truly dynamic pooled estimates, which would be more informative as to the value of midlife physical activity patterns for the prevention of frailty. In addition, heterogeneity in frailty measurement further complicates comparisons across studies. In several cohorts, frailty was assessed at discrete time points, limiting precision in determining the exact onset or progression of frailty. Follow-up duration varied considerably across studies, with shorter follow-up periods more common in older-age cohorts [36,37,39]. Some studies included pre-frail or frail individuals at baseline [32,39], increasing the possibility of reverse causation or survival bias, particularly in very old populations. Attrition was substantial in certain cohorts [32,34,35,39], and excluded participants were often older or had poorer health profiles, which may have led to underestimation of frailty risk. Residual confounding cannot be excluded, as not all studies adjusted for relevant biological or behavioral factors.

Regarding limitations deriving from our review and meta-analysis process, we acknowledge that the small number of included studies, albeit with large, nationally representative populations, limits our ability to explore additional sources of heterogeneity. The robustness of our findings is also challenged by heterogeneity in methodologies for PA and frailty assessments, as well as effect estimate types. Specifically, in order to derive a quantitative synthesis of the data, we had to treat both ORs and HRs as RRs, an assumption that may not be equally robust across all cohorts and outcome definitions. We were able to test concordance of studies reporting ORs to those reporting HRs for the pattern of inclining activity, but were unable to demonstrate similar comparability for the pattern of persistent activity due to the scarcity of studies reporting HRs. Finally, the restriction to English-language publications may have introduced language bias by excluding potentially relevant studies published in other languages. Nevertheless, the convergence of findings across diverse methodological contexts supports the robustness of the observed associations between physical activity patterns and frailty-related outcomes. Overall, these limitations may influence the accuracy of the magnitude, rather than the direction, of observed associations, supporting the conclusion that sustained physical activity in midlife is inversely associated with frailty risk in older age.

## 5. Conclusions

There is moderate-certainty evidence to support middle-aged adults maintaining high levels of physical activity as they progress into older age and low-certainty evidence to support them increasing their levels of physical activity as plausible strategies for frailty prevention in older adults progressing from midlife to older age, with protective effects likely enhanced with longer periods of a physically active lifestyle. These findings support the promotion of sustained long-term physical activity as a plausible strategy for frailty prevention. However, current evidence remains insufficient to determine the optimal type, dose, intensity, or age window at which physical activity may provide the greatest preventive benefit. The majority of available data comes from population-based prospective observational studies involving participants older than 60 years, whereas studies in persons younger than 60 years are scarce. There is a need for additional longitudinal cohort studies in order to document the preventive potential of persistent PA initiated in early midlife, and interventional studies comparing the effectiveness of preventative public health interventions promoting PA from midlife versus later life. Future research should prioritize objective assessments and harmonized definitions of PA exposure and frailty outcomes to clarify the optimal timing, intensity, and duration of activity for frailty prevention across the life course.

## Figures and Tables

**Table 1:** General characteristics of the included studies.

Author, Year	Region, Total Sample (N)		Mean Baseline Age (Years), Sex Distribution	Mean Follow-Up (Years)	Time Points of PA Follow-Up Assessments	PA Assessment	PA Trajectory Entered in Logistic Regression Model	Frailty Assessment Tool	Trajectory Modelling/Statistical Analysis	Outcome	Adjustment for Age, Sex, Co-Morbidities	Adjustment for Additional Confounders
Savela 2013 [33]	Finland	514	48 M	26	-1974 -2000	Self-reported LTPA intensity/frequency	Moderate vs. low High vs. low Persistent vs. low Inclining vs. low Declining vs. low	Modified Fried phenotype	PA categories based on repeated measurements Multinomial logistic regression	Frailty & prefrailty status at end of follow-up	●	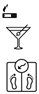
Wang 2022 [34]	UK	8227	58 M ≈ F	10 (waves 4–9)	-2002 -2004 -2006 -2008	Biennial self-report-derived GBTM PA trajectories	Persistently moderate vs. low Persistently vigorous vs. low Inclining vs. low Declining vs. low	Frailty Index (32 items)	GBTM; linear mixed model; Cox proportional hazards regression	Motor function decline Incident frailty	●	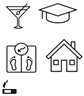 Health condition, depression, ethnicity
Borda 2020 [36]	Mexico	6087	62 M ≈ F	3	2010–2012	Self-reported single answer on PA frequency (3+/week in last 2 years)	Frequent vs. infrequent	Frailty Index (39 items)	PA categories based on self-reported sustained longitudinal PA Multivariable logistic regression	Incident frailty	●	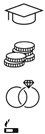 Vaccination, physician visits, breast and prostate cancer screening
Fung 2023 [41]	USA	69,642	≥60 F	24	-1992 -1996 -2000 -2004 -2008 -2012	Self-reported PA h/week; cumulative averages & PA change	High cumulative vs. low cumulative Moderate persistent vs. low Vigorous persistent vs. low Inclining vs. low Declining vs. low	FRAIL scale	PA categories based on repeated measurements; multivariable Cox proportional hazards regression	Incident frailty	●	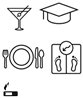 Lean mass, medication use
Lin 2022 [38]	Taiwan	5131	66 M ≈ F	20	-1993 -1996 -1999 -2003 -2007 -2011 -2015	Self-report-derived GBTM PA trajectories (every 3–4 years)	Persistent vs. low Inclining vs. low Declining vs. low	Frailty Index (cumulative deficits across 6 domains—max score = 66)	GBTM; multivariable logistic regression	Incident frailty	●	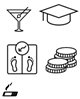 Oral health, well-being, depression
Jung 2025 [40]	Korea	5594	64 M ≈ F	16	-2006 -2008 -2010 -2012 -2014 -2016 -2018 -2020 -2022	Biennial self-reported PA frequency ≥ 1/week	Persistent vs. low Inclining vs. low Declining vs. low Longer vs. shorter duration	34-item Frailty Index	Multivariable logistic regression	Incident frailty	Age and sex only	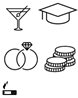 Region, employment, social activities.
Haapanen 2024 [32]	Finland	2003	62 M ≈ F	17	-2001 -2011 -2017	Binary PA Self-report (WHO ≥ 12.5 MET-h/week)	Declining vs. low	37-item Frailty Index	Linear mixed effects models	Frailty progression (β over time)	Age and sex only	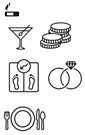 Sleep quality
Huang 2025 [35]	England	4379	67 M ≈ F	3.5	-2018–19 -2021–22	Self-reported weekly frequency of vigorous, moderate, or mild activities	Moderate persistent vs. discontinuous Vigorous persistent vs. discontinuous Inclining to vigorous vs. discontinuous Declining vs. discontinuous	Frailty Index (32 items)	Multivariable logistic regression	Incident frailty	●	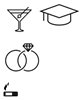 Ethnicity
Hayashi 2021 [39]	Japan	2567	74 M < F	2.4	-2010 -2011 -2012 -2013 -2014 -2015 -2016 -2017 -2018 -2019	Objective recording of community program participation	Mid-term participation vs. short term Long-term participation vs. short term	Kihon Checklist (KCL)	Linear mixed-effects regression models	KCL score increase	Age, sex, and BP only	Pulse rate, baseline KCL
Zhang 2021 [37]	China	7006	81 M ≈ F	3.1 (median)	-2008	Self-reported past and current aerobic exercise	Persistent vs. non-persistent	34-item Frailty Index	Cox proportional hazards regression Subgroup analysis for nonagenarians	Incident frailty	●	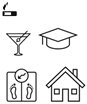 High protein intake

BP: blood pressure; β: regression coefficient (beta coefficient); F: female; FRAIL: fatigue, resistance, ambulation, illnesses, and loss of weight scale; GBTM: group-based trajectory modeling; KCL: Kihon Checklist; LTPA: leisure-time physical activity; M; male; MET: metabolic equivalent of task; PA: physical activity; WHO: World Health Organization. ● = yes. 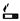: smoking. 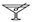: alcohol. 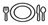:diet 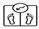: body mass index. 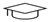: education. 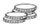: financial status. 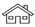: habitat. 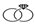: marital status.

**Table 2:** Methodological strengths and limitations of included studies.

Strengths	Savela et al. [33]	Wang et al. [34]	Borda et al. [36]	Fung et al. [41]	Lin et al. [38]	Jung et al. [40]	Haapanen et al. [32]	Hayashi et al. [39]	Zhang et al. [37]	Huang et al. [35]
Nationally representative cohort		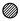	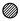		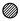	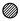	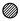		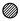	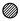
Validated frailty assessment tool	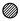	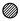	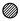	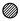	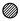	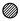	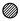	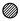	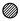	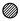
Prospective frailty incidence design	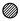	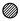	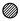	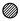	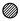	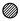			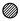	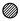
Repeated PA assessments	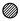	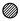		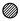	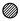	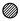	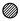	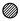		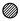
Trajectory modelling (e.g., GBTM)	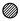	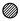			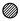	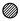				
Objective physical activity or fitness measurement								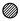		
Long follow-up (≥10 years)	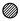	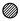		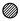	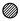	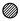	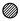			
Clear temporal separation between final PA exposure and frailty outcome assessment		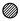	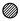	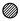					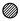	
Limitations	Savela et al.	Wang et al.	Borda et al.	Fung et al.	Lin et al.	Jung et al.	Haapanen et al.	Hayashi et al.	Zhang et al.	Huang et al.
Self-reported PA	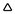	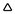	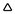	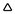	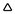	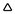	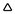		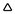	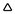
Retrospective PA assessment-recall bias			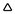						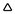	
Limited data on type, intensity, duration of exercise	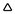	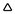	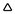		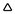	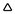			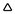	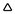
Binary exposure classification			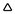				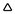		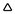	
Short follow-up (<5 years)			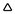					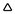	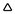	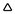
Limited generalizability (sex- or occupation-restricted sample)	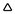			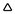						
Potential reverse causation	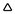	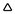			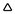	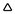	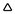	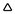	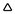	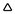
High attrition		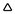				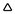	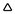	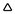		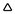
Survival bias	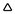	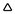					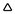	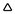		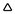
Residual confounding		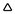			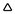		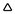	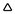	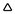	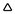
Overall risk of bias	▨	▨	■	▨	▨	▨	□	▨	■	▨

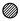
 = strength present; 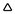 = limitation present; GBTM = group-based trajectory modeling. Overall risk of bias (derived from ROBINS-E overall judgment): □ = low; ▨ = some concerns; ■ = high.

## Data Availability

No new data were created or analyzed in this study. Data sharing is not applicable to this article.

## Supplementary Materials

The following supporting information can be downloaded at: https://www.mdpi.com/article/10.3390/geriatrics11040104/s1, Table S1: Prisma 2020 checklist; Table S2: Search strategy of the study; Table S3: Title screening; Table S4: Abstract screening; Table S5: Longitudinal Physical Activity Assessment Characteristics; Table S6: Detailed ROBINS-E domain-level risk of bias assessments for each included study; Table S7: GRADE assessment of the certainty of evidence delivered in the meta-analysis for the impact of persistent physical activity through midlife on risk of future frailty development; Figure S1. Persistent/consistent PA vs low BY Metric; Figure S2. Inclining/increasing PA vs low by metric; Figure S3. Persistent/consistent PA vs low BY ROB.
