# Supplementary material for: Long-Term Physical Activity Patterns and Risk of Future Frailty: A Systematic Review and Age-Stratified Meta-Analysis
Source: Geriatrics (Basel). 2026 Aug 11;11(4):104. doi: 10.3390/geriatrics11040104 (PMC13512540; doi:10.3390/geriatrics11040104)
Supplement: Supplementary file 1 [file geriatrics-11-00104-s001.zip › geriatrics-4361258-supplementary.pdf]

Supplementary Material File S1

Supplementary Table S1. Prisma 2020 checklist

Section and Topic	Item #	Checklist item	Location where item is reported
**TITLE**			
Title	1	Identify the report as a systematic review.	Title / pages 1
**ABSTRACT**			
Abstract	2	See the PRISMA 2020 for Abstracts checklist.	Abstract /page 2; table S1
**INTRODUCTION**			
Rationale	3	Describe the rationale for the review in the context of existing knowledge.	Introduction/page 2
Objectives	4	Provide an explicit statement of the objective(s) or question(s) the review addresses.	Introduction/page 2
**METHODS**			
Eligibility criteria	5	Specify the inclusion and exclusion criteria for the review and how studies were grouped for the syntheses.	Methods/page 3-4
Information sources	6	Specify all databases, registers, websites, organisations, reference lists and other sources searched or consulted to identify studies. Specify the date when each source was last searched or consulted.	Methods/page 3
Search strategy	7	Present the full search strategies for all databases, registers and websites, including any filters and limits used.	SM_2
Selection process	8	Specify the methods used to decide whether a study met the inclusion criteria of the review, including how many reviewers screened each record and each report retrieved, whether they worked independently, and if applicable, details of automation tools used in the process.	Methods/page 3
Data collection process	9	Specify the methods used to collect data from reports, including how many reviewers collected data from each report, whether they worked independently, any processes for obtaining or confirming data from study investigators, and if applicable, details of automation tools used in the process.	Methods/page 4
Data items	10a	List and define all outcomes for which data were sought. Specify whether all results that were compatible with each outcome domain in each study were sought (e.g. for all measures, time points, analyses), and if not, the methods used to decide which results to collect.	Methods/Page 4
	10b	List and define all other variables for which data were sought (e.g. participant and intervention characteristics, funding sources). Describe any assumptions made about any missing or unclear information.	Methods/Page 4
Study risk of bias assessment	11	Specify the methods used to assess risk of bias in the included studies, including details of the tool(s) used, how many reviewers assessed each study and whether they worked independently, and if applicable, details of automation tools used in the process.	Methods / pages 4/ table S6
Effect measures	12	Specify for each outcome the effect measure(s) (e.g. risk ratio, mean difference) used in the synthesis or presentation of results.	Page 4
Synthesis methods	13a	Describe the processes used to decide which studies were eligible for each synthesis (e.g. tabulating the study intervention characteristics and comparing against the planned groups for each synthesis (item #5)).	Methods (Eligibility Criteria, Data Charting Process, Statistical Analysis) / pages 3–4

Section and Topic	Item #	Checklist item	Location where item is reported
	13b	Describe any methods required to prepare the data for presentation or synthesis, such as handling of missing summary statistics, or data conversions.	Methods (Data Charting Process, Statistical Analysis) / page 4
	13c	Describe any methods used to tabulate or visually display results of individual studies and syntheses.	Methods (, Statistical Analysis) / pages 4-5)
	13d	Describe any methods used to synthesize results and provide a rationale for the choice(s). If meta-analysis was performed, describe the model(s), method(s) to identify the presence and extent of statistical heterogeneity, and software package(s) used.	Methods (, Statistical Analysis) / pages 4-5)
	13e	Describe any methods used to explore possible causes of heterogeneity among study results (e.g. subgroup analysis, meta-regression).	Methods (statistical Analysis)/page 5
	13f	Describe any sensitivity analyses conducted to assess robustness of the synthesized results.	Methods (statistical Analysis)/page 5
Reporting bias assessment	14	Describe any methods used to assess risk of bias due to missing results in a synthesis (arising from reporting biases).	Methods/ page 4
Certainty assessment	15	Describe any methods used to assess certainty (or confidence) in the body of evidence for an outcome.	Methods/ page 4
**RESULTS**			
Study selection	16a	Describe the results of the search and selection process, from the number of records identified in the search to the number of studies included in the review, ideally using a flow diagram.	Fig. 1/Page 5
	16b	Cite studies that might appear to meet the inclusion criteria, but which were excluded, and explain why they were excluded.	Results/ Page 6
Study characteristics	17	Cite each included study and present its characteristics.	Results/ Pages 6-10/ Table 1
Risk of bias in studies	18	Present assessments of risk of bias for each included study.	Results /page 11 table S6
Results of individual studies	19	For all outcomes, present, for each study: (a) summary statistics for each group (where appropriate) and (b) an effect estimate and its precision (e.g. confidence/credible interval), ideally using structured tables or plots.	Results/pages 12-14 / table 1 & figures 2,3
Results of syntheses	20a	For each synthesis, briefly summarise the characteristics and risk of bias among contributing studies.	Results/ pages 12
	20b	Present results of all statistical syntheses conducted. If meta-analysis was done, present for each the summary estimate and its precision (e.g. confidence/credible interval) and measures of statistical heterogeneity. If comparing groups, describe the direction of the effect.	Results/ page 14-16
	20c	Present results of all investigations of possible causes of heterogeneity among study results.	Results/ page 14-16; fig.2-3
	20d	Present results of all sensitivity analyses conducted to assess the robustness of the synthesized results.	Supplementary fig.
Reporting biases	21	Present assessments of risk of bias due to missing results (arising from reporting biases) for each synthesis assessed.	Supplementary fig.
Certainty of evidence	22	Present assessments of certainty (or confidence) in the body of evidence for each outcome assessed.	Supplementary material/ S_7
**DISCUSSION**			

Section and Topic	Item #	Checklist item	Location where item is reported
Discussion	23a	Provide a general interpretation of the results in the context of other evidence.	Discussion/ page 18-19
	23b	Discuss any limitations of the evidence included in the review.	Discussion /page 19/ table 2
	23c	Discuss any limitations of the review processes used.	Discussion/Page 19
	23d	Discuss implications of the results for practice, policy, and future research.	Conclusion/ page 20
**OTHER INFORMATION**			
Registration and protocol	24a	Provide registration information for the review, including register name and registration number, or state that the review was not registered.	Methods /Page 3
	24b	Indicate where the review protocol can be accessed, or state that a protocol was not prepared.	PROSPERO (registration number: CRD420251168483)
	24c	Describe and explain any amendments to information provided at registration or in the protocol.	N/A
Support	25	Describe sources of financial or non-financial support for the review, and the role of the funders or sponsors in the review.	Statements/declarations/page 17
Competing interests	26	Declare any competing interests of review authors.	Statements/declarations/page 17
Availability of data, code and other materials	27	Report which of the following are publicly available and where they can be found: template data collection forms; data extracted from included studies; data used for all analyses; analytic code; any other materials used in the review.	N/A

From: Page MJ, McKenzie JE, Bossuyt PM, Boutron I, Hoffmann TC, Mulrow CD, et al. The PRISMA 2020 statement: an updated guideline for reporting systematic reviews. BMJ 2021;372:n71. doi: 10.1136/bmj.n71.
This work is licensed under CC BY 4.0. To view a copy of this license, visit https://creativecommons.org/licenses/by/4.0/

Supplementary Table S2. Search strategy of the study

PubMed

Search terms	Search field
"Exercise"[MeSH]	MeSH Terms
"Motor Activity"[MeSH]	MeSH Terms
"physical activity"	Title/Abstract
"Exercise"	Title/Abstract
"aerobic exercise"	Title/Abstract
"resistance exercise"	Title/Abstract
"strength exercise"	Title/Abstract
"isometric exercises"	Title/Abstract
"daily activity"	Title/Abstract
"daily physical activity"	Title/Abstract
"leisure activity"	Title/Abstract
"leisure-time physical activity"	Title/Abstract
"activity level*"	Title/Abstract
"Trajectories"	Title/Abstract
"Patterns"	Title/Abstract
"Long-Term"	Title/Abstract
"Longitudinal"	Title/Abstract
"incidence"	Title/Abstract
"growth curve*"	Title/Abstract
"follow-up"	Title/Abstract

"prospective"	Title/Abstract
"cohort"	Title/Abstract
"Frailty"[MeSH]	MeSH Terms
"Frail Elderly"[MeSH]	MeSH Terms
"Frailty Syndrome"	Title/Abstract
"frailty risk"	Title/Abstract
"frailty incidence"	Title/Abstract

Search combination (PubMed)

Step	Query
1	1–13 OR
2	14–22 OR
3	23–27 OR
4	1 AND 2 AND 3
5	Filters: English language; records indexed from database inception to June 2025

Web of Science

Search terms	Search field
"Exercise"	Topic (TS)
"Motor Activity"	Topic (TS)
"physical activity"	Topic (TS)
"aerobic exercise"	Topic (TS)
"resistance exercise"	Topic (TS)
"strength exercise"	Topic (TS)
"isometric exercises"	Topic (TS)
"daily activity"	Topic (TS)

"daily physical activity"	Topic (TS)
"leisure activity"	Topic (TS)
"leisure-time physical activity"	Topic (TS)
"activity level*"	Topic (TS)
"Trajectories"	Topic (TS)
"Patterns"	Topic (TS)
"Long-Term"	Topic (TS)
"Longitudinal"	Topic (TS)
"incidence"	Topic (TS)
"growth curve*"	Topic (TS)
"follow-up"	Topic (TS)
"prospective"	Topic (TS)
"cohort"	Topic (TS)
"Frailty"	Topic (TS)
"Frail Elderly"	Topic (TS)
"Frailty Syndrome"	Topic (TS)
"frailty risk"	Topic (TS)
"frailty incidence"	Topic (TS)

Search combination (Web of Science)

Step	Query
1	1–13 OR
2	14–22 OR
3	23–26 OR
4	1 AND 2 AND 3

5	Filters: English language; records indexed from database inception to June 2025

Embase

Search terms	Search field
kinesiotherapy	Emtree Terms
motor activity	Emtree Terms
physical activity	Title/Abstract
exercise	Title/Abstract
aerobic exercise	Title/Abstract
resistance exercise	Title/Abstract
strength exercise	Title/Abstract
isometric exercise	Title/Abstract
daily activity	Title/Abstract
daily physical activity	Title/Abstract
leisure activity	Title/Abstract
leisure time physical activity	Title/Abstract
activity level*	Title/Abstract
trajectory	Title/Abstract
trajectories	Title/Abstract
pattern*	Title/Abstract
long term	Title/Abstract
longitudinal	Title/Abstract

incidence	Title/Abstract
growth curve*	Title/Abstract
follow up	Title/Abstract
prospective	Title/Abstract
cohort	Title/Abstract
frailty	Emtree Terms
frail elderly	Emtree Terms
frailty syndrome	Title/Abstract
frailty risk	Title/Abstract
frailty incidence	Title/Abstract

Search combination (Embase)

Step	Query
1	1–13 OR
2	14–23 OR
3	24–28 OR
4	1 AND 2 AND 3
5	Filters: English language; records indexed from database inception to June 2025

Supplementary Table S3. Title screening

Code	Exclusion category	Includes	Items
1	Irrelevant topic	Irrelevant exposure, outcome, intervention, or research question	3543
2	Wrong outcome	Frailty not assessed as the outcome	256
3	Disease-specific population	Cancer, heart failure, Parkinson's disease, CKD, HIV etc.	487
4	Institutionalized population	Nursing homes, hospitalized patients, long-term care facilities	66
5	Wrong exposure	Physical activity not evaluated as the exposure of interest	202
6	Interventional studies	Randomized controlled trials, non-randomized intervention studies, controlled clinical trials, experimental studies**, pilot studies/trials, trial protocols/study protocols**	345
7	Cross-sectional studies	Cross-sectional and other ineligible study design	9
8	Reviews	Systematic reviews, meta-analyses, narrative reviews, scoping reviews	63
9	Conference abstracts	Conference proceedings, congress abstracts, meeting abstracts	12
10	Case reports	Case reports and case series	1
11	Animal studies	Animal and preclinical studies	16
12	Wrong population	Children, adolescents, or other ineligible populations	10
13	Retracted articles	Retracted publications	5
		Total excluded articles	5015

Supplementary Table S4. Abstract screening

Code	Exclusion category	Includes	Items
2	Wrong outcome	Frailty not assessed as the outcome	6
3	Disease-specific population		1
5	Wrong exposure	Physical activity not evaluated as the exposure of interest	12
6	Interventional studies	Randomized controlled trials, non-randomized intervention studies, controlled clinical trials, experimental studies, pilot studies/trials, and exercise-based intervention studies	18
7	Wrong study design	Cross-sectional studies and other ineligible study designs	25
8	Reviews	Systematic reviews, meta-analyses, narrative reviews, and scoping reviews	6
9	Conference abstracts	Conference proceedings, congress abstracts, and meeting abstracts	6
14	Short-term exposure	Studies assessing PA only at baseline or the PA exposure was very short-term rather than long-term or repeated exposure	8
		Total excluded articles	82

Supplementary Table S5. Longitudinal Physical Activity Assessment Characteristics

Study	PA time points	Exposure window	Activity definition	Type of activity	Reference group	Trajectory modelling
Savela et al., 2013	1974, 2000	1974–2000 (26-year PA exposure)	Self-reported leisure-time PA intensity/frequency	Leisure-time PA (walking, cycling, gardening, jogging, swimming, tennis, skiing, etc.)	Low PA	No formal trajectory modelling (activity-change categories)
Wang et al., 2022	2002, 2004, 2008, 2012	2002–2012 (10-year repeated PA exposure)	Self-reported frequency of mild, moderate and vigorous PA	Mixed-intensity PA: mild (laundry, home repairs); moderate (gardening, brisk walking, dancing, stretching); vigorous (running, swimming, cycling, aerobics/gym, tennis, digging)	Low PA	GBTM

Haapanen et al., 2024	2001–2004, 2011–2013, 2017–2018	2001–2018 (17-year repeated PA exposure)	Self-reported regular exercise according to WHO recommendation	Moderate-to-vigorous leisure-time PA (≥12.5 MET-h/week)	Maintained activity	No formal trajectory modelling (change-score analysis)
Borda et al., 2020	2012, 2015	Previous 2 years	Self-reported PA or hard physical work ≥3 times/week	Non-specific physical activity (exercise or hard physical work)	Infrequent PA	No formal trajectory modelling
Lin et al., 2022	1996, 1999, 2003, 2007, 2011, 2015	1996–2015 (20-year repeated PA exposure)	Self-reported exercise frequency	Non-specific leisure-time exercise frequency	Low PA	GBTM
Fung et al., 2023	1992, 1996, 2000, 2004, 2008, 2012	1992–2012 (20-year repeated PA exposure)	Repeated MET-hours/week of total, moderate and vigorous PA	Total, moderate and vigorous leisure-time PA (walking, running, swimming, cycling, calisthenics, aerobics, racquet sports, yoga,	Lowest cumulative PA	No formal trajectory modelling (repeated longitudinal assessment)

				weight training, etc.)		
Jung et al., 2025	2006, 2008, 2010, 2012, 2014, 2016, 2018, 2020, 2022	2006–2022 (16-year repeated PA exposure)	Self-reported changes in regular exercise (≥1 session/week)	Non-specific physical activity	Persistently inactive	No formal trajectory modelling (LAG-defined change groups)
Huang et al.,2025	2008-2009<br><br>2012-2013	2008-2012<br><br>(3,5 years repeated PA exposure	Self-reported frequency of mild, moderate and vigorous PA	Mild activities (laundry and home repairs);<br><br>moderate-intensity activity (gardening, cleaning<br><br>the car, walking at moderate pace, dancing, and floor or<br><br>stretching exercises); and vigorous intensity (run	Persistently inactive	No formal trajectory modelling (activity-change categories)

				ning/ jogging, swimming, cycling, aerobics/gym workout, tennis, and digging with a spade)		
Hayashi et al., 2021	Repeated participation records (2010–2019)	2010–2019 (up to 6,4 years of exercise programme participation)	Duration of participation in community resistance exercise	Community-based group resistance training (arm raises, sit-to-stand, knee extension, knee lift, lateral leg lift, hip extension)	Short-term participation	No formal trajectory modelling (MLM for longitudinal outcome modelling)
Zhang et al., 2021	Baseline	2008 (past and current exercise assessed at baseline)	Persistent aerobic exercise	Aerobic exercise (walking, running, ball games, etc.)	Non-persistent aerobic exercise	No formal trajectory modelling

Supplementary Table S6. Detailed ROBINS-E domain-level risk of bias assessments for each included study.

The Risk Of Bias In Non-randomized Studies – of Exposures (ROBINS-E) assessment tool
(for follow-up studies)

Version 24 March 2024

This work is licensed under a Creative Commons Attribution-NonCommercial-NoDerivatives 4.0 International License.

The ROBINS-E tool

At planning stage: list confounding factors and consider appropriateness criteria

P1. List the important confounding factors relevant to all or most studies on this topic. Specify whether these are particular to specific exposures-outcome combinations.

Important potential confounders included age, sex, socioeconomic status (education), body mass index (BMI), smoking, alcohol consumption, baseline physical function, baseline frailty status, multimorbidity/chronic diseases (e.g., hypertension, diabetes, cardiovascular disease, stroke, cancer, chronic lung disease), depressive symptoms, functional limitations, and other lifestyle or health-related factors that may influence both physical activity and the risk of frailty.

P2. Will the review use the ROBINS-E assessment of appropriateness (important aspects of "study sensitivity")?

No

If Yes, complete sections Addressing appropriateness, Parts I and II in Appendix 1.

Savela et al.

For each study result: preliminary considerations

A. Specify the result being assessed for risk of bias

A1. Specify the numerical result being assessed

Association between midlife leisure-time physical activity (1974) and frailty status in old age (2000) after 26 years of follow-up, estimated using adjusted logistic regression models.

B. Decide whether to proceed with a risk-of-bias assessment

	Response options	Comments
B1. Did the authors make any attempt to control for confounding?	Y	Multivariable logistic regression models were used. The authors adjusted for age, body mass index, smoking, blood pressure, cholesterol, alcohol consumption, and additionally for Charlson comorbidity index.
B2. **If N/PN to B1**: Is there sufficient potential for confounding that an unadjusted result should not be considered further?	NA	NA
B3. Was the method of measuring exposure inappropriate?	N	Leisure-time physical activity was self-reported in 1974 before outcome occurrence. Although self-report may introduce non-differential misclassification, the exposure assessment was appropriate for a prospective cohort study.
B4. Was the method of measuring the outcome inappropriate?	N	Frailty was assessed at follow-up using a modified Fried phenotype with predefined criteria applied to all participants.

If the answer to any of B2, B3 or B4 is 'Yes' or 'Probably yes', the result should be considered to be at very high risk of bias and no further assessment is required. Otherwise, proceed to section C.

C. Specify the analysis in the current study for which results are being assessed for risk of bias

C1. Specify the outcome to which this result relates.

Incident frailty at the 26-year follow-up (2000).

C2. Specify the participant group on which this result was based.

Initially healthy, community-dwelling Caucasian men from the Helsinki Businessmen Study who participated in both baseline (1974) and follow-up (2000) assessments (n = 514).

C3 to C8: Describe the exposure measurement(s) used to produce this result.

C3. What is the exposure being measured and how was it measured or assessed?	Leisure-time physical activity (LTPA) in midlife, assessed in 1974 using a self-administered questionnaire. Participants were classified into low, moderate, and high LTPA categories according to weekly exercise frequency and intensity.
C4. Was exposure analysed as a quantitative (rather than a categorical) variable?	N
C5. Did repeated measurements of exposure over time (for each participant) contribute to the analysis that produced this result?	N
C6. **If Y/PY to C5**, was a single estimate of each participant's exposure level derived from the repeated measurements of exposure over time?	NA
C7. **If N/PN to C6**, was the analysis based on splitting participants' follow up time according to exposure status and/or magnitude?	NA
C8. **If Y/PY to C7**, were changes in exposure status and/or magnitude likely to be related to factors that are predictive of the outcome?	NA

C9. **If N/PN to C7**, how were repeat measurements used?

Y = Yes; PY = Probably yes; PN = Probably no; N = No; NA = Not applicable

C10. Specify the relationship analysed to produce this result. For example, this may be a quadratic relationship of cumulative exposure with the log odds of the outcome, or a risk ratio for the outcome comparing exposed with unexposed individuals.

Association between categorical midlife leisure-time physical activity (low, moderate and high) and frailty status at 26-year follow-up, estimated using multivariable logistic regression models, with the low physical activity group as the reference category.

D: Specify the causal effect of exposure being estimated by this result

D1. Specify the population of interest	Describe eligible participants (to whom the causal effect applies). These may be different from the study participants on whom the result was based (specified in C2). Such differences may give rise to selection biases.	514 initially healthy community-dwelling Caucasian middle-aged men from the Helsinki Businessmen Study, free of frailty at baseline
Specification of the exposure metric of interest		
D2. Specify the exposure	This is the factor whose causal effect on the outcome of interest is the subject of the study result being assessed. It may be thought of as the 'true' exposure of interest. It is distinct from the method with which exposure was measured.	Leisure-time physical activity in midlife.
D3. Specify the exposure window	The exposure window of interest is the exposure period for which the result being assessed estimates the effect of exposure on the outcome. Specification of the exposure window is judged by the ROBINS-E user, who should aim to define a window that is both meaningful in answering the review question and broadly in line with when the study measured exposure. Specification should include both the time of onset and period of exposure. For example, it may be lifetime exposure (from birth or from conception), during ages 50-55, the period from first employment in a particular occupation, time from birth to age 10, or during pregnancy.<br><br>The specified exposure window is used to determine whether exposure data adequately reflect exposure during the window. Exposure before the start of the exposure window is addressed during the assessment of risk of bias due to confounding	Midlife (approximately 48 years of age, assessed at baseline in 1974).
D4. Specify how exposure over time	This may, for example, be ever/never exposed, cumulative exposure, average exposure, or peak exposure during the exposure period, for each participant. Alternatively, there may be only a	Categorical baseline exposure (low, moderate, and high leisure-time physical activity).

should be summarized	single exposure event, or the exposure may be time invariant (such as a genetic variant or family history).	

E. Evaluation of confounding factors

Complete a row for each important confounding factor listed in advance (subsection (i)). In addition, consider any further confounding factors that are either relevant to the setting of this particular study or which the study authors identified as potentially important (subsection (ii)).

"Important" confounding factors are those for which, in the context of this study, adjustment is expected to lead to an important change in the estimated effect of the exposure.

(i) Important confounding factors listed in advance						
Confounding factor	Measured variable(s) for this factor, if any	Was this variable (or were these variables) controlled for in the analysis?<br>(Y / N)	If this confounding factor was controlled for, was it measured validly and reliably by this variable (or these variables)?*<br>(NA / Y / PY / PN / N / NI)	If this confounding factor was not controlled for, is there evidence that controlling for it was unnecessary?**<br>(NA / Y / PY / PN / N)	Is failure to adjust for this confounding factor expected to bias the effect estimate towards benefit or harm of (higher) exposure?***<br>(Benefit of (higher) exposure / Harm of (higher) exposure / Insufficient information available)	Comments
Age	years	Y	Y	NA	NA	Adjusted in all multivariable models
Sex	Male/female	Y	N	Y	NA	All participants were men; sex could not confound within this cohort.

BMI	Body mass index	Y	Y	NA	NA	Adjusted in multivariable models
Baseline health/comorbidities	Blood pressure, cholesterol and Charlson comorbidity index	Y	Y	NA	NA	Blood pressure, cholesterol and Charlson comorbidity index were included in adjusted models.
Baseline frailty / functional status	Self-rated health/fitness	N	PN	N	Benefit of higher exposure	No formal baseline frailty assessment.
Lifestyle factors	Smoking & alcohol consumption	Y	Y	N	NA	Included in adjustment
Socioeconomic status	Businessman cohort	N	N	PY	NA	Cohort consisted of businessmen with relatively homogeneous socioeconomic status; SES was therefore not adjusted for.

(ii) Additional confounding factors relevant to the setting of this particular study, or identified by study authors and considered to be important, or which were identified since the protocol was written

Confounding factor	Measured variable(s) for this factor, if any	Was this variable (or were these variables) controlled for in the analysis?<br>(Y / N)	If this confounding factor was controlled for, was it measured validly and reliably by this variable (or these variables)?*<br>(NA / Y / PY / PN / N / NI)	If this confounding factor was not controlled for, is there evidence that controlling for it was unnecessary?**<br>(NA / Y / PY / PN / N)	Is failure to adjust for this confounding factor expected to bias the effect estimate towards benefit or harm of (higher) exposure?*** (Benefit of (higher) exposure / Harm of (higher) exposure / Insufficient information available)	Comments

* "Validity" refers to whether the confounding variable or variables accurately measure the confounding factor, while "reliability" refers to the precision of the measurement (more measurement error means less reliability).

** In the context of a particular study, variables need not be included in the analysis: (a) if they are measured validly and reliably and are not associated with the outcome, conditional on exposure (noting that lack of a statistically significant association is not evidence of a lack of association); (b) if they are measured validly and reliably and are not associated with exposure; (c) if they are measured validly and reliably and adjustment makes no or minimal difference to the estimated effect of the primary parameter; (d) because the confounder was addressed in the study design, for example by restricting to individuals with the same value of the confounder; (e) because a negative control demonstrates that there was unlikely to have been confounding due to this variable or that uncontrolled confounding was likely to be minimal; or (f) because external evidence suggests that controlling for the variable is not necessary in the context of the study being assessed..

For each study: risk of bias assessment

Domain 1: Risk of bias due to confounding

*Domain 1, Variant (a): **If N/PN to C5 or Y/PY to C6 or N/PN to C7** (only baseline confounding needs to be addressed)*

Signalling questions	Response options	Comments
1.1 Did the authors control for all the important confounding factors for which this was necessary?	PY	The analyses adjusted for major baseline confounders including age, BMI, smoking, alcohol consumption, blood pressure, cholesterol and Charlson comorbidity index. However, baseline frailty or functional status was not directly assessed, leaving the possibility of residual confounding.
1.2 **If Y/PY/WN to 1.1**: Were confounding factors that were controlled for (and for which control was necessary) measured validly and reliably by the variables available in this study?	PY	Most confounders were measured objectively. However, baseline health status relied partly on self-reported information and baseline frailty was not directly assessed.
1.3 **If Y/PY/WN to 1.1**: Did the authors control for any variables after the start of the exposure period being studied that could have been affected by the exposure?	Y	Model C additionally adjusted for Charlson comorbidity index assessed at follow-up (2000), which may lie on the causal pathway between midlife physical activity and frailty.
1.4 Did the use of negative controls, or other considerations, suggest serious uncontrolled confounding?	N	No negative controls were reported
Risk of bias (due to confounding) in the estimated effect of exposure on the outcome	Some concerns	Most important baseline confounders were adjusted for. However, baseline frailty or functional status was not directly assessed, leaving the possibility of residual confounding.
What is the predicted direction of bias due to confounding?	Towards benefit of (higher) exposure	Residual confounding is likely to overestimate the protective effect of higher physical activity, as healthier participants may have been more likely to engage in higher levels of leisure-time physical activity.
Is the risk of bias (due to confounding) sufficiently high, in the context of its likely direction and the magnitude of the estimated exposure effect, to threaten conclusions about whether the exposure has an important effect on the outcome?	No	Although some residual confounding cannot be excluded, the adjustment for major baseline confounders is unlikely to invalidate the overall study conclusions.

Y = Yes; PY = Probably yes; PN = Probably no; N = No; SY = Strong yes; WY = Weak yes; SN = Strong no; WN = Weak no; NA = Not applicable; NI = No information

*Domain 1, variant (b): **If Y/PY to C7 and Y/PY to C8** (the analysis was based on splitting participants' follow up time according to exposure status and/or magnitude and changes in exposure status and/or magnitude likely to be related to factors that are predictive of the outcome, so both baseline and time-varying confounding need to be addressed)*

Signalling questions	Response options	Comments
1.1 Did the authors use an analysis method that was appropriate to control for time-varying as well as baseline confounding?	Y / PY / PN / N / NI	
1.2 **If Y/PY to 1.1**: Did the authors control for all the important baseline and time-varying confounding factors for which this was necessary?	NA / Y / PY / WN (no, but uncontrolled confounding was probably not substantial) / SN (no, and uncontrolled confounding was probably substantial) / NI	
1.3 **If Y/PY/WN to 1.2**: Were confounding factors that were controlled for (and for which control was necessary) measured validly and reliably by the variables available in this study?	NA / Y / WN (no, but the extent of measurement error in confounding factors was probably not substantial) / SN (no, and the extent of measurement error in confounding factors was probably substantial) / NI	
1.4 **If N/PN/NI to 1.1**: Did the authors control for time-varying factors or other variables measured after the start of the exposure window being studied?	NA / Y / PY / PN / N / NI	
1.5 Did the use of negative controls, or other considerations, suggest uncontrolled confounding?	Y / PY / PN / N	
Risk of bias (due to confounding) in the estimated effect of exposure on the outcome	Low risk / Some concerns / High risk / Very high risk	

Signalling questions	Response options	Comments
What is the predicted direction of bias due to confounding?	Towards benefit of (higher) exposure / Towards harm of (higher) exposure / Towards null / Away from null / Insufficient information available	
Is the risk of bias (due to confounding) sufficiently high, in the context of its likely direction and the magnitude of the estimated exposure effect, to threaten conclusions about whether the exposure has an important effect on the outcome?	Yes / No / Cannot tell	

Y = Yes; PY = Probably yes; PN = Probably no; N = No; SY = Strong yes; WY = Weak yes; SN = Strong no; WN = Weak no; NA = Not applicable; NI = No information

Domain 2: Risk of bias arising from measurement of the exposure

Domain 2, Variant (a): ***If N/PN to C5*** *(exposure was measured at a single point in time)*

Signalling questions	Response options	Comments
Mismeasurement or misclassification of the exposure.		
2.1 Does the measured exposure well-characterize the exposure metric specified to be of interest in this study? [*This was specified in the answers to D2, D3 and D4*]	PY	Leisure-time physical activity was assessed prospectively at baseline using a standardized questionnaire and categorized into three predefined activity levels.
2.2 Was the exposure likely to be measured with error, or misclassified?	SY	Physical activity was self-reported, which may have resulted in some non-differential misclassification of exposure.
Bias in the estimated effect of exposure arising from mismeasurement or misclassification of the exposure		
2.3 If SY/WY to 2.2: Could mismeasurement or misclassification of exposure have been differential (i.e. related to the outcome or risk of the outcome)?	PN	Exposure was assessed approximately 26 years before frailty assessment; therefore, measurement error was unlikely to depend on the future outcome.
2.4 If SY/WY to 2.2 and N/PN/WY to 2.3: Is non-differential measurement error likely to bias the estimated effect of exposure on outcome?	WY	Non-differential misclassification would most likely attenuate the association between physical activity and frailty.
Risk of bias (arising from measurement of exposure) in the estimated effect of exposure on the outcome	Some concerns	The exposure used in the multivariable analyses was based on a single self-reported assessment of leisure-time physical activity at baseline (1974). Although physical activity was reassessed at follow-up, repeated measurements were not incorporated into the primary adjusted analysis, which may have resulted in exposure misclassification over the 26-year follow-up.
What is the predicted direction of bias arising from measurement of exposure?	Towards null	Exposure misclassification due to a single self-reported baseline assessment would most likely attenuate the true association between physical activity and frailty.
Is the risk of bias (arising from measurement of exposure) sufficiently high, in the context of its likely direction and the magnitude of the estimated exposure effect, to threaten conclusions about whether the exposure has an important effect on the outcome?	No	Although exposure was based on a single self-reported baseline assessment, any resulting misclassification was likely to be largely non-differential and is unlikely to materially affect the study conclusions.

Y = Yes; PY = Probably yes; PN = Probably no; N = No; SY = Strong yes; WY = Weak yes; SN = Strong no; WN = Weak no; NA = Not applicable; NI = No information

*Domain 2, Variant (b): **If Y/PY to C5 and Y/PY to C6** (each individual's exposure level was estimated from measurements made at multiple time points)*

Signalling questions	Response options	Comments
2.1 Does the measured exposure (derived from measurements at multiple time points) well-characterize the exposure metric specified to be of interest in this study? [*This was specified in the answers to D2, D3 and D4*]	Y / PY / WN (no, to a small extent) / SN (no, to a large extent) / NI	
2.2 Was there error in measurement, or misclassification, of the exposure, at each single time point?	SY (yes, probably a substantial amount) / WY (yes, but probably not a substantial amount) / PN / N / NI	
2.3 If SY/WY to 2.2: Could mismeasurement or misclassification of exposure have been differential (i.e. related to the outcome or risk of the outcome)?	NA / SY (yes, to a large extent) / WY (yes, to a small extent) / PN / N / NI	
2.4 If SY/WY to 2.2 and N/PN/WY to 2.3: Is the nature of the (non-differential) measurement error likely to bias the estimated effect of exposure on outcome?	NA / SY (yes, to a large extent) / WY (yes, to a small extent) / PN / N / NI	
Risk of bias (arising from measurement of exposure) in the estimated effect of exposure on the outcome	Low risk / Some concerns / High risk / Very high risk	
What is the predicted direction of bias arising from measurement of exposure?	Towards benefit of (higher) exposure / Towards harm of (higher) exposure / Towards null / Away from null / Insufficient information available	
Is the risk of bias (arising from measurement of exposure) sufficiently high, in the context of its likely direction and the magnitude of the estimated exposure effect, to threaten conclusions about whether the exposure has an important effect on the outcome?	Yes / No / Cannot tell	

Y = Yes; PY = Probably yes; SN = Strong no; WN = Weak no; NA = Not applicable; NI = No information

Domain 2, Variant (c): ***If Y/PY to C5, N/PN to C6 and Y/PY to C7*** *(the analysis was based on splitting participants' follow up time according to exposure status and/or magnitude):*

**Signalling questions**	**Response options**	**Comments**
2.1 Does the measured exposure (including changes over time) well-characterize the exposure metric specified to be of interest in this study? [*This was specified in the answers to D2, D3 and D4*]	Y / PY / WN (no, to a small extent) / SN (no, to a large extent) / NI	
2.2 Was there error in measurement, or misclassification, of the exposure, at each single time point?	SY (yes, probably a substantial amount) / WY (yes, but probably not a substantial amount) / PN / N / NI	
2.3 If SY/WY to 2.2: Could mismeasurement or misclassification of exposure have been differential (i.e. related to the outcome or risk of the outcome)?	NA / SY (yes, to a large extent) / WY (yes, to a small extent) / PN / N / NI	
2.4 If SY/WY to 2.2 and N/PN/WY to 2.3: Is the nature of the (non-differential) measurement error likely to bias the estimated effect of exposure on outcome?	NA / SY (yes, to a large extent) / WY (yes, to a small extent) / PN / N / NI	
Risk of bias (arising from measurement of exposure) in the estimated effect of exposure on the outcome	Low risk / Some concerns / High risk / Very high risk	
What is the predicted direction of bias<br><br>arising from measurement of exposure?	Towards benefit of (higher) exposure / Towards harm of (higher) exposure / Towards null / Away from null / Insufficient information available	
Is the risk of bias (arising from measurement of exposure) sufficiently high, in the context of its likely direction and the magnitude of the estimated exposure effect, to threaten conclusions about whether the exposure has an important effect on the outcome?	Yes / No / Cannot tell	

Y = Yes; PY = Probably yes; SN = Strong no; WN = Weak no; NA = Not applicable; NI = No information

Domain 3: Risk of bias in selection of participants into the study (or into the analysis)

Signalling questions	Response options	Comments
3.1 Did follow-up begin at (or close to) the start of the exposure window for most participants? [*The exposure window is specified in D3*]	Y	Participants were enrolled and exposure was assessed at baseline (1974), before follow-up for frailty began.
3.2 **If N/PN to 3.1**: Is the effect of exposure likely to be constant over the period of follow up analysed?	NA	The association between midlife physical activity and frailty may vary over the long follow-up period.
3.3 Was selection of participants into the study (or into the analysis) based on participant characteristics observed after the start of the exposure window being studied? [*The exposure window is specified in D3*]	Y	The analysis included only participants who survived and attended the 26-year follow-up assessment.
3.4 **If Y/PY to 3.3**: Were these characteristics likely to be influenced by exposure or a cause of exposure?	PY	Midlife physical activity is likely to influence long-term survival and the probability of participating in follow-up.
3.5 **If Y/PY to 3.4**: Were these characteristics likely to be influenced by the outcome or a cause of the outcome?	PY	Frailty and poor health may have reduced the likelihood of attending the follow-up assessment.
3.6 **If N/PN to 3.2 or Y/PY to 3.5**: Is it likely that the analysis corrected for all of the potential selection biases identified in A and B above?	PN	No statistical methods were reported to address potential selection bias due to loss to follow-up or survivor bias.
3.7 **If N/PN to 3.2 or Y/PY to 3.5**: Did sensitivity analyses demonstrate that the likely impact of the potential selection biases identified in A or B above was minimal?	WN	Baseline characteristics of participants and non-participants were compared, but no formal sensitivity analyses for selection bias were reported.
Risk of bias (due to selection of participants into the study) in the estimated effect of exposure on the outcome	Some concerns	Only participants who survived and attended follow-up were included, so some selection bias cannot be excluded
What is the predicted direction of bias<br><br>due to selection of participants into the study?	Towards benefit of (higher) exposure	Healthier and more physically active participants were more likely to survive and attend follow-up, which may have overestimated the protective effect of physical activity
Is the risk of bias (due to selection of participants into the study) sufficiently high, in the context of its likely direction and the magnitude of the estimated exposure effect, to	No	Although survivor bias is possible, it is unlikely to have changed the overall study conclusions

Signalling questions	Response options	Comments
threaten conclusions about whether the exposure has an important effect on the outcome?		

Y = Yes; PY = Probably yes; PN = Probably no; N = No; SN = Strong no; WN = Weak no; NA = Not applicable; NI = No information

Domain 4: Risk of bias due to post-exposure interventions

Signalling questions	Response options	Comments
4.1 Were there post-exposure interventions that were influenced by prior exposure during the follow-up period?	N	No post-exposure interventions influenced by baseline physical activity were reported or considered in the analysis.
4.2 **If Y/PY to 4.1**: Is it likely that the analysis corrected for the effect of post-exposure interventions that were influenced by prior exposure?	NA	Not applicable, as no relevant post-exposure interventions were identified.
Risk of bias (due post-exposure interventions) in the estimated effect of exposure on the outcome	Low risk	No evidence of post-exposure interventions that could have introduced bias into the estimated association between physical activity and frailty.
What is the predicted direction of bias due to confounding?	Insufficient information available	No relevant post-exposure interventions were identified.
Is the risk of bias (due post-exposure interventions) sufficiently high, in the context of its likely direction and the magnitude of the estimated exposure effect, to threaten conclusions about whether the exposure has an important effect on the outcome?	No	Bias due to post-exposure interventions is unlikely to affect the study conclusions.

Y = Yes; PY = Probably yes; PN = Probably no; N = No; NA = Not applicable; NI = No information

Domain 5: Risk of bias due to missing data

Signalling questions	Response options	Comments
5.1 Were complete data on exposure status available for all, or nearly all, participants?	Y	Baseline leisure-time physical activity was available for the study participants included in the analysis.
5.2 Were complete data on the outcome available for all, or nearly all, participants?	PN	Frailty status was unavailable for participants who died or did not attend the 26-year follow-up assessment.
5.3 Were complete data on confounding variables available for all, or nearly all, participants?	PY	Baseline confounding variables were available for most participants included in the multivariable analyses.
5.4 **If N/PN/NI to 5.1, 5.2 or 5.3**: Is the result based on a complete case analysis?	Y	The analyses were based on participants with complete data available at follow-up.
5.5 **If Y/PY/NI**: Was exclusion from the analysis because of missing data (in exposure, confounders or the outcome) likely to be related to the true value of the outcome?	WY	Participants who died or were unable to attend follow-up were more likely to have poorer health and a higher risk of frailty.
5.6 **If N/PN to 5.5**: Were all or most predictors of missingness (in exposure, confounders or the outcome) included in the analysis model?	PN	No evidence was provided that predictors of missingness were incorporated into the statistical models.
5.7 **If N/PN to 5.4**: Was the analysis based on imputing missing values?	N	No imputation methods were reported.
5.8 **If Y/PY to 5.7**: Was imputation performed appropriately?	NA	
5.9 **If N/PN to 5.7**: Was an appropriate alternative method used to correct for bias due to missing data?	N	No alternative methods, such as multiple imputation or inverse probability weighting, were reported.
5.10 **If PN/N/NI to 5.1, 5.2 or 5.3**: Is there evidence that the result was not biased by missing data?	PN	
Risk of bias (due to missing data) in the estimated effect of exposure on the outcome	Some concerns	About 17% of the original cohort was not included in the final analysis, and no methods were used to address missing data.

Signalling questions	Response options	Comments
What is the predicted direction of bias due to missing data?	Towards benefit of (higher) exposure	Participants lost to follow-up may have been frailer and less physically active, which could have overestimated the protective effect of physical activity.
Is the risk of bias (due to missing data) sufficiently high, in the context of its likely direction and the magnitude of the estimated exposure effect, to threaten conclusions about whether the exposure has an important effect on the outcome?	No	Although missing data may have introduced some uncertainty, it is unlikely to have materially affected the overall study conclusions.

Y = Yes; PY = Probably yes; PN = Probably no; N = No; SY = Strong yes; WY = Weak yes; NA = Not applicable; NI = No information

Domain 6: Risk of bias arising from measurement of the outcome

Signalling questions	Response options	Comments
6.1 Could measurement or ascertainment of the outcome have differed between exposure groups or levels of exposure?	N	Frailty was assessed using the same modified Fried phenotype for all participants, regardless of baseline physical activity level.
6.2 Were outcome assessors aware of study participants' exposure history?	PY	Blinding of outcome assessors was not reported. However, frailty was assessed 26 years after baseline exposure measurement.
6.3 **If Y/PY/NI to 6.2**: Could assessment of the outcome have been influenced by knowledge of participants' exposure history?	WN	Although assessor blinding was not reported, it is unlikely that knowledge of baseline physical activity materially influenced frailty assessment
Risk of bias (arising from measurement of outcomes) in the estimated effect of exposure on the outcome	Low risk	The same outcome assessment was applied to all participants, making differential outcome measurement unlikely.
What is the predicted direction of bias arising from measurement of outcomes?	Towards null	Any non-differential outcome misclassification would most likely attenuate the observed association.
Is the risk of bias (arising from measurement of outcomes) sufficiently high, in the context of its likely direction and the magnitude of the estimated exposure effect, to threaten conclusions about whether the exposure has an important effect on the outcome?	No	Any remaining outcome measurement bias is unlikely to have materially affected the study conclusions.

Y = Yes; PY = Probably yes; PN = Probably no; N = No; SY = Strong yes; WY = Weak yes; NA = Not applicable; NI = No information

Domain 7: Risk of bias in selection of the reported result

Signalling questions	Response options	Comments
7.1 Was the result reported in accordance with an available, pre-determined analysis plan?	PN	No pre-specified analysis plan or study protocol was available to verify whether analyses were pre-defined.
7.2 **If N/PN/NI to 7.1**: Is the reported effect estimate likely to be selected, based on desirability of the magnitude (or statistical significance) of the estimated effect of exposure on outcome, from multiple *exposure measurements* within the exposure domain?	N	There is no evidence that the reported effect estimate was selected from multiple exposure measurements based on statistical significance.
7.3 Is the reported effect estimate likely to be selected, based on desirability of the magnitude (or statistical significance) of the estimated effect of exposure on outcome, from multiple *outcome measurements* within the outcome domain?	N	No evidence suggests selective reporting among multiple outcome measurements.
7.4 Is the reported effect estimate likely to be selected, based on desirability of the magnitude (or statistical significance) of the estimated effect of exposure on outcome, from multiple *analyses* of the exposure-outcome relationship?	PN	The study reported several adjusted models and additional analyses, with no indication that only favourable analyses were selected.
7.5 Is the reported effect estimate likely to be selected, based on the basis of desirability of the results (e.g. statistical significance), from different *subgroups*?	N	There is no evidence that the reported results were selected on the basis of statistical significance.
Risk of bias (due to selection of the reported result) in the estimated effect of exposure on the outcome	Low risk	Although no published protocol was available, there is no indication of selective reporting of analyses or results.
What is the predicted direction of bias due to selection of the reported result?	Insufficient information available	No evidence of selective reporting was identified; therefore, the direction of any potential bias cannot be determined.
Is the risk of bias (due to selection of the reported result) sufficiently high, in the context of its likely direction and the magnitude of the estimated exposure effect, to	No	There is no evidence that selection of the reported result materially threatens the study conclusions.

Signalling questions	Response options	Comments
threaten conclusions about whether the exposure has an important effect on the outcome?		

Y = Yes; PY = Probably yes; PN = Probably no; N = No; NA = Not applicable; NI = No information

Overall risk of bias

	Response options	Comments
Overall risk of bias	Some concerns	The study adjusted for most major baseline confounders and used standardized outcome assessment across participants. However, some concerns remain because baseline frailty was not formally assessed, physical activity was self-reported at baseline, and moderate loss to follow-up was handled using complete-case analysis without methods to address missing data. In addition, the cohort consisted exclusively of men of relatively high socioeconomic status, limiting the generalizability of the findings.
What is the predicted direction of bias?	Towards benefit of (higher) exposure	Residual confounding, exposure misclassification and selective loss to follow-up are more likely to overestimate the protective association between higher physical activity and frailty.
Is the overall risk of bias sufficiently high, in the context of its likely direction and the magnitude of the estimated exposure effect, to threaten conclusions about whether the exposure has an important effect on the outcome?	No	Although several sources of bias were identified, they are unlikely to fully explain the observed association. Therefore, the overall conclusions of the study are unlikely to be materially affected..

This work is licensed under a Creative Commons Attribution-NonCommercial-NoDerivatives 4.0 International License.

Wang et al.

For each study result: preliminary considerations

A. Specify the result being assessed for risk of bias

A1. Specify the numerical result being assessed

**Adjusted hazard ratios (95% CIs) from multivariable Cox proportional hazards models estimating the association between long-term physical activity trajectories (persistently active and inclining trajectories versus persistently inactive trajectory) and incident frailty.**

B. Decide whether to proceed with a risk-of-bias assessment

	Response options	Comments
B1. Did the authors make any attempt to control for confounding?	Y	The authors adjusted for major demographic, lifestyle and clinical confounders using multivariable Cox regression.
B2. **If N/PN to B1**: Is there sufficient potential for confounding that an unadjusted result should not be considered further?	NA	NA
B3. Was the method of measuring exposure inappropriate?	PN	Physical activity was assessed repeatedly using a standardized self-reported questionnaire. Although self-report may introduce recall bias, the exposure assessment was appropriate for this cohort study.
B4. Was the method of measuring the outcome inappropriate?	N	Incident frailty was assessed using a validated frailty index based on standardized procedures.

If the answer to any of B2, B3 or B4 is 'Yes' or 'Probably yes', the result should be considered to be at very high risk of bias and no further assessment is required. Otherwise, proceed to section C.

C. Specify the analysis in the current study for which results are being assessed for risk of bias

C1. Specify the outcome to which this result relates.

Incident frailty

C2. Specify the participant group on which this result was based.

Community-dwelling adults aged ≥50 years from the English Longitudinal Study of Ageing (ELSA) who were non-frail at baseline (wave 4).

C3 to C8: Describe the exposure measurement(s) used to produce this result.

C3. What is the exposure being measured and how was it measured or assessed?	Long-term physical activity participation trajectories assessed using repeated self-reported physical activity questionnaires across waves 1–4 and classified using group-based trajectory modelling (GBTM).
C4. Was exposure analysed as a quantitative (rather than a categorical) variable?	N
C5. Did repeated measurements of exposure over time (for each participant) contribute to the analysis that produced this result?	Y
C6. **If Y/PY to C5**, was a single estimate of each participant's exposure level derived from the repeated measurements of exposure over time?	Y
C7. **If N/PN to C6**, was the analysis based on splitting participants' follow up time according to exposure status and/or magnitude?	NA
C8. **If Y/PY to C7**, were changes in exposure status and/or magnitude likely to be related to factors that are predictive of the outcome?	NA
C9. **If N/PN to C7**, how were repeat measurements used?	

Y = Yes; PY = Probably yes; PN = Probably no; N = No; NA = Not applicable

C10. Specify the relationship analysed to produce this result. For example, this may be a quadratic relationship of cumulative exposure with the log odds of the outcome, or a risk ratio for the outcome comparing exposed with unexposed individuals.

Association between long-term physical activity trajectory groups and incident frailty estimated using multivariable Cox proportional hazards regression.

D: Specify the causal effect of exposure being estimated by this result

D1. Specify the population of interest	Describe eligible participants (to whom the causal effect applies). These may be different from the study participants on whom the result was based (specified in C2). Such differences may give rise to selection biases.	Community-dwelling adults aged ≥50 years who were non-frail at baseline.
Specification of the exposure metric of interest		
D2. Specify the exposure	This is the factor whose causal effect on the outcome of interest is the subject of the study result being assessed. It may be thought of as the 'true' exposure of interest. It is distinct from the method with which exposure was measured.	Long-term physical activity participation trajectories.
D3. Specify the exposure window	The exposure window of interest is the exposure period for which the result being assessed estimates the effect of exposure on the outcome. Specification of the exposure window is judged by the ROBINS-E user, who should aim to define a window that is both meaningful in answering the review question and broadly in line with when the study measured exposure. Specification should include both the time of onset and period of exposure. For example, it may be lifetime exposure (from birth or from conception), during ages 50-55, the period from first employment in a particular occupation, time from birth to age 10, or during pregnancy.<br><br>The specified exposure window is used to determine whether exposure data adequately reflect exposure during the window. Exposure before the start of the exposure window is addressed during the assessment of risk of bias due to confounding	Physical activity participation over the 6-year period from ELSA wave 1 (2002–2003) to wave 4 (2008–2009).
D4. Specify how exposure over time	This may, for example, be ever/never exposed, cumulative exposure, average exposure, or peak exposure during the exposure period, for each participant.	Exposure was summarized as group-based physical activity trajectories (persistently low, increasing,

should be summarized	Alternatively, there may be only a single exposure event, or the exposure may be time invariant (such as a genetic variant or family history).	declining, persistently moderate, and persistently high) derived from repeated measurements over six years.

E. Evaluation of confounding factors

Complete a row for each important confounding factor listed in advance (subsection (i)). In addition, consider any further confounding factors that are either relevant to the setting of this particular study or which the study authors identified as potentially important (subsection (ii)).

"Important" confounding factors are those for which, in the context of this study, adjustment is expected to lead to an important change in the estimated effect of the exposure.

(i) Important confounding factors listed in advance						
Confounding factor	Measured variable(s) for this factor, if any	Was this variable (or were these variables) controlled for in the analysis?<br>(Y / N)	If this confounding factor was controlled for, was it measured validly and reliably by this variable (or these variables)?*<br>(NA / Y / PY / PN / N / NI)	If this confounding factor was not controlled for, is there evidence that controlling for it was unnecessary?**<br>(NA / Y / PY / PN / N)	Is failure to adjust for this confounding factor expected to bias the effect estimate towards benefit or harm of (higher) exposure?***<br>(Benefit of (higher) exposure / Harm of (higher) exposure / Insufficient information available)	Comments
Age	Y	Y	Y	NA	Towards benefit of higher exposure	Age was measured and included as an adjustment variable in the multivariable models. Older age is associated with lower physical activity levels and a higher risk of frailty.

Sex	Y	Y	Y	NA	Towards benefit of higher exposure	Sex was accurately recorded and adjusted for in the multivariable models. Sex differences are associated with both physical activity patterns and frailty risk.
**Baseline frailty/physical function** (grip strength, gait speed, chair rise, frailty index)	**Baseline frailty/physical function**	Y	Y	NA	Towards benefit of higher exposure	Participants with prevalent frailty were excluded at baseline. Baseline physical function was measured and adjusted, whereas baseline frailty status was not included as an adjustment variable.
BMI		Y	Y	NA	Towards benefit of higher exposure	BMI was included as an adjustment variable in multivariable models.
Lifestyle factors	Smoking/alcohol/vigorous exercise	Y	Y	NA	Towards benefit of higher exposure	Smoking, alcohol consumption and lifestyle behaviours were adjusted for in multivariable analyses.
**Comorbidities** (hypertension, diabetes, stroke, CVD, chronic lung disease, cancer, depressive symptoms, functional limitations)	Y	Y	Y	NA	Towards benefit of higher exposure	Major chronic diseases (hypertension, diabetes, stroke, CVD, COPD, cancer) were adjusted for.

Socioeconomic status	Educational background	Y	N	NA	NA	Educational background was included as an indicator of socioeconomic status.

(ii) Additional confounding factors relevant to the setting of this particular study, or identified by study authors and considered to be important, or which were identified since the protocol was written						
Confounding factor	Measured variable(s) for this factor, if any	Was this variable (or were these variables) controlled for in the analysis?<br>(Y / N)	If this confounding factor was controlled for, was it measured validly and reliably by this variable (or these variables)?*<br>(NA / Y / PY / PN / N / NI)	If this confounding factor was not controlled for, is there evidence that controlling for it was unnecessary?**<br>(NA / Y / PY / PN / N)	Is failure to adjust for this confounding factor expected to bias the effect estimate towards benefit or harm of (higher) exposure?*** (Benefit of (higher) exposure / Harm of (higher) exposure / Insufficient information available)	Comments
Cohabitation status	Living alone	Y	PY	NA	Towards benefit of higher exposure	Living alone (cohabitation status) was included as a social confounder in adjusted analyses.
Inflammatory biomarkers (e.g., IL-6)	N	N	PN	NA	Insufficient information available	Inflammatory biomarkers (e.g., IL-6) were not measured or adjusted for. The authors acknowledged these as potential sources of residual confounding.

* "Validity" refers to whether the confounding variable or variables accurately measure the confounding factor, while "reliability" refers to the precision of the measurement (more measurement error means less reliability).

** In the context of a particular study, variables need not be included in the analysis: (a) if they are measured validly and reliably and are not associated with the outcome, conditional on exposure (noting that lack of a statistically significant association is not evidence of a lack of association); (b) if they are measured validly and reliably and are not associated with exposure; (c) if they are measured validly and reliably and adjustment makes no or minimal difference to the estimated effect of the primary parameter; (d) because the confounder was addressed in the study design, for example by restricting to individuals with the

same value of the confounder; (e) because a negative control demonstrates that there was unlikely to have been confounding due to this variable or that uncontrolled confounding was likely to be minimal; or (f) because external evidence suggests that controlling for the variable is not necessary in the context of the study being assessed..

For each study: risk of bias assessment

Domain 1: Risk of bias due to confounding

*Domain 1, Variant (a): **If N/PN to C5 or Y/PY to C6 or N/PN to C7** (only baseline confounding needs to be addressed)*

Signalling questions	Response options	Comments
1.1 Did the authors control for all the important confounding factors for which this was necessary?	PY	Demographic, lifestyle and clinical confounders were adjusted for using multivariable Cox regression. However, residual confounding from unmeasured factors (e.g., inflammatory biomarkers) cannot be excluded.
1.2 **If Y/PY/WN to 1.1**: Were confounding factors that were controlled for (and for which control was necessary) measured validly and reliably by the variables available in this study?	PY	Most confounders were measured using standard methods, although physical activity was self-reported
1.3 **If Y/PY/WN to 1.1**: Did the authors control for any variables after the start of the exposure period being studied that could have been affected by the exposure?	PN	No post-exposure variables appear to have been adjusted for.
1.4 Did the use of negative controls, or other considerations, suggest serious uncontrolled confounding?	PN	No evidence of serious uncontrolled confounding was reported.
Risk of bias (due to confounding) in the estimated effect of exposure on the outcome	Some concerns	Most important confounders were adjusted for, but some residual confounding cannot be excluded.
What is the predicted direction of bias due to confounding?	(Towards benefit of (higher) exposure	Residual confounding may make higher physical activity appear more protective than it really is.
Is the risk of bias (due to confounding) sufficiently high, in the context of its likely direction and the magnitude of the estimated exposure effect, to threaten conclusions about whether the exposure has an important effect on the outcome?	No	Any remaining confounding is unlikely to materially affect the estimated association.

Y = Yes; PY = Probably yes; PN = Probably no; N = No; SY = Strong yes; WY = Weak yes; SN = Strong no; WN = Weak no; NA = Not applicable; NI = No information

Domain 1, variant (b): ***If Y/PY to C7 and Y/PY to C8*** *(the analysis was based on splitting participants' follow up time according to exposure status and/or magnitude and changes in exposure status and/or magnitude likely to be related to factors that are predictive of the outcome, so both baseline and time-varying confounding need to be addressed)*

**Signalling questions**	**Response options**	**Comments**
1.1 Did the authors use an analysis method that was appropriate to control for time-varying as well as baseline confounding?	Y / PY / PN / N / NI	
1.2 **If Y/PY to 1.1**: Did the authors control for all the important baseline and time-varying confounding factors for which this was necessary?	NA / Y / PY / WN (no, but uncontrolled confounding was probably not substantial) / SN (no, and uncontrolled confounding was probably substantial) / NI	
1.3 **If Y/PY/WN to 1.2**: Were confounding factors that were controlled for (and for which control was necessary) measured validly and reliably by the variables available in this study?	NA / Y / WN (no, but the extent of measurement error in confounding factors was probably not substantial) / SN (no, and the extent of measurement error in confounding factors was probably substantial) / NI	
1.4 **If N/PN/NI to 1.1**: Did the authors control for time-varying factors or other variables measured after the start of the exposure window being studied?	NA / Y / PY / PN / N / NI	
1.5 Did the use of negative controls, or other considerations, suggest uncontrolled confounding?	Y / PY / PN / N	
Risk of bias (due to confounding) in the estimated effect of exposure on the outcome	Low risk / Some concerns / High risk / Very high risk	

Signalling questions	Response options	Comments
What is the predicted direction of bias due to confounding?	Towards benefit of (higher) exposure / Towards harm of (higher) exposure / Towards null / Away from null / Insufficient information available	
Is the risk of bias (due to confounding) sufficiently high, in the context of its likely direction and the magnitude of the estimated exposure effect, to threaten conclusions about whether the exposure has an important effect on the outcome?	Yes / No / Cannot tell	

Y = Yes; PY = Probably yes; PN = Probably no; N = No; SY = Strong yes; WY = Weak yes; SN = Strong no; WN = Weak no; NA = Not applicable; NI = No information

Domain 2: Risk of bias arising from measurement of the exposure

*Domain 2, Variant (a): **If N/PN to C5** (exposure was measured at a single point in time)*

Signalling questions	Response options	Comments
Mismeasurement or misclassification of the exposure.		
2.1 Does the measured exposure well-characterize the exposure metric specified to be of interest in this study? [*This was specified in the answers to D2, D3 and D4*]		
2.2 Was the exposure likely to be measured with error, or misclassified?		
Bias in the estimated effect of exposure arising from mismeasurement or misclassification of the exposure		
2.3 If SY/WY to 2.2: Could mismeasurement or misclassification of exposure have been differential (i.e. related to the outcome or risk of the outcome)?		
2.4 If SY/WY to 2.2 and N/PN/WY to 2.3: Is non-differential measurement error likely to bias the estimated effect of exposure on outcome?		
Risk of bias (arising from measurement of exposure) in the estimated effect of exposure on the outcome		
What is the predicted direction of bias arising from measurement of exposure?		
Is the risk of bias (arising from measurement of exposure) sufficiently high, in the context of its likely direction and the magnitude of the estimated exposure effect, to threaten conclusions about whether the exposure has an important effect on the outcome?		

Y = Yes; PY = Probably yes; PN = Probably no; N = No; SY = Strong yes; WY = Weak yes; SN = Strong no; WN = Weak no; NA = Not applicable; NI = No information

*Domain 2, Variant (b): **If Y/PY to C5 and Y/PY to C6** (each individual's exposure level was estimated from measurements made at multiple time points)*

Signalling questions	Response options	Comments
2.1 Does the measured exposure (derived from measurements at multiple time points) well-characterize the exposure metric specified to be of interest in this study? [*This was specified in the answers to D2, D3 and D4*]	Y	Repeated self-reported assessments improved the characterization of long-term physical activity trajectories, although some measurement error may remain.
2.2 Was there error in measurement, or misclassification, of the exposure, at each single time point?	WY	Physical activity was self-reported, so some recall and reporting error is possible
2.3 If SY/WY to 2.2: Could mismeasurement or misclassification of exposure have been differential (i.e. related to the outcome or risk of the outcome)?	PN	Physical activity was assessed before frailty occurred, making differential exposure misclassification unlikely.
2.4 If SY/WY to 2.2 and N/PN/WY to 2.3: Is the nature of the (non-differential) measurement error likely to bias the estimated effect of exposure on outcome?	WN	Any remaining measurement error is likely non-differential and would attenuate the association.
Risk of bias (arising from measurement of exposure) in the estimated effect of exposure on the outcome	Some concerns	Exposure assessment relied on self-report, but repeated measurements substantially improved exposure characterization over time.
What is the predicted direction of bias arising from measurement of exposure?	Towards null	Such non-differential exposure misclassification would be expected to dilute the association towards the null.
Is the risk of bias (arising from measurement of exposure) sufficiently high, in the context of its likely direction and the magnitude of the estimated exposure effect, to threaten conclusions about whether the exposure has an important effect on the outcome?	No	Remaining measurement error is unlikely to be sufficient to threaten the study conclusions because repeated assessments reduced exposure misclassification.

Y = Yes; PY = Probably yes; SN = Strong no; WN = Weak no; NA = Not applicable; NI = No information

*Domain 2, Variant (c): **If Y/PY to C5, N/PN to C6 and Y/PY to C7** (the analysis was based on splitting participants' follow up time according to exposure status and/or magnitude):*

Signalling questions	Response options	Comments
2.1 Does the measured exposure (including changes over time) well-characterize the exposure metric specified to be of interest in this study? [*This was specified in the answers to D2, D3 and D4*]	Y / PY / WN (no, to a small extent) / SN (no, to a large extent) / NI	
2.2 Was there error in measurement, or misclassification, of the exposure, at each single time point?	SY (yes, probably a substantial amount) / WY (yes, but probably not a substantial amount) / PN / N / NI	
2.3 If SY/WY to 2.2: Could mismeasurement or misclassification of exposure have been differential (i.e. related to the outcome or risk of the outcome)?	NA / SY (yes, to a large extent) / WY (yes, to a small extent) / PN / N / NI	
2.4 If SY/WY to 2.2 and N/PN/WY to 2.3: Is the nature of the (non-differential) measurement error likely to bias the estimated effect of exposure on outcome?	NA / SY (yes, to a large extent) / WY (yes, to a small extent) / PN / N / NI	
Risk of bias (arising from measurement of exposure) in the estimated effect of exposure on the outcome	Low risk / Some concerns / High risk / Very high risk	
What is the predicted direction of bias arising from measurement of exposure?	Towards benefit of (higher) exposure / Towards harm of (higher) exposure / Towards null / Away from null / Insufficient information available	
Is the risk of bias (arising from measurement of exposure) sufficiently high, in the context of its likely direction and the magnitude of the estimated exposure effect, to threaten conclusions about whether the exposure has an important effect on the outcome?	Yes / No / Cannot tell	

Y = Yes; PY = Probably yes; SN = Strong no; WN = Weak no; NA = Not applicable; NI = No information

Domain 3: Risk of bias in selection of participants into the study (or into the analysis)

Signalling questions	Response options	Comments
3.1 Did follow-up begin at (or close to) the start of the exposure window for most participants? [*The exposure window is specified in D3*]	Y	Follow-up began at the start of the exposure assessment.
3.2 **If N/PN to 3.1**: Is the effect of exposure likely to be constant over the period of follow up analysed?	NA	
3.3 Was selection of participants into the study (or into the analysis) based on participant characteristics observed after the start of the exposure window being studied? [*The exposure window is specified in D3*]	PY	Participants with missing exposure or outcome data and those lost to follow-up were excluded from the analysis. The authors acknowledged that excluded individuals were older and had a higher prevalence of chronic diseases and living alone, indicating potential attrition-related selection bias.
3.4 **If Y/PY to 3.3**: Were these characteristics likely to be influenced by exposure or a cause of exposure?	PY	Those lost to follow-up were older and less healthy.
3.5 **If Y/PY to 3.4**: Were these characteristics likely to be influenced by the outcome or a cause of the outcome?	PN	Selection occurred before the outcome assessment.
3.6 **If N/PN to 3.2 or Y/PY to 3.5**: Is it likely that the analysis corrected for all of the potential selection biases identified in A and B above?	PN	No methods were used to address selection bias
3.7 **If N/PN to 3.2 or Y/PY to 3.5**: Did sensitivity analyses demonstrate that the likely impact of the potential selection biases identified in A or B above was minimal?	WN	Sensitivity analyses did not specifically assess selection bias
Risk of bias (due to selection of participants into the study) in the estimated effect of exposure on the outcome	Some concerns	Participants lost to follow-up were excluded, but the impact is unlikely to be substantial
What is the predicted direction of bias<br><br>due to selection of participants into the study?	Towards null	Loss to follow-up of older, less healthy participants may have weakened the observed association.
Is the risk of bias (due to selection of participants into the study) sufficiently high, in the context of its likely direction and the magnitude of the estimated exposure effect, to	No	The potential selection bias is unlikely to have a meaningful impact on the study findings

Signalling questions	Response options	Comments
threaten conclusions about whether the exposure has an important effect on the outcome?		

Y = Yes; PY = Probably yes; PN = Probably no; N = No; SN = Strong no; WN = Weak no; NA = Not applicable; NI = No information

Domain 4: Risk of bias due to post-exposure interventions

Signalling questions	Response options	Comments
4.1 Were there post-exposure interventions that were influenced by prior exposure during the follow-up period?	N	No post-exposure interventions influenced by prior physical activity during follow-up were reported.
4.2 **If Y/PY to 4.1**: Is it likely that the analysis corrected for the effect of post-exposure interventions that were influenced by prior exposure?	NA	Not applicable, as no post-exposure interventions were identified.
Risk of bias (due post-exposure interventions) in the estimated effect of exposure on the outcome	Low risk	No evidence that post-exposure interventions influenced the association between ph activity trajectories and incident frailty.
What is the predicted direction of bias due to confounding?	NA	*Not applicable.*
Is the risk of bias (due post-exposure interventions) sufficiently high, in the context of its likely direction and the magnitude of the estimated exposure effect, to threaten conclusions about whether the exposure has an important effect on the outcome?	No	No post-exposure interventions were identified; therefore, this domain is unlikely to threaten the validity of the estimated association.

Y = Yes; PY = Probably yes; PN = Probably no; N = No; NA = Not applicable; NI = No information

Domain 5: Risk of bias due to missing data

Signalling questions	Response options	Comments
5.1 Were complete data on exposure status available for all, or nearly all, participants?	PY	Physical activity trajectories were derived from repeated assessments, but some participants were excluded because of missing follow-up data.
5.2 Were complete data on the outcome available for all, or nearly all, participants?	PY	Incident frailty was assessed prospectively; however, participants lost to follow-up did not have outcome data available
5.3 Were complete data on confounding variables available for all, or nearly all, participants?	PY	Confounders were comprehensively assessed, although complete availability for all participants was not explicitly reported.
5.4 **If N/PN/NI to 5.1, 5.2 or 5.3**: Is the result based on a complete case analysis?	Y	Participants with missing data and those lost to follow-up were excluded from the final analysis (complete-case analysis).
5.5 **If Y/PY/NI**: Was exclusion from the analysis because of missing data (in exposure, confounders or the outcome) likely to be related to the true value of the outcome?	WY	Participants lost to follow-up may have differed in frailty risk, making outcor missingness plausible.
5.6 **If N/PN to 5.5**: Were all or most predictors of missingness (in exposure, confounders or the outcome) included in the analysis model?	PN	No inverse probability weighting or other modelling of missingness was repo
5.7 **If N/PN to 5.4**: Was the analysis based on imputing missing values?	NA	
5.8 **If Y/PY to 5.7**: Was imputation performed appropriately?	NA	
5.9 **If N/PN to 5.7**: Was an appropriate alternative method used to correct for bias due to missing data?	NA	
5.10 **If PN/N/NI to 5.1, 5.2 or 5.3**: Is there evidence that the result was not biased by missing data?	NA	
Risk of bias (due to missing data) in the estimated effect of exposure on the outcome	Some concerns	Missing data were handled using complete-case analysis without methods t potential bias from loss to follow-up.

Signalling questions	Response options	Comments
What is the predicted direction of bias due to missing data?	Towards null	Loss to follow-up is more likely to attenuate than exaggerate the observed association.
Is the risk of bias (due to missing data) sufficiently high, in the context of its likely direction and the magnitude of the estimated exposure effect, to threaten conclusions about whether the exposure has an important effect on the outcome?	No	Although missing data may have introduced some bias, its magnitude is unlikely to threaten the overall study conclusions.

Y = Yes; PY = Probably yes; PN = Probably no; N = No; SY = Strong yes; WY = Weak yes; NA = Not applicable; NI = No information

Domain 6: Risk of bias arising from measurement of the outcome

Signalling questions	Response options	Comments
6.1 Could measurement or ascertainment of the outcome have differed between exposure groups or levels of exposure?	PN	Frailty was assessed using the same standardized Frailty Index for all participants, regardless of physical activity group.
6.2 Were outcome assessors aware of study participants' exposure history?	NI	It was not reported whether outcome assessors were aware of participants' trajectories.
6.3 **If Y/PY/NI to 6.2**: Could assessment of the outcome have been influenced by knowledge of participants' exposure history?	NA	
Risk of bias (arising from measurement of outcomes) in the estimated effect of exposure on the outcome	Low risk	The outcome was measured using the same standardized method for all participants, making differential outcome measurement unlikely.
What is the predicted direction of bias arising from measurement of outcomes?	Insufficient information available	There is no evidence suggesting a specific direction of bias due to outcome measurement.
Is the risk of bias (arising from measurement of outcomes) sufficiently high, in the context of its likely direction and the magnitude of the estimated exposure effect, to threaten conclusions about whether the exposure has an important effect on the outcome?	No	Outcome measurement is unlikely to have affected the study conclusions.

Y = Yes; PY = Probably yes; PN = Probably no; N = No; SY = Strong yes; WY = Weak yes; NA = Not applicable; NI = No information

Domain 7: Risk of bias in selection of the reported result

Signalling questions	Response options	Comments
7.1 Was the result reported in accordance with an available, pre-determined analysis plan?	NI	No published protocol or statistical analysis plan was available to verify whether the reported analyses were pre-specified.
7.2 **If N/PN/NI to 7.1**: Is the reported effect estimate likely to be selected, based on desirability of the magnitude (or statistical significance) of the estimated effect of exposure on outcome, from multiple *exposure measurements* within the exposure domain?	NA	
7.3 Is the reported effect estimate likely to be selected, based on desirability of the magnitude (or statistical significance) of the estimated effect of exposure on outcome, from multiple *outcome measurements* within the outcome domain?	PN	There was no evidence that results were selectively reported from multiple outcome measurements
7.4 Is the reported effect estimate likely to be selected, based on desirability of the magnitude (or statistical significance) of the estimated effect of exposure on outcome, from multiple *analyses* of the exposure-outcome relationship?	PN	No evidence of selective reporting from multiple analyses of the exposure–outcome relationship was identified.
7.5 Is the reported effect estimate likely to be selected, based on the basis of desirability of the results (e.g. statistical significance), from different *subgroups*?	PN	Subgroup analyses were reported transparently and there was no indication that results were selected based on statistical significance.
Risk of bias (due to selection of the reported result) in the estimated effect of exposure on the outcome	Low risk	No evidence of selective reporting was identified. Although a pre-specified analysis plan was not available, the reported analyses were consistent with the study objectives and methods.
What is the predicted direction of bias due to selection of the reported result?	Insufficient information available	There is no evidence to suggest a specific direction of bias due to selective reporting.
Is the risk of bias (due to selection of the reported result) sufficiently high, in the context of its likely direction and the magnitude of the estimated exposure effect, to	No	Selective reporting is unlikely to have affected the study conclusions.

Signalling questions	Response options	Comments
threaten conclusions about whether the exposure has an important effect on the outcome?		

Y = Yes; PY = Probably yes; PN = Probably no; N = No; NA = Not applicable; NI = No information

Overall risk of bias

	Response options	Comments
Overall risk of bias	Some concerns	Some concerns remain because of potential residual confounding, self-reported physical activity, and missing data.
What is the predicted direction of bias?	Towards benefit of (higher) exposure	The identified sources of bias are more likely to overestimate the protective effect of higher physical activity on frailty.
Is the overall risk of bias sufficiently high, in the context of its likely direction and the magnitude of the estimated exposure effect, to threaten conclusions about whether the exposure has an important effect on the outcome?	No	The identified sources of bias are unlikely to be large enough to change the overall study conclusions.

This work is licensed under a Creative Commons Attribution-NonCommercial-NoDerivatives 4.0 International License.

Borda et al.

For each study result: preliminary considerations

A. Specify the result being assessed for risk of bias

A1. Specify the numerical result being assessed

Adjusted odds ratio (OR) for incident frailty associated with physical activity versus no physical activity: OR 0.7 (95% CI 0.7–0.8).

B. Decide whether to proceed with a risk-of-bias assessment

	Response options	Comments
B1. Did the authors make any attempt to control for confounding?	y	Adjusted for age, sex, marital status, financial status, education and physician visits.
B2. **If N/PN to B1**: Is there sufficient potential for confounding that an unadjusted result should not be considered further?	y	Physical activity and frailty share common causes. The adjusted estimate is the appropriate result.
B3. Was the method of measuring exposure inappropriate?	PN	Physical activity was self-reported using a standardized questionnaire. Although based on a single baseline assessment and a simple question, the measurement was not inappropriate for this cohort study
B4. Was the method of measuring the outcome inappropriate?	PN	Incident frailty was assessed using a validated 39-item Frailty Index derived from standardized questionnaire data.

If the answer to any of B2, B3 or B4 is 'Yes' or 'Probably yes', the result should be considered to be at very high risk of bias and no further assessment is required. Otherwise, proceed to section C.

C. Specify the analysis in the current study for which results are being assessed for risk of bias

C1. Specify the outcome to which this result relates.

Incident frailty over the 3-year follow-up, defined using a validated 39-item Frailty Index.

C2. Specify the participant group on which this result was based.

Community-dwelling Mexican adults aged ≥50 years who were non-frail at baseline (2012) and followed until 2015.

C3 to C8: Describe the exposure measurement(s) used to produce this result.

C3. What is the exposure being measured and how was it measured or assessed?	Physical activity was assessed at baseline by self-report using a standardized questionnaire asking whether participants had exercised or performed hard physical work at least three times per week during the previous two years.
C4. Was exposure analysed as a quantitative (rather than a categorical) variable?	N
C5. Did repeated measurements of exposure over time (for each participant) contribute to the analysis that produced this result?	N
C6. **If Y/PY to C5**, was a single estimate of each participant's exposure level derived from the repeated measurements of exposure over time?	NA
C7. **If N/PN to C6**, was the analysis based on splitting participants' follow up time according to exposure status and/or magnitude?	NA
C8. **If Y/PY to C7**, were changes in exposure status and/or magnitude likely to be related to factors that are predictive of the outcome?	NA
C9. **If N/PN to C7**, how were repeat measurements used?	

Y = Yes; PY = Probably yes; PN = Probably no; N = No; NA = Not applicable

C10. Specify the relationship analysed to produce this result. For example, this may be a quadratic relationship of cumulative exposure with the log odds of the outcome, or a risk ratio for the outcome comparing exposed with unexposed individuals.

The analysis estimated the association between baseline physical activity (yes versus no) and the odds of developing incident frailty over the 3-year follow-up using multivariable logistic regression.

D: Specify the causal effect of exposure being estimated by this result

D1. Specify the population of interest	Describe eligible participants (to whom the causal effect applies). These may be different from the study participants on whom the result was based (specified in C2). Such differences may give rise to selection biases.	Community-dwelling Mexican adults aged ≥50 years who were non-frail at baseline and were followed for incident frailty over 3 years.
Specification of the exposure metric of interest		
D2. Specify the exposure	This is the factor whose causal effect on the outcome of interest is the subject of the study result being assessed. It may be thought of as the 'true' exposure of interest. It is distinct from the method with which exposure was measured.	Regular physical activity, defined as exercising or performing hard physical work at least three times per week during the previous two years.
D3. Specify the exposure window	The exposure window of interest is the exposure period for which the result being assessed estimates the effect of exposure on the outcome. Specification of the exposure window is judged by the ROBINS-E user, who should aim to define a window that is both meaningful in answering the review question and broadly in line with when the study measured exposure. Specification should include both the time of onset and period of exposure. For example, it may be lifetime exposure (from birth or from conception), during ages 50-55, the period from first employment in a particular occupation, time from birth to age 10, or during pregnancy.<br><br>The specified exposure window is used to determine whether exposure data adequately reflect exposure during the window. Exposure before the start of the exposure window is addressed during the assessment of risk of bias due to confounding	Physical activity was reported for the 2 years before baseline. Frailty was assessed over the following 3 years.
D4. Specify how exposure over time	This may, for example, be ever/never exposed, cumulative exposure, average exposure, or peak exposure during the exposure	Exposure was treated as a single baseline measure (ever/never physically active according to the predefined criterion) and was assumed to

should be summarized	period, for each participant. Alternatively, there may be only a single exposure event, or the exposure may be time invariant (such as a genetic variant or family history).	represent participants' habitual physical activity during the exposure window.

E. Evaluation of confounding factors

Complete a row for each important confounding factor listed in advance (subsection (i)). In addition, consider any further confounding factors that are either relevant to the setting of this particular study or which the study authors identified as potentially important (subsection (ii)).

"Important" confounding factors are those for which, in the context of this study, adjustment is expected to lead to an important change in the estimated effect of the exposure.

(i) Important confounding factors listed in advance						
Confounding factor	Measured variable(s) for this factor, if any	Was this variable (or were these variables) controlled for in the analysis?<br>(Y / N)	If this confounding factor was controlled for, was it measured validly and reliably by this variable (or these variables)?*<br>(NA / Y / PY / PN / N / NI)	If this confounding factor was not controlled for, is there evidence that controlling for it was unnecessary?**<br>(NA / Y / PY / PN / N)	Is failure to adjust for this confounding factor expected to bias the effect estimate towards benefit or harm of (higher) exposure?***<br>(Benefit of (higher) exposure / Harm of (higher) exposure / Insufficient information available)	Comments
Age	years	Y	Y	NA	NA	Adjusted in multivariable logistic regression.
Sex	Male/femal e	Y	Y	NA	NA	Included in the adjusted model
BMI/adiposity	Body mass index	N	NA	PN	Towards benefit of higher exposure	Body composition was not measured or adjusted despite being associated with both physical activity and frailty risk.
Baseline health/comorbid ities	Chronic diseases	N	PY	PN	Towards benefit of higher exposure	Chronic diseases contributed to the frailty index but were not comprehensively

						adjusted as baseline confounders in the physical activity analysis.
Baseline frailty / functional status	Baseline frailty	N	PY	PN	Towards benefit of higher exposure	Participants with prevalent frailty were excluded, but baseline frailty components or subclinical functional deficits were not adjusted for and may have influenced both physical activity and subsequent frailty.
Lifestyle factors	Smoking, alcohol intake,diet	N	NA	Y	NA	Other lifestyle behaviours were not adjusted separately because they were included in the healthy behaviour score.
Socioeconomic status	education, financial status, marital status	Y	PY	NA	NA	Education, marital status and financial status were included as covariates, although SES may not have been fully captured.

(ii) Additional confounding factors relevant to the setting of this particular study, or identified by study authors and considered to be important, or which were identified since the protocol was written						
Confounding factor	Measured variable(s) for this factor, if any	Was this variable (or were these	If this confounding factor was controlled for, was it measured validly and	If this confounding factor was not controlled for, is there	Is failure to adjust for this confounding factor expected to bias the effect estimate towards benefit or harm of	Comments

		variables) controlled for in the analysis?<br>(Y / N)	reliably by this variable (or these variables)?*<br>(NA / Y / PY / PN / N / NI)	evidence that controlling for it was unnecessary?**<br>(NA / Y / PY / PN / N)	(higher) exposure?*** (Benefit of (higher) exposure / Harm of (higher) exposure / Insufficient information available)	
Healthcare utilization/aces s	Physician visits during previous 2 years	Y	PY	PN	Towards benefit of higher exposure	Healthcare utilization (physician visits during the previous two years) was included in the adjusted models because access to healthcare may influence both healthy behaviours and subsequent frailty.

* "Validity" refers to whether the confounding variable or variables accurately measure the confounding factor, while "reliability" refers to the precision of the measurement (more measurement error means less reliability).

** In the context of a particular study, variables need not be included in the analysis: (a) if they are measured validly and reliably and are not associated with the outcome, conditional on exposure (noting that lack of a statistically significant association is not evidence of a lack of association); (b) if they are measured validly and reliably and are not associated with exposure; (c) if they are measured validly and reliably and adjustment makes no or minimal difference to the estimated effect of the primary parameter; (d) because the confounder was addressed in the study design, for example by restricting to individuals with the same value of the confounder; (e) because a negative control demonstrates that there was unlikely to have been confounding due to this variable or that uncontrolled confounding was likely to be minimal; or (f) because external evidence suggests that controlling for the variable is not necessary in the context of the study being assessed..

For each study: risk of bias assessment

Domain 1: Risk of bias due to confounding

Domain 1, Variant (a): ***If N/PN to C5 or Y/PY to C6 or N/PN to C7*** *(only baseline confounding needs to be addressed)*

Signalling questions	Response options	Comments
1.1 Did the authors control for all the important confounding factors for which this was necessary?	SN	Adjusted for age, sex, marital status, education, financial status and physician visits. Baseline functional status and overall health were not included
1.2 **If Y/PY/WN to 1.1**: Were confounding factors that were controlled for (and for which control was necessary) measured validly and reliably by the variables available in this study?	PY	Most confounders were collected by questionnaire. Several variables were self-reported
1.3 **If Y/PY/WN to 1.1**: Did the authors control for any variables after the start of the exposure period being studied that could have been affected by the exposure?	N	All covariates were measured at baseline. No post-exposure variables were adjusted for.
1.4 Did the use of negative controls, or other considerations, suggest serious uncontrolled confounding?	PN	No negative controls were reported.
Risk of bias (due to confounding) in the estimated effect of exposure on the outcome	High risk	Baseline functional status and overall health were not fully accounted for.
What is the predicted direction of bias due to confounding?	Towards benefit of (higher) exposure	Uncontrolled baseline health and functional status may have overestimated the protective effect of physical activity on incident frailty.
Is the risk of bias (due to confounding) sufficiently high, in the context of its likely direction and the magnitude of the estimated exposure effect, to threaten conclusions about whether the exposure has an important effect on the outcome?	Yes	Key health-related confounders were not reported, so residual confounding is likely.

Y = Yes; PY = Probably yes; PN = Probably no; N = No; SY = Strong yes; WY = Weak yes; SN = Strong no; WN = Weak no; NA = Not applicable; NI = No information

*Domain 1, variant (b): **If Y/PY to C7 and Y/PY to C8** (the analysis was based on splitting participants' follow up time according to exposure status and/or magnitude and changes in exposure status and/or magnitude likely to be related to factors that are predictive of the outcome, so both baseline and time-varying confounding need to be addressed)*

**Signalling questions**	**Response options**	**Comments**
1.1 Did the authors use an analysis method that was appropriate to control for time-varying as well as baseline confounding?	Y / PY / PN / N / NI	
1.2 **If Y/PY to 1.1**: Did the authors control for all the important baseline and time-varying confounding factors for which this was necessary?	NA / Y / PY / WN (no, but uncontrolled confounding was probably not substantial) / SN (no, and uncontrolled confounding was probably substantial) / NI	
1.3 **If Y/PY/WN to 1.2**: Were confounding factors that were controlled for (and for which control was necessary) measured validly and reliably by the variables available in this study?	NA / Y / WN (no, but the extent of measurement error in confounding factors was probably not substantial) / SN (no, and the extent of measurement error in confounding factors was probably substantial) / NI	
1.4 **If N/PN/NI to 1.1**: Did the authors control for time-varying factors or other variables measured after the start of the exposure window being studied?	NA / Y / PY / PN / N / NI	
1.5 Did the use of negative controls, or other considerations, suggest uncontrolled confounding?	Y / PY / PN / N	
Risk of bias (due to confounding) in the estimated effect of exposure on the outcome	Low risk / Some concerns / High risk / Very high risk	

Signalling questions	Response options	Comments
What is the predicted direction of bias due to confounding?	Towards benefit of (higher) exposure / Towards harm of (higher) exposure / Towards null / Away from null / Insufficient information available	
Is the risk of bias (due to confounding) sufficiently high, in the context of its likely direction and the magnitude of the estimated exposure effect, to threaten conclusions about whether the exposure has an important effect on the outcome?	Yes / No / Cannot tell	

Y = Yes; PY = Probably yes; PN = Probably no; N = No; SY = Strong yes; WY = Weak yes; SN = Strong no; WN = Weak no; NA = Not applicable; NI = No information

Domain 2: Risk of bias arising from measurement of the exposure

*Domain 2, Variant (a): **If N/PN to C5** (exposure was measured at a single point in time)*

Signalling questions	Response options	Comments
Mismeasurement or misclassification of the exposure.		
2.1 Does the measured exposure well-characterize the exposure metric specified to be of interest in this study? [*This was specified in the answers to D2, D3 and D4*]	SN	Physical activity was assessed using a single self-reported question at baseline and did not capture intensity, duration or changes over time.
2.2 Was the exposure likely to be measured with error, or misclassified?	SY	Physical activity was self-reported using one questionnaire item. Recall error is possible.
Bias in the estimated effect of exposure arising from mismeasurement or misclassification of the exposure		
2.3 If SY/WY to 2.2: Could mismeasurement or misclassification of exposure have been differential (i.e. related to the outcome or risk of the outcome)?	PN	Physical activity was measured before frailty developed. Differential misclassification is unlikely
2.4 If SY/WY to 2.2 and N/PN/WY to 2.3: Is non-differential measurement error likely to bias the estimated effect of exposure on outcome?	WY	Non-differential misclassification is likely to attenuate the association, biasing the estimate towards the null.
Risk of bias (arising from measurement of exposure) in the estimated effect of exposure on the outcome	Some concerns	Exposure was self-reported at baseline using a single questionnaire item
What is the predicted direction of bias arising from measurement of exposure?	Towards null	Any misclassification would likely bias the results towards the null
Is the risk of bias (arising from measurement of exposure) sufficiently high, in the context of its likely direction and the magnitude of the estimated exposure effect, to threaten conclusions about whether the exposure has an important effect on the outcome?	No	The bias is more likely to weaken the association than change the overall conclusion.

Y = Yes; PY = Probably yes; PN = Probably no; N = No; SY = Strong yes; WY = Weak yes; SN = Strong no; WN = Weak no; NA = Not applicable; NI = No information

*Domain 2, Variant (b): **If Y/PY to C5 and Y/PY to C6** (each individual's exposure level was estimated from measurements made at multiple time points)*

Signalling questions	Response options	Comments
2.1 Does the measured exposure (derived from measurements at multiple time points) well-characterize the exposure metric specified to be of interest in this study? [*This was specified in the answers to D2, D3 and D4*]	Y / PY / WN (no, to a small extent) / SN (no, to a large extent) / NI	
2.2 Was there error in measurement, or misclassification, of the exposure, at each single time point?	SY (yes, probably a substantial amount) / WY (yes, but probably not a substantial amount) / PN / N / NI	
2.3 If SY/WY to 2.2: Could mismeasurement or misclassification of exposure have been differential (i.e. related to the outcome or risk of the outcome)?	NA / SY (yes, to a large extent) / WY (yes, to a small extent) / PN / N / NI	
2.4 If SY/WY to 2.2 and N/PN/WY to 2.3: Is the nature of the (non-differential) measurement error likely to bias the estimated effect of exposure on outcome?	NA / SY (yes, to a large extent) / WY (yes, to a small extent) / PN / N / NI	
Risk of bias (arising from measurement of exposure) in the estimated effect of exposure on the outcome	Low risk / Some concerns / High risk / Very high risk	
What is the predicted direction of bias arising from measurement of exposure?	Towards benefit of (higher) exposure / Towards harm of (higher) exposure / Towards null / Away from null / Insufficient information available	
Is the risk of bias (arising from measurement of exposure) sufficiently high, in the context of its likely direction and the magnitude of the estimated exposure effect, to threaten conclusions about whether the exposure has an important effect on the outcome?	Yes / No / Cannot tell	

Y = Yes; PY = Probably yes; SN = Strong no; WN = Weak no; NA = Not applicable; NI = No information

*Domain 2, Variant (c): **If Y/PY to C5, N/PN to C6 and Y/PY to C7** (the analysis was based on splitting participants' follow up time according to exposure status and/or magnitude):*

Signalling questions	Response options	Comments
2.1 Does the measured exposure (including changes over time) well-characterize the exposure metric specified to be of interest in this study? [*This was specified in the answers to D2, D3 and D4*]	Y / PY / WN (no, to a small extent) / SN (no, to a large extent) / NI	
2.2 Was there error in measurement, or misclassification, of the exposure, at each single time point?	SY (yes, probably a substantial amount) / WY (yes, but probably not a substantial amount) / PN / N / NI	
2.3 If SY/WY to 2.2: Could mismeasurement or misclassification of exposure have been differential (i.e. related to the outcome or risk of the outcome)?	NA / SY (yes, to a large extent) / WY (yes, to a small extent) / PN / N / NI	
2.4 If SY/WY to 2.2 and N/PN/WY to 2.3: Is the nature of the (non-differential) measurement error likely to bias the estimated effect of exposure on outcome?	NA / SY (yes, to a large extent) / WY (yes, to a small extent) / PN / N / NI	
Risk of bias (arising from measurement of exposure) in the estimated effect of exposure on the outcome	Low risk / Some concerns / High risk / Very high risk	
What is the predicted direction of bias<br><br>arising from measurement of exposure?	Towards benefit of (higher) exposure / Towards harm of (higher) exposure / Towards null / Away from null / Insufficient information available	
Is the risk of bias (arising from measurement of exposure) sufficiently high, in the context of its likely direction and the magnitude of the estimated exposure effect, to threaten conclusions about whether the exposure has an important effect on the outcome?	Yes / No / Cannot tell	

Y = Yes; PY = Probably yes; SN = Strong no; WN = Weak no; NA = Not applicable; NI = No information

Domain 3: Risk of bias in selection of participants into the study (or into the analysis)

Signalling questions	Response options	Comments
3.1 Did follow-up begin at (or close to) the start of the exposure window for most participants? [*The exposure window is specified in D3*]	Y	Physical activity was measured at baseline. Participants were followed for 3 years
3.2 **If N/PN to 3.1**: Is the effect of exposure likely to be constant over the period of follow up analysed?	PY	The follow-up period was relatively short (3 years), making substantial changes in physical activity less likely.
3.3 Was selection of participants into the study (or into the analysis) based on participant characteristics observed after the start of the exposure window being studied? [*The exposure window is specified in D3*]	N	Participants were selected based on baseline characteristics and exclusion of prevalent frailty before outcome assessment.
3.4 **If Y/PY to 3.3**: Were these characteristics likely to be influenced by exposure or a cause of exposure?	NA	
3.5 **If Y/PY to 3.4**: Were these characteristics likely to be influenced by the outcome or a cause of the outcome?	NA	
3.6 **If N/PN to 3.2 or Y/PY to 3.5**: Is it likely that the analysis corrected for all of the potential selection biases identified in A and B above?	NA	
3.7 **If N/PN to 3.2 or Y/PY to 3.5**: Did sensitivity analyses demonstrate that the likely impact of the potential selection biases identified in A or B above was minimal?	NA	
Risk of bias (due to selection of participants into the study) in the estimated effect of exposure on the outcome	Low risk	Participants with prevalent frailty were excluded at baseline.
What is the predicted direction of bias<br><br>due to selection of participants into the study?	Insufficient information available	No clear direction of bias is expected.
Is the risk of bias (due to selection of participants into the study) sufficiently high, in the context of its likely direction and the magnitude of the estimated exposure effect, to	No	Selection bias is unlikely to have affected the results

**Signalling questions**	**Response options**	**Comments**
threaten conclusions about whether the exposure has an important effect on the outcome?		

Y = Yes; PY = Probably yes; PN = Probably no; N = No; SN = Strong no; WN = Weak no; NA = Not applicable; NI = No information

Domain 4: Risk of bias due to post-exposure interventions

Signalling questions	Response options	Comments
4.1 Were there post-exposure interventions that were influenced by prior exposure during the follow-up period?	N	No post-exposure interventions related to physical activity were reported.
4.2 **If Y/PY to 4.1**: Is it likely that the analysis corrected for the effect of post-exposure interventions that were influenced by prior exposure?	NA	
Risk of bias (due post-exposure interventions) in the estimated effect of exposure on the outcome	Low risk	No post-exposure interventions were identified.
What is the predicted direction of bias due to confounding?	Insufficient information available	No relevant post-exposure interventions were identified; no direction of bias can be predicted.
Is the risk of bias (due post-exposure interventions) sufficiently high, in the context of its likely direction and the magnitude of the estimated exposure effect, to threaten conclusions about whether the exposure has an important effect on the outcome?	No	Post-exposure interventions are unlikely to have affected the results.

Y = Yes; PY = Probably yes; PN = Probably no; N = No; NA = Not applicable; NI = No information

Domain 5: Risk of bias due to missing data

Signalling questions	Response options	Comments
5.1 Were complete data on exposure status available for all, or nearly all, participants?	Y	Baseline physical activity was available for all analysed participants.
5.2 Were complete data on the outcome available for all, or nearly all, participants?	PY	Incident frailty was assessed at follow-up:some loss to follow-up occurred.
5.3 Were complete data on confounding variables available for all, or nearly all, participants?	PY	Confounder information was largely available for participants included in the adjusted analyses.
5.4 **If N/PN/NI to 5.1, 5.2 or 5.3**: Is the result based on a complete case analysis?	NA	Analyses were conducted on participants with complete data; no imputation was reported
5.5 **If Y/PY/NI**: Was exclusion from the analysis because of missing data (in exposure, confounders or the outcome) likely to be related to the true value of the outcome?	PN	There is no evidence that exclusion due to missing data depended on the true frailty outcome.
5.6 **If N/PN to 5.5**: Were all or most predictors of missingness (in exposure, confounders or the outcome) included in the analysis model?	NA	
5.7 **If N/PN to 5.4**: Was the analysis based on imputing missing values?	N	No multiple imputation was reported.
5.8 **If Y/PY to 5.7**: Was imputation performed appropriately?	NA	
5.9 **If N/PN to 5.7**: Was an appropriate alternative method used to correct for bias due to missing data?	NI	No alternative approach to address missing data was reported.
5.10 **If PN/N/NI to 5.1, 5.2 or 5.3**: Is there evidence that the result was not biased by missing data?	PN	Some loss to follow-up occurred. No sensitivity analyses for missing data were report
Risk of bias (due to missing data) in the estimated effect of exposure on the outcome	Some concerns	**Some participants were excluded because of missing data. No imputation or sensitivity analyses were reported.**

Signalling questions	Response options	Comments
What is the predicted direction of bias due to missing data?	Insufficient information available	No clear direction of bias can be identified.
Is the risk of bias (due to missing data) sufficiently high, in the context of its likely direction and the magnitude of the estimated exposure effect, to threaten conclusions about whether the exposure has an important effect on the outcome?	No	Missing data may have introduced some uncertainty. The results are unlikely to be substantially affected.

Y = Yes; PY = Probably yes; PN = Probably no; N = No; SY = Strong yes; WY = Weak yes; NA = Not applicable; NI = No information

Domain 6: Risk of bias arising from measurement of the outcome

Signalling questions	Response options	Comments
6.1 Could measurement or ascertainment of the outcome have differed between exposure groups or levels of exposure?	N	Incident frailty was assessed with the same 39-item Frailty Index for all participants.
6.2 Were outcome assessors aware of study participants' exposure history?	PY	Blinding of outcome assessors was not reported.
6.3 **If Y/PY/NI to 6.2**: Could assessment of the outcome have been influenced by knowledge of participants' exposure history?	PN	Frailty was assessed using a standardized instrument. Differential assessment is unlikely.
Risk of bias (arising from measurement of outcomes) in the estimated effect of exposure on the outcome	Low risk	Outcome assessment was standardized for all participants.
What is the predicted direction of bias arising from measurement of outcomes?	Insufficient information available	No clear direction of bias can be identified
Is the risk of bias (arising from measurement of outcomes) sufficiently high, in the context of its likely direction and the magnitude of the estimated exposure effect, to threaten conclusions about whether the exposure has an important effect on the outcome?	No	Outcome measurement is unlikely to have affected the results

Y = Yes; PY = Probably yes; PN = Probably no; N = No; SY = Strong yes; WY = Weak yes; NA = Not applicable; NI = No information

Domain 7: Risk of bias in selection of the reported result

Signalling questions	Response options	Comments
7.1 Was the result reported in accordance with an available, pre-determined analysis plan?	N	No pre-specified protocol or statistical analysis plan was available
7.2 **If N/PN/NI to 7.1**: Is the reported effect estimate likely to be selected, based on desirability of the magnitude (or statistical significance) of the estimated effect of exposure on outcome, from multiple *exposure measurements* within the exposure domain?	PN	Physical activity was measured once at baseline. Selective reporting from repeated measurements is unlikely
7.3 Is the reported effect estimate likely to be selected, based on desirability of the magnitude (or statistical significance) of the estimated effect of exposure on outcome, from multiple *outcome measurements* within the outcome domain?	PN	Frailty was assessed using a predefined 39-item Frailty Index.
7.4 Is the reported effect estimate likely to be selected, based on desirability of the magnitude (or statistical significance) of the estimated effect of exposure on outcome, from multiple *analyses* of the exposure-outcome relationship?	PN	The reported analyses were consistent with the study objectives. No evidence of selective reporting was found.
7.5 Is the reported effect estimate likely to be selected, based on the basis of desirability of the results (e.g. statistical significance), from different *subgroups*?	N	No subgroup analyses suggesting selective reporting were presented
Risk of bias (due to selection of the reported result) in the estimated effect of exposure on the outcome	Low risk	Although no pre-specified analysis plan was available, there was no evidence that the reported results were selectively chosen from multiple exposure measurements, outcome definitions, analyses or subgroups.
What is the predicted direction of bias due to selection of the reported result?	Insufficient information available	No clear direction of bias can be identified
Is the risk of bias (due to selection of the reported result) sufficiently high, in the context of its likely direction and the magnitude of the estimated exposure effect, to	No	Selective reporting is unlikely to have affected the results

Signalling questions	Response options	Comments
threaten conclusions about whether the exposure has an important effect on the outcome?		

Y = Yes; PY = Probably yes; PN = Probably no; N = No; NA = Not applicable; NI = No information

Overall risk of bias

	Response options	Comments
Overall risk of bias	High risk	High risk was mainly driven by residual confounding. The models adjusted for several sociodemographic variables, but important baseline health-related factors (e.g. functional status and overall health) were not included. Physical activity was also self-reported, and no pre-specified analysis plan was available.
What is the predicted direction of bias?	Towards benefit of (higher) exposure	Residual confounding may have overestimated the protective effect of physical activity, as healthier participants were more likely to be physically active.
Is the overall risk of bias sufficiently high, in the context of its likely direction and the magnitude of the estimated exposure effect, to threaten conclusions about whether the exposure has an important effect on the outcome?	Yes	Residual confounding is likely to have inflated the estimated protective effect of physical activity. This could have influenced the study conclusions.

This work is licensed under a Creative Commons Attribution-NonCommercial-NoDerivatives 4.0 International License.

Fung et al.

For each study result: preliminary considerations

A. Specify the result being assessed for risk of bias

A1. Specify the numerical result being assessed

Hazard ratio (HR) for incident frailty associated with declining physical activity trajectory, extracted from Supplementary Table 2 after multivariable adjustment. This estimate was used in the meta-analysis.

B. Decide whether to proceed with a risk-of-bias assessment

	Response options	Comments
B1. Did the authors make any attempt to control for confounding?	Y	Multivariable Cox models adjusted for demographic, lifestyle, dietary and clinical confounders.
B2. **If N/PN to B1**: Is there sufficient potential for confounding that an unadjusted result should not be considered further?	N	Adjusted estimates were reported and used.
B3. Was the method of measuring exposure inappropriate?	N	Physical activity was assessed repeatedly using a validated questionnaire.
B4. Was the method of measuring the outcome inappropriate?	N	Frailty was assessed repeatedly using the validated FRAIL scale.

If the answer to any of B2, B3 or B4 is 'Yes' or 'Probably yes', the result should be considered to be at very high risk of bias and no further assessment is required. Otherwise, proceed to section C.

C. Specify the analysis in the current study for which results are being assessed for risk of bias

C1. Specify the outcome to which this result relates.

The outcome assessed was incident frailty during follow-up among participants who were non-frail at baseline, as determined using the FRAIL scale.

C2. Specify the participant group on which this result was based.

The result was based on women from the Nurses' Health Study who had repeated physical activity assessments over time and were followed for the development of incident frailty

C3 to C8: Describe the exposure measurement(s) used to produce this result.

C3. What is the exposure being measured and how was it measured or assessed?	The result was based on women from the Nurses' Health Study who had repeated physical activity assessments over time and were followed for the development of incident frailty
C4. Was exposure analysed as a quantitative (rather than a categorical) variable?	N
C5. Did repeated measurements of exposure over time (for each participant) contribute to the analysis that produced this result?	Y
C6. **If Y/PY to C5**, was a single estimate of each participant's exposure level derived from the repeated measurements of exposure over time?	y
C7. **If N/PN to C6**, was the analysis based on splitting participants' follow up time according to exposure status and/or magnitude?	NA
C8. **If Y/PY to C7**, were changes in exposure status and/or magnitude likely to be related to factors that are predictive of the outcome?	NA
C9. **If N/PN to C7**, how were repeat measurements used?	

Y = Yes; PY = Probably yes; PN = Probably no; N = No; NA = Not applicable

C10. Specify the relationship analysed to produce this result. For example, this may be a quadratic relationship of cumulative exposure with the log odds of the outcome, or a risk ratio for the outcome comparing exposed with unexposed individuals.

Hazard ratio (HR) for incident frailty associated with an increase in leisure-time physical activity over time (inclining physical activity), extracted from Supplementary Table 2 after multivariable adjustment. This estimate was used in the meta-analysis.

D: Specify the causal effect of exposure being estimated by this result

D1. Specify the population of interest	Describe eligible participants (to whom the causal effect applies). These may be different from the study participants on whom the result was based (specified in C2). Such differences may give rise to selection biases.	69642 community-dwelling women free of frailty at baseline who were eligible for follow-up assessment of incident frailty.
Specification of the exposure metric of interest		
D2. Specify the exposure	This is the factor whose causal effect on the outcome of interest is the subject of the study result being assessed. It may be thought of as the 'true' exposure of interest. It is distinct from the method with which exposure was measured.	Long-term change in leisure-time physical activity assessed through repeated measurements before incident frailty.
D3. Specify the exposure window	The exposure window of interest is the exposure period for which the result being assessed estimates the effect of exposure on the outcome. Specification of the exposure window is judged by the ROBINS-E user, who should aim to define a window that is both meaningful in answering the review question and broadly in line with when the study measured exposure. Specification should include both the time of onset and period of exposure. For example, it may be lifetime exposure (from birth or from conception), during ages 50-55, the period from first employment in a particular occupation, time from birth to age 10, or during pregnancy.<br><br>The specified exposure window is used to determine whether exposure data adequately reflect exposure during the window. Exposure before the start of the exposure window is addressed during the assessment of risk of bias due to confounding	The exposure window covered repeated assessments of leisure-time physical activity over an 8-year period before the assessment of incident frailty, allowing long-term changes in physical activity to be characterized.

D4. Specify how exposure over time should be summarized	This may, for example, be ever/never exposed, cumulative exposure, average exposure, or peak exposure during the exposure period, for each participant. Alternatively, there may be only a single exposure event, or the exposure may be time invariant (such as a genetic variant or family history).	Exposure was defined as the change in leisure-time physical activity over time and categorized according to the magnitude of increase or decrease, with the inclining trajectory compared with the stable activity group.

E. Evaluation of confounding factors

Complete a row for each important confounding factor listed in advance (subsection (i)). In addition, consider any further confounding factors that are either relevant to the setting of this particular study or which the study authors identified as potentially important (subsection (ii)).

"Important" confounding factors are those for which, in the context of this study, adjustment is expected to lead to an important change in the estimated effect of the exposure.

(i) Important confounding factors listed in advance						
Confounding factor	Measured variable(s) for this factor, if any	Was this variable (or were these variables) controlled for in the analysis? (Y / N)	If this confounding factor was controlled for, was it measured validly and reliably by this variable (or these variables)?* (NA / Y / PY / PN / N / NI)	If this confounding factor was not controlled for, is there evidence that controlling for it was unnecessary?** (NA / Y / PY / PN / N)	Is failure to adjust for this confounding factor expected to bias the effect estimate towards benefit or harm of (higher) exposure?*** (Benefit of (higher) exposure / Harm of (higher) exposure / Insufficient information available)	Comments
Age	Age(years)	Y	Y	NA	Benefit of higher exposure	Age was accurately measured and included in the multivariable model. Older age is associated with both lower physical activity and higher frailty risk.
Sex	NA	NA	NA	NA	NA	Women population
Lifestyle factors	Smoking, alcohol intake, total energy intake, diet quality (AHEI-2010)	Y	PY	NA	Benefit of higher exposure	Major lifestyle factors associated with both physical activity and frailty were included in

						the multivariable models. Diet quality was derived from a validated FFQ.
BMI	Predicted lean body mass	Y	Y	NA	Benefit of higher exposure	Lean body mass was estimated using a validated prediction equation previously validated against DXA measurements
Baseline frailty/functional status	Baseline frailty status	Y	Y	NA	Benefit of higher exposure	Women with prevalent frailty were excluded at baseline, and additional analyses stratified by the number of pre-existing frailty criteria were performed.
Health status / comorbidities	Hormone therapy, aspirin, β-blockers, calcium channel blockers, ACE inhibitors, other antihypertensive drugs, lipid-lowering medication, insulin, oral hypoglycaemic medication	Y	PY	NA	Benefit of higher exposure	Medication use and clinical factors reflecting underlying health status were adjusted for in the multivariable analyses.

Socioeconomic status	Highest educational degree	Y	Y	NA	Benefit of higher exposure	Educational attainment was included as an indicator of socioeconomic status.

(ii) Additional confounding factors relevant to the setting of this particular study, or identified by study authors and considered to be important, or which were identified since the protocol was written						
Confounding factor	Measured variable(s) for this factor, if any	Was this variable (or were these variables) controlled for in the analysis? (Y / N)	If this confounding factor was controlled for, was it measured validly and reliably by this variable (or these variables)?* (NA / Y / PY / PN / N / NI)	If this confounding factor was not controlled for, is there evidence that controlling for it was unnecessary?** (NA / Y / PY / PN / N)	Is failure to adjust for this confounding factor expected to bias the effect estimate towards benefit or harm of (higher) exposure?*** (Benefit of (higher) exposure / Harm of (higher) exposure / Insufficient information available)	Comments
Residual confounding	Unmeasured factors (e.g., inflammatory biomarkers, other health-related behaviours)	N	NA	PN	Benefit of higher exposure	Although an extensive set of demographic, lifestyle and clinical confounders was adjusted for, residual confounding from unmeasured factors cannot be completely excluded.

* "Validity" refers to whether the confounding variable or variables accurately measure the confounding factor, while "reliability" refers to the precision of the measurement (more measurement error means less reliability).

** In the context of a particular study, variables need not be included in the analysis: (a) if they are measured validly and reliably and are not associated with the outcome, conditional on exposure (noting that lack of a statistically significant association is not evidence of a lack of association); (b) if they are measured validly and reliably and are not associated with exposure; (c) if they are measured validly and reliably and adjustment makes no or minimal difference to the estimated effect of the primary parameter; (d) because the confounder was addressed in the study design, for example by restricting to individuals with the

same value of the confounder; (e) because a negative control demonstrates that there was unlikely to have been confounding due to this variable or that uncontrolled confounding was likely to be minimal; or (f) because external evidence suggests that controlling for the variable is not necessary in the context of the study being assessed..

For each study: risk of bias assessment

Domain 1: Risk of bias due to confounding

Domain 1, Variant (a): ***If N/PN to C5 or Y/PY to C6 or N/PN to C7*** *(only baseline confounding needs to be addressed)*

Signalling questions	Response options	Comments
1.1 Did the authors control for all the important confounding factors for which this was necessary?	WN	Most important confounders were adjusted for, but residual confounding cannot be excluded.
1.2 **If Y/PY/WN to 1.1**: Were confounding factors that were controlled for (and for which control was necessary) measured validly and reliably by the variables available in this study?	Y	Confounders were measured using validated instruments or standardized methods.
1.3 **If Y/PY/WN to 1.1**: Did the authors control for any variables after the start of the exposure period being studied that could have been affected by the exposure?	PN	No post-exposure variables appear to have been adjusted for.
1.4 Did the use of negative controls, or other considerations, suggest serious uncontrolled confounding?	PN	No evidence of serious uncontrolled confounding was reported
Risk of bias (due to confounding) in the estimated effect of exposure on the outcome	Some concerns	Most important confounding factors were appropriately adjusted for, but residual confounding from unmeasured variables remains possible.
What is the predicted direction of bias due to confounding?	(Towards benefit of (higher) exposure	Residual confounding may overestimate the protective effect of higher physical activity.
Is the risk of bias (due to confounding) sufficiently high, in the context of its likely direction and the magnitude of the estimated exposure effect, to threaten conclusions about whether the exposure has an important effect on the outcome?	No	Although residual confounding cannot be excluded, it is unlikely to be substantial enough to alter the overall conclusions of the study.

Y = Yes; PY = Probably yes; PN = Probably no; N = No; SY = Strong yes; WY = Weak yes; SN = Strong no; WN = Weak no; NA = Not applicable; NI = No information

*Domain 1, variant (b): **If Y/PY to C7 and Y/PY to C8** (the analysis was based on splitting participants' follow up time according to exposure status and/or magnitude and changes in exposure status and/or magnitude likely to be related to factors that are predictive of the outcome, so both baseline and time-varying confounding need to be addressed)*

**Signalling questions**	**Response options**	**Comments**
1.1 Did the authors use an analysis method that was appropriate to control for time-varying as well as baseline confounding?	Y / PY / PN / N / NI	
1.2 **If Y/PY to 1.1**: Did the authors control for all the important baseline and time-varying confounding factors for which this was necessary?	NA / Y / PY / WN (no, but uncontrolled confounding was probably not substantial) / SN (no, and uncontrolled confounding was probably substantial) / NI	
1.3 **If Y/PY/WN to 1.2**: Were confounding factors that were controlled for (and for which control was necessary) measured validly and reliably by the variables available in this study?	NA / Y / WN (no, but the extent of measurement error in confounding factors was probably not substantial) / SN (no, and the extent of measurement error in confounding factors was probably substantial) / NI	
1.4 **If N/PN/NI to 1.1**: Did the authors control for time-varying factors or other variables measured after the start of the exposure window being studied?	NA / Y / PY / PN / N / NI	
1.5 Did the use of negative controls, or other considerations, suggest uncontrolled confounding?	Y / PY / PN / N	
Risk of bias (due to confounding) in the estimated effect of exposure on the outcome	Low risk / Some concerns / High risk / Very high risk	

Signalling questions	Response options	Comments
What is the predicted direction of bias due to confounding?	Towards benefit of (higher) exposure / Towards harm of (higher) exposure / Towards null / Away from null / Insufficient information available	
Is the risk of bias (due to confounding) sufficiently high, in the context of its likely direction and the magnitude of the estimated exposure effect, to threaten conclusions about whether the exposure has an important effect on the outcome?	Yes / No / Cannot tell	

Y = Yes; PY = Probably yes; PN = Probably no; N = No; SY = Strong yes; WY = Weak yes; SN = Strong no; WN = Weak no; NA = Not applicable; NI = No information

Domain 2: Risk of bias arising from measurement of the exposure

*Domain 2, Variant (a): **If N/PN to C5** (exposure was measured at a single point in time)*

Signalling questions	Response options	Comments
Mismeasurement or misclassification of the exposure.		
2.1 Does the measured exposure well-characterize the exposure metric specified to be of interest in this study? [*This was specified in the answers to D2, D3 and D4*]	Y / PY / WN (no, to a small extent) / SN (no, to a large extent) / NI	
2.2 Was the exposure likely to be measured with error, or misclassified?	SY (yes, probably a substantial amount) / WY (yes, but probably not a substantial amount) / PN / N / NI	
Bias in the estimated effect of exposure arising from mismeasurement or misclassification of the exposure		
2.3 If SY/WY to 2.2: Could mismeasurement or misclassification of exposure have been differential (i.e. related to the outcome or risk of the outcome)?	NA / SY (yes, to a large extent) / WY (yes, to a small extent) / PN / N / NI	
2.4 If SY/WY to 2.2 and N/PN/WY to 2.3: Is non-differential measurement error likely to bias the estimated effect of exposure on outcome?	NA / SY (yes, to a large extent) / WY (yes, to a small extent) / PN / N / NI	
Risk of bias (arising from measurement of exposure) in the estimated effect of exposure on the outcome	Low risk / Some concerns / High risk / Very high risk	
What is the predicted direction of bias arising from measurement of exposure?	Towards benefit of (higher) exposure / Towards harm of (higher) exposure / Towards null / Away from null / Insufficient information available	
Is the risk of bias (arising from measurement of exposure) sufficiently high, in the context of its likely direction and the magnitude of the estimated exposure effect, to	Yes / No / Cannot tell	

Signalling questions	Response options	Comments
threaten conclusions about whether the exposure has an important effect on the outcome?		

Y = Yes; PY = Probably yes; PN = Probably no; N = No; SY = Strong yes; WY = Weak yes; SN = Strong no; WN = Weak no; NA = Not applicable; NI = No information

*Domain 2, Variant (b): **If Y/PY to C5 and Y/PY to C6** (each individual's exposure level was estimated from measurements made at multiple time points)*

Signalling questions	Response options	Comments
2.1 Does the measured exposure (derived from measurements at multiple time points) well-characterize the exposure metric specified to be of interest in this study? [*This was specified in the answers to D2, D3 and D4*]	Y	Repeated assessments of leisure-time physical activity over 20 years using cumulative averages provided a good characterization of long-term exposure.
2.2 Was there error in measurement, or misclassification, of the exposure, at each single time point?	WY	Physical activity was self-reported and therefore subject to recall and reporting error; however, repeated assessments and cumulative averages likely reduced measurement error
2.3 If SY/WY to 2.2: Could mismeasurement or misclassification of exposure have been differential (i.e. related to the outcome or risk of the outcome)?	PN	Participants with frailty at baseline were excluded, and physical activity was assessed before incident frailty occurred, making differential misclassification of exposure unlikely.
2.4 If SY/WY to 2.2 and N/PN/WY to 2.3: Is the nature of the (non-differential) measurement error likely to bias the estimated effect of exposure on outcome?	WY	Any remaining measurement error is likely to be non-differential and would attenuate the observed association between physical activity and frailty.
Risk of bias (arising from measurement of exposure) in the estimated effect of exposure on the outcome	Some concerns	Exposure assessment relied on repeated self-reported questionnaires. Although cumulative averages substantially improved exposure characterization, some measurement error remains possible.
What is the predicted direction of bias arising from measurement of exposure?	Towards null	Non-differential measurement error would most likely bias the estimated association towards the null.
Is the risk of bias (arising from measurement of exposure) sufficiently high, in the context of its likely direction and the magnitude of the estimated exposure effect, to threaten conclusions about whether the exposure has an important effect on the outcome?	No	Repeated exposure assessments over time reduce random measurement error, making substantial bias unlikely.

Y = Yes; PY = Probably yes; SN = Strong no; WN = Weak no; NA = Not applicable; NI = No information

*Domain 2, Variant (c): **If Y/PY to C5, N/PN to C6 and Y/PY to C7** (the analysis was based on splitting participants' follow up time according to exposure status and/or magnitude):*

Signalling questions	Response options	Comments
2.1 Does the measured exposure (including changes over time) well-characterize the exposure metric specified to be of interest in this study? [*This was specified in the answers to D2, D3 and D4*]	Y / PY / WN (no, to a small extent) / SN (no, to a large extent) / NI	
2.2 Was there error in measurement, or misclassification, of the exposure, at each single time point?	SY (yes, probably a substantial amount) / WY (yes, but probably not a substantial amount) / PN / N / NI	
2.3 If SY/WY to 2.2: Could mismeasurement or misclassification of exposure have been differential (i.e. related to the outcome or risk of the outcome)?	NA / SY (yes, to a large extent) / WY (yes, to a small extent) / PN / N / NI	
2.4 If SY/WY to 2.2 and N/PN/WY to 2.3: Is the nature of the (non-differential) measurement error likely to bias the estimated effect of exposure on outcome?	NA / SY (yes, to a large extent) / WY (yes, to a small extent) / PN / N / NI	
Risk of bias (arising from measurement of exposure) in the estimated effect of exposure on the outcome	Low risk / Some concerns / High risk / Very high risk	
What is the predicted direction of bias arising from measurement of exposure?	Towards benefit of (higher) exposure / Towards harm of (higher) exposure / Towards null / Away from null / Insufficient information available	
Is the risk of bias (arising from measurement of exposure) sufficiently high, in the context of its likely direction and the magnitude of the estimated exposure effect, to threaten conclusions about whether the exposure has an important effect on the outcome?	Yes / No / Cannot tell	

Y = Yes; PY = Probably yes; SN = Strong no; WN = Weak no; NA = Not applicable; NI = No information

Domain 3: Risk of bias in selection of participants into the study (or into the analysis)

Signalling questions	Response options	Comments
3.1 Did follow-up begin at (or close to) the start of the exposure window for most participants? [*The exposure window is specified in D3*]	Y	Follow-up began at the start of the exposure window, and participants were followed prospectively after baseline exposure assessment.
3.2 **If N/PN to 3.1**: Is the effect of exposure likely to be constant over the period of follow up analysed?	NA	
3.3 Was selection of participants into the study (or into the analysis) based on participant characteristics observed after the start of the exposure window being studied? [*The exposure window is specified in D3*]	PN	Participant selection was defined at baseline and was not based on characteristics measured after the start of the exposure window.
3.4 **If Y/PY to 3.3**: Were these characteristics likely to be influenced by exposure or a cause of exposure?	NA	
3.5 **If Y/PY to 3.4**: Were these characteristics likely to be influenced by the outcome or a cause of the outcome?	NA	
3.6 **If N/PN to 3.2 or Y/PY to 3.5**: Is it likely that the analysis corrected for all of the potential selection biases identified in A and B above?	NA	
3.7 **If N/PN to 3.2 or Y/PY to 3.5**: Did sensitivity analyses demonstrate that the likely impact of the potential selection biases identified in A or B above was minimal?	WN	Several sensitivity analyses, including analyses excluding women with baseline frailty components and using different lag times, supported the robustness of the findings and suggested that selection bias was unlikely to materially influence the results.
Risk of bias (due to selection of participants into the study) in the estimated effect of exposure on the outcome	Low risk	Participants were enrolled before outcome occurrence, and sensitivity analyses suggested that any selection bias was unlikely to meaningfully affect the estimated association.
What is the predicted direction of bias<br><br>due to selection of participants into the study?	Towards null	Any bias resulting from participant selection or loss to follow-up would most likely attenuate the observed association between physical activity and incident frailty.
Is the risk of bias (due to selection of participants into the study) sufficiently high, in the context of its likely direction	No	Selection bias is unlikely to be substantial enough to alter the overall conclusions of the study.

Signalling questions	Response options	Comments
and the magnitude of the estimated exposure effect, to threaten conclusions about whether the exposure has an important effect on the outcome?		

Y = Yes; PY = Probably yes; PN = Probably no; N = No; SN = Strong no; WN = Weak no; NA = Not applicable; NI = No information

Domain 4: Risk of bias due to post-exposure interventions

Signalling questions	Response options	Comments
4.1 Were there post-exposure interventions that were influenced by prior exposure during the follow-up period?	PN	No post-exposure interventions influenced by prior physical activity were reported during follow-up.
4.2 **If Y/PY to 4.1**: Is it likely that the analysis corrected for the effect of post-exposure interventions that were influenced by prior exposure?	NA	Not applicable because no post-exposure interventions were identified.
Risk of bias (due post-exposure interventions) in the estimated effect of exposure on the outcome	Low risk	No post-exposure interventions related to prior physical activity were reported that could have influenced the association with incident frailty.
What is the predicted direction of bias due to confounding?	Insufficient information available	No meaningful bias from post-exposure interventions is expected because such interventions were not reported.
Is the risk of bias (due post-exposure interventions) sufficiently high, in the context of its likely direction and the magnitude of the estimated exposure effect, to threaten conclusions about whether the exposure has an important effect on the outcome?	No	Bias due to post-exposure interventions is unlikely to have influenced the study findings.

Y = Yes; PY = Probably yes; PN = Probably no; N = No; NA = Not applicable; NI = No information

Domain 5: Risk of bias due to missing data

Signalling questions	Response options	Comments
5.1 Were complete data on exposure status available for all, or nearly all, participants?	Y	Physical activity data were available from repeated questionnaires used to derive long-term exposure.
5.2 Were complete data on the outcome available for all, or nearly all, participants?	PY	Incident frailty was assessed during follow-up, although some participants were lost to follow-up.
5.3 Were complete data on confounding variables available for all, or nearly all, participants?	PY	Confounding variables were largely available and included in multivariable models, with limited missing information reported.
5.4 **If N/PN/NI to 5.1, 5.2 or 5.3**: Is the result based on a complete case analysis?	Y	The primary analyses were based on participants with complete information for the vari[illegible] included in the models
5.5 **If Y/PY/NI**: Was exclusion from the analysis because of missing data (in exposure, confounders or the outcome) likely to be related to the true value of the outcome?	PN	Although some participants were excluded because of missing data or loss to follow-up, [illegible] is no clear evidence that missingness depended on the true frailty outcome.
5.6 **If N/PN to 5.5**: Were all or most predictors of missingness (in exposure, confounders or the outcome) included in the analysis model?	NA	Not applicable because there was no modelling of missingness.
5.7 **If N/PN to 5.4**: Was the analysis based on imputing missing values?	N	No multiple imputation was reported.
5.8 **If Y/PY to 5.7**: Was imputation performed appropriately?	NA	
5.9 **If N/PN to 5.7**: Was an appropriate alternative method used to correct for bias due to missing data?	PY	Sensitivity analyses excluding participants with baseline frailty components and using different lag times partly addressed the potential impact of missing data.
5.10 **If PN/N/NI to 5.1, 5.2 or 5.3**: Is there evidence that the result was not biased by missing data?	PY	Sensitivity analyses produced consistent findings despite some missing data and loss to follow-up, suggesting that missing data were unlikely to have materially biased the results.

Signalling questions	Response options	Comments
Risk of bias (due to missing data) in the estimated effect of exposure on the outcome	Some concerns	Some missing data and loss to follow-up occurred, but extensive sensitivity analyses produced consistent findings, suggesting that missing data were unlikely to materially influence the estimated association.
What is the predicted direction of bias due to missing data?	Towards null	Participants lost to follow-up are likely to have been less healthy, which would tend to attenuate rather than exaggerate the association between physical activity and incident frailty.
Is the risk of bias (due to missing data) sufficiently high, in the context of its likely direction and the magnitude of the estimated exposure effect, to threaten conclusions about whether the exposure has an important effect on the outcome?	No	Although missing data were present, the consistency of the sensitivity analyses suggests that the main conclusions are unlikely to have been materially affected.

Y = Yes; PY = Probably yes; PN = Probably no; N = No; SY = Strong yes; WY = Weak yes; NA = Not applicable; NI = No information

Domain 6: Risk of bias arising from measurement of the outcome

Signalling questions	Response options	Comments
6.1 Could measurement or ascertainment of the outcome have differed between exposure groups or levels of exposure?	PN	Incident frailty was assessed using the validated FRAIL scale, based on standardized questionnaire items that were applied uniformly to all participants regardless of physical activity level.
6.2 Were outcome assessors aware of study participants' exposure history?	PN	Frailty and physical activity were derived from the same cohort questionnaires, so complete blinding of exposure status cannot be assumed.
6.3 **If Y/PY/NI to 6.2**: Could assessment of the outcome have been influenced by knowledge of participants' exposure history?	PN	Frailty was assessed using predefined objective FRAIL criteria, making differential outcome assessment according to exposure unlikely.
Risk of bias (arising from measurement of outcomes) in the estimated effect of exposure on the outcome	Low risk	Incident frailty was assessed using standardized and predefined criteria applied consistently across exposure groups, making measurement bias unlikely.
What is the predicted direction of bias arising from measurement of outcomes?	Insufficient information available	
Is the risk of bias (arising from measurement of outcomes) sufficiently high, in the context of its likely direction and the magnitude of the estimated exposure effect, to threaten conclusions about whether the exposure has an important effect on the outcome?	No	Incident frailty was measured using the validated FRAIL scale with standardized criteria applied consistently across all participants. Although both exposure and outcome were self-reported, differential outcome assessment according to physical activity level is unlikely.

Y = Yes; PY = Probably yes; PN = Probably no; N = No; SY = Strong yes; WY = Weak yes; NA = Not applicable; NI = No information

Domain 7: Risk of bias in selection of the reported result

Signalling questions	Response options	Comments
7.1 Was the result reported in accordance with an available, pre-determined analysis plan?	NI	No pre-published statistical analysis plan or protocol was reported.
7.2 **If N/PN/NI to 7.1**: Is the reported effect estimate likely to be selected, based on desirability of the magnitude (or statistical significance) of the estimated effect of exposure on outcome, from multiple *exposure measurements* within the exposure domain?	PN	Results for repeated physical activity assessments were reported systematically, includi predefined trajectory and change categories.
7.3 Is the reported effect estimate likely to be selected, based on desirability of the magnitude (or statistical significance) of the estimated effect of exposure on outcome, from multiple *outcome measurements* within the outcome domain?	PN	Frailty was assessed as a single predefined outcome using the FRAIL scale.
7.4 Is the reported effect estimate likely to be selected, based on desirability of the magnitude (or statistical significance) of the estimated effect of exposure on outcome, from multiple *analyses* of the exposure-outcome relationship?	PN	Primary analyses and sensitivity analyses were both reported transparently without evi of selective reporting.
7.5 Is the reported effect estimate likely to be selected, based on the basis of desirability of the results (e.g. statistical significance), from different *subgroups*?	PN	Subgroup analyses (e.g., by age and lean body mass) were prespecified exploratory analyses and were reported alongside the primary results.
Risk of bias (due to selection of the reported result) in the estimated effect of exposure on the outcome	Low risk	There was no evidence of selective reporting. Primary, subgroup, and sensitivity analyses were reported transparently and consistently, although no publicly available prespecified analysis plan was identified.
What is the predicted direction of bias due to selection of the reported result?	Insufficient information available	

Signalling questions	Response options	Comments
Is the risk of bias (due to selection of the reported result) sufficiently high, in the context of its likely direction and the magnitude of the estimated exposure effect, to threaten conclusions about whether the exposure has an important effect on the outcome?	No	There is no evidence that selective reporting materially influenced the estimated association between physical activity and incident frailty.

Y = Yes; PY = Probably yes; PN = Probably no; N = No; NA = Not applicable; NI = No information

Overall risk of bias

	Response options	Comments
Overall risk of bias	Some concerns	The study was generally well conducted, with adjustment for major confounders, repeated physical activity assessments and sensitivity analyses. However, some concerns remain due to residual confounding, self-reported physical activity and missing data.
What is the predicted direction of bias?	Insufficient information available	Potential sources of bias may act in different directions. Residual confounding may favour higher physical activity, whereas measurement error and missing data may bias results towards the null. Therefore, the overall direction of bias cannot be determined.
Is the overall risk of bias sufficiently high, in the context of its likely direction and the magnitude of the estimated exposure effect, to threaten conclusions about whether the exposure has an important effect on the outcome?	No	Although some concerns remain, they are unlikely to materially affect the overall study conclusions

This work is licensed under a Creative Commons Attribution-NonCommercial-NoDerivatives 4.0 International License.

Lin et al.

For each study result: preliminary considerations

A. Specify the result being assessed for risk of bias

A1. Specify the numerical result being assessed

Two numerical results were assessed: (1) consistently physically active vs consistently physically inactive trajectory (OR 0.367, 95% CI 0.234–0.575), and (2) inclining physical activity trajectory vs consistently physically inactive trajectory (OR 0.451, 95% CI 0.294–0.691) for frailty at the 2015 follow-up.

B. Decide whether to proceed with a risk-of-bias assessment

	Response options	Comments
B1. Did the authors make any attempt to control for confounding?	y	The analysis adjusted for several baseline covariates, including age, sex, diabetes, chronic kidney disease, oral health, depressive symptoms, life satisfaction and baseline frailty.
B2. **If N/PN to B1**: Is there sufficient potential for confounding that an unadjusted result should not be considered further?	y	Physical activity trajectories are likely to be strongly related to baseline health, functional status and psychosocial factors, which also predict frailty.
B3. Was the method of measuring exposure inappropriate?	PN	Physical activity was self-reported, but repeated assessments over time were used to construct trajectories.
B4. Was the method of measuring the outcome inappropriate?	PN	Frailty was assessed using a multidimensional deficit-based frailty score; although partly self-reported, it was predefined and consistently applied.

If the answer to any of B2, B3 or B4 is 'Yes' or 'Probably yes', the result should be considered to be at very high risk of bias and no further assessment is required. Otherwise, proceed to section C.

C. Specify the analysis in the current study for which results are being assessed for risk of bias

C1. Specify the outcome to which this result relates.

Frailty status at 2015, defined using a deficit-based frailty score with a cutoff point of 30

C2. Specify the participant group on which this result was based.

Community-dwelling Taiwanese adults aged ≥60 years in 1996 from the Taiwan Longitudinal Study of Aging (TLSA), followed through 2015.

C3 to C8: Describe the exposure measurement(s) used to produce this result.

C3. What is the exposure being measured and how was it measured or assessed?	Long-term physical activity pattern, assessed repeatedly through self-reported physical activity across TLSA survey waves and classified into trajectory groups using group-based trajectory modelling.
C4. Was exposure analysed as a quantitative (rather than a categorical) variable?	N
C5. Did repeated measurements of exposure over time (for each participant) contribute to the analysis that produced this result?	Y
C6. **If Y/PY to C5**, was a single estimate of each participant's exposure level derived from the repeated measurements of exposure over time?	Y
C7. **If N/PN to C6**, was the analysis based on splitting participants' follow up time according to exposure status and/or magnitude?	NA
C8. **If Y/PY to C7**, were changes in exposure status and/or magnitude likely to be related to factors that are predictive of the outcome?	NA

C9. **If N/PN to C7**, how were repeat measurements used?

Y = Yes; PY = Probably yes; PN = Probably no; N = No; NA = Not applicable

C10. Specify the relationship analysed to produce this result. For example, this may be a quadratic relationship of cumulative exposure with the log odds of the outcome, or a risk ratio for the outcome comparing exposed with unexposed individuals.

Adjusted odds ratios from multivariable logistic regression comparing the consistently active, inclining and declining physical activity trajectories with the consistently inactive trajectory for frailty at 2015.

D: Specify the causal effect of exposure being estimated by this result

D1. Specify the population of interest	Describe eligible participants (to whom the causal effect applies). These may be different from the study participants on whom the result was based (specified in C2). Such differences may give rise to selection biases.	5,131 community-dwelling Taiwanese adults aged ≥60 years participating in the Taiwan Longitudinal Study of Aging (TLSA).
Specification of the exposure metric of interest		
D2. Specify the exposure	This is the factor whose causal effect on the outcome of interest is the subject of the study result being assessed. It may be thought of as the 'true' exposure of interest. It is distinct from the method with which exposure was measured.	Long-term physical activity trajectory (persistently active, inclining, declining, or persistently inactive).
D3. Specify the exposure window	The exposure window of interest is the exposure period for which the result being assessed estimates the effect of exposure on the outcome. Specification of the exposure window is judged by the ROBINS-E user, who should aim to define a window that is both meaningful in answering the review question and broadly in line with when the study measured exposure. Specification should include both the time of onset and period of exposure. For example, it may be lifetime exposure (from birth or from conception), during ages 50-55, the period from first employment in a particular occupation, time from birth to age 10, or during pregnancy.<br><br>The specified exposure window is used to determine whether exposure data adequately reflect exposure during the window. Exposure before the start of the exposure window is addressed during the assessment of risk of bias due to confounding	Repeated physical activity measurements collected at the 1996, 1999, 2003, 2007, 2011 and 2015 TLSA surveys.
D4. Specify how exposure over time	This may, for example, be ever/never exposed, cumulative exposure, average exposure, or peak exposure during the exposure	Trajectory of physical activity derived from repeated measurements using group-based trajectory modelling (GBTM)

should be summarized	period, for each participant. Alternatively, there may be only a single exposure event, or the exposure may be time invariant (such as a genetic variant or family history).	

E. Evaluation of confounding factors

Complete a row for each important confounding factor listed in advance (subsection (i)). In addition, consider any further confounding factors that are either relevant to the setting of this particular study or which the study authors identified as potentially important (subsection (ii)).

"Important" confounding factors are those for which, in the context of this study, adjustment is expected to lead to an important change in the estimated effect of the exposure.

(i) Important confounding factors listed in advance						
Confounding factor	Measured variable(s) for this factor, if any	Was this variable (or were these variables) controlled for in the analysis?<br>(Y / N)	If this confounding factor was controlled for, was it measured validly and reliably by this variable (or these variables)?*<br>(NA / Y / PY / PN / N / NI)	If this confounding factor was not controlled for, is there evidence that controlling for it was unnecessary?**<br>(NA / Y / PY / PN / N)	Is failure to adjust for this confounding factor expected to bias the effect estimate towards benefit or harm of (higher) exposure?***<br>(Benefit of (higher) exposure / Harm of (higher) exposure / Insufficient information available)	Comments
Age	Age (years)	Y	Y	NA	NA	Included in multivariable model.
Sex	Male/female	Y	Y	NA	NA	Included in multivariable model
BMI	Body mass index	N	NI	PN	Benefit of (higher) exposure	BMI was not included despite being an important confounder of the PA–frailty association.
Baseline frailty/functional status	Baseline fraity score	Y	Y	PN	Benefit of (higher) exposure	Baseline frailty was included in the multivariable logistic regression model.

Chronic diseases/comorbiidities	Diabetes, CKD	Y	PY	NA	NA	Only diabetes mellitus and chronic kidney disease were included in the adjusted model; other chronic diseases were not controlled for
Socioeconomic status	Education ,income	N	N	NA	NA	Education and income were measured but were not included in the final multivariable model.
Lifestyle factors	Smoking and drinking status	N	N	NA	NA	Smoking and drinking status was not included in the final multivariable model.

(ii) Additional confounding factors relevant to the setting of this particular study, or identified by study authors and considered to be important, or which were identified since the protocol was written						
Confounding factor	Measured variable(s) for this factor, if any	Was this variable (or were these variables) controlled for in the analysis? (Y / N)	If this confounding factor was controlled for, was it measured validly and reliably by this variable (or these variables)?* (NA / Y / PY / PN / N / NI)	If this confounding factor was not controlled for, is there evidence that controlling for it was unnecessary?** (NA / Y / PY / PN / N)	Is failure to adjust for this confounding factor expected to bias the effect estimate towards benefit or harm of (higher) exposure?*** (Benefit of (higher) exposure / Harm of (higher) exposure / Insufficient information available)	Comments
Depressive symptoms	CES-D	Y	Y	NA	NA	Included in the adjusted model.

Oral health	OHIP-7	Y	Y	NA	NA	Included in the adjusted model.
Life satisfaction	Questionnaire	Y	PY	NA	NA	Included as a protective factor.

* "Validity" refers to whether the confounding variable or variables accurately measure the confounding factor, while "reliability" refers to the precision of the measurement (more measurement error means less reliability).

** In the context of a particular study, variables need not be included in the analysis: (a) if they are measured validly and reliably and are not associated with the outcome, conditional on exposure (noting that lack of a statistically significant association is not evidence of a lack of association); (b) if they are measured validly and reliably and are not associated with exposure; (c) if they are measured validly and reliably and adjustment makes no or minimal difference to the estimated effect of the primary parameter; (d) because the confounder was addressed in the study design, for example by restricting to individuals with the same value of the confounder; (e) because a negative control demonstrates that there was unlikely to have been confounding due to this variable or that uncontrolled confounding was likely to be minimal; or (f) because external evidence suggests that controlling for the variable is not necessary in the context of the study being assessed..

For each study: risk of bias assessment

Domain 1: Risk of bias due to confounding

Domain 1, Variant (a): ***If N/PN to C5 or Y/PY to C6 or N/PN to C7*** *(only baseline confounding needs to be addressed)*

Signalling questions	Response options	Comments
1.1 Did the authors control for all the important confounding factors for which this was necessary?	WN	The analyses adjusted for age, sex, baseline frailty, diabetes, chronic kidney disease, oral health, depressive symptoms and life satisfaction. However, important confounders such as BMI, education, income, smoking and alcohol consumption were not included in the final model. .
1.2 **If Y/PY/WN to 1.1**: Were confounding factors that were controlled for (and for which control was necessary) measured validly and reliably by the variables available in this study?	Y	Most adjusted confounders were measured using standardized questionnaires and validated instruments (e.g., CES-D, OHIP and baseline frailty assessment)
1.3 **If Y/PY/WN to 1.1**: Did the authors control for any variables after the start of the exposure period being studied that could have been affected by the exposure?	PY	Baseline frailty was included in the adjusted model, but it appears to have been measured after the physical activity trajectory had already begun, meaning it may have been influenced by prior exposure.
1.4 Did the use of negative controls, or other considerations, suggest serious uncontrolled confounding?	PN	No negative control analyses were reported. Although some lifestyle confounders were not included, there is no direct evidence of serious uncontrolled confounding
Risk of bias (due to confounding) in the estimated effect of exposure on the outcome	Some concerns	The study adjusted for several important confounders, including age, sex, baseline frailty, diabetes, chronic kidney disease, depressive symptoms and life satisfaction. However, potentially important confounders such as BMI, education, income, smoking and alcohol consumption were not included in the final model. In addition, baseline frailty was included as an adjustment variable, although it appears to have been measured after the start of the physical activity trajectory, raising concerns about adjustment for a post-exposure variable.

Signalling questions	Response options	Comments
What is the predicted direction of bias due to confounding?	Towards benefit of (higher) exposure	Residual confounding from unmeasured lifestyle and socioeconomic factors may have overestimated the protective association between higher physical activity and frailty. In addition, adjustment for baseline frailty measured after exposure began may have influenced the estimated association
Is the risk of bias (due to confounding) sufficiently high, in the context of its likely direction and the magnitude of the estimated exposure effect, to threaten conclusions about whether the exposure has an important effect on the outcome?	No	Although residual confounding and the timing of baseline frailty assessment raise some methodological concerns, the study adjusted for several key confounders and these limitations are unlikely to be sufficient to overturn the overall conclusions

Y = Yes; PY = Probably yes; PN = Probably no; N = No; SY = Strong yes; WY = Weak yes; SN = Strong no; WN = Weak no; NA = Not applicable; NI = No information

*Domain 1, variant (b): **If Y/PY to C7 and Y/PY to C8** (the analysis was based on splitting participants' follow up time according to exposure status and/or magnitude and changes in exposure status and/or magnitude likely to be related to factors that are predictive of the outcome, so both baseline and time-varying confounding need to be addressed)*

**Signalling questions**	**Response options**	**Comments**
1.1 Did the authors use an analysis method that was appropriate to control for time-varying as well as baseline confounding?	Y / PY / PN / N / NI	
1.2 **If Y/PY to 1.1**: Did the authors control for all the important baseline and time-varying confounding factors for which this was necessary?	NA / Y / PY / WN (no, but uncontrolled confounding was probably not substantial) / SN (no, and uncontrolled confounding was probably substantial) / NI	
1.3 **If Y/PY/WN to 1.2**: Were confounding factors that were controlled for (and for which control was necessary) measured validly and reliably by the variables available in this study?	NA / Y / WN (no, but the extent of measurement error in confounding factors was probably not substantial) / SN (no, and the extent of measurement error in confounding factors was probably substantial) / NI	
1.4 **If N/PN/NI to 1.1**: Did the authors control for time-varying factors or other variables measured after the start of the exposure window being studied?	NA / Y / PY / PN / N / NI	
1.5 Did the use of negative controls, or other considerations, suggest uncontrolled confounding?	Y / PY / PN / N	
Risk of bias (due to confounding) in the estimated effect of exposure on the outcome	Low risk / Some concerns / High risk / Very high risk	

Signalling questions	Response options	Comments
What is the predicted direction of bias due to confounding?	Towards benefit of (higher) exposure / Towards harm of (higher) exposure / Towards null / Away from null / Insufficient information available	
Is the risk of bias (due to confounding) sufficiently high, in the context of its likely direction and the magnitude of the estimated exposure effect, to threaten conclusions about whether the exposure has an important effect on the outcome?	Yes / No / Cannot tell	

Y = Yes; PY = Probably yes; PN = Probably no; N = No; SY = Strong yes; WY = Weak yes; SN = Strong no; WN = Weak no; NA = Not applicable; NI = No information

Domain 2: Risk of bias arising from measurement of the exposure

*Domain 2, Variant (a): **If N/PN to C5** (exposure was measured at a single point in time)*

Signalling questions	Response options	Comments
Mismeasurement or misclassification of the exposure.		
2.1 Does the measured exposure well-characterize the exposure metric specified to be of interest in this study? [*This was specified in the answers to D2, D3 and D4*]	Y / PY / WN (no, to a small extent) / SN (no, to a large extent) / NI	
2.2 Was the exposure likely to be measured with error, or misclassified?	SY (yes, probably a substantial amount) / WY (yes, but probably not a substantial amount) / PN / N / NI	
Bias in the estimated effect of exposure arising from mismeasurement or misclassification of the exposure		
2.3 If SY/WY to 2.2: Could mismeasurement or misclassification of exposure have been differential (i.e. related to the outcome or risk of the outcome)?	NA / SY (yes, to a large extent) / WY (yes, to a small extent) / PN / N / NI	
2.4 If SY/WY to 2.2 and N/PN/WY to 2.3: Is non-differential measurement error likely to bias the estimated effect of exposure on outcome?	NA / SY (yes, to a large extent) / WY (yes, to a small extent) / PN / N / NI	
Risk of bias (arising from measurement of exposure) in the estimated effect of exposure on the outcome	Low risk / Some concerns / High risk / Very high risk	
What is the predicted direction of bias arising from measurement of exposure?	Towards benefit of (higher) exposure / Towards harm of (higher) exposure / Towards null / Away from null / Insufficient information available	
Is the risk of bias (arising from measurement of exposure) sufficiently high, in the context of its likely direction and the magnitude of the estimated exposure effect, to	Yes / No / Cannot tell	

Signalling questions	Response options	Comments
threaten conclusions about whether the exposure has an important effect on the outcome?		

Y = Yes; PY = Probably yes; PN = Probably no; N = No; SY = Strong yes; WY = Weak yes; SN = Strong no; WN = Weak no; NA = Not applicable; NI = No information

*Domain 2, Variant (b): **If Y/PY to C5 and Y/PY to C6** (each individual's exposure level was estimated from measurements made at multiple time points)*

Signalling questions	Response options	Comments
2.1 Does the measured exposure (derived from measurements at multiple time points) well-characterize the exposure metric specified to be of interest in this study? [*This was specified in the answers to D2, D3 and D4*]	Y	Physical activity was assessed repeatedly at six time points (1996–2015) using the same standardized questionnaire, allowing classification of long-term trajectories.
2.2 Was there error in measurement, or misclassification, of the exposure, at each single time point?	WY	Physical activity was self-reported, which may have introduced some recall and reporting error at each assessment, although the same questionnaire was used across all time points.
2.3 If SY/WY to 2.2: Could mismeasurement or misclassification of exposure have been differential (i.e. related to the outcome or risk of the outcome)?	**PN**	Exposure assessment was performed before the outcome assessment using the same procedure for all participants; differential misclassification is unlikely.
2.4 If SY/WY to 2.2 and N/PN/WY to 2.3: Is the nature of the (non-differential) measurement error likely to bias the estimated effect of exposure on outcome?	PN	Any exposure misclassification is likely to be non-differential and would probably not substantially bias the estimated association.
Risk of bias (arising from measurement of exposure) in the estimated effect of exposure on the outcome	Some concerns	Exposure assessment relied on self-report, but repeated measurements substantially improved exposure characterization over time.
What is the predicted direction of bias arising from measurement of exposure?	Towards null	Non-differential misclassification from self-reported physical activity would be expected to attenuate the true association.
Is the risk of bias (arising from measurement of exposure) sufficiently high, in the context of its likely direction and the magnitude of the estimated exposure effect, to threaten conclusions about whether the exposure has an important effect on the outcome?	No	Any exposure misclassification is unlikely to be sufficient to threaten the overall conclusions of the study.

Y = Yes; PY = Probably yes; SN = Strong no; WN = Weak no; NA = Not applicable; NI = No information

*Domain 2, Variant (c): **If Y/PY to C5, N/PN to C6 and Y/PY to C7** (the analysis was based on splitting participants' follow up time according to exposure status and/or magnitude):*

**Signalling questions**	**Response options**	**Comments**
2.1 Does the measured exposure (including changes over time) well-characterize the exposure metric specified to be of interest in this study? [*This was specified in the answers to D2, D3 and D4*]	Y / PY / WN (no, to a small extent) / SN (no, to a large extent) / NI	
2.2 Was there error in measurement, or misclassification, of the exposure, at each single time point?	SY (yes, probably a substantial amount) / WY (yes, but probably not a substantial amount) / PN / N / NI	
2.3 If SY/WY to 2.2: Could mismeasurement or misclassification of exposure have been differential (i.e. related to the outcome or risk of the outcome)?	NA / SY (yes, to a large extent) / WY (yes, to a small extent) / PN / N / NI	
2.4 If SY/WY to 2.2 and N/PN/WY to 2.3: Is the nature of the (non-differential) measurement error likely to bias the estimated effect of exposure on outcome?	NA / SY (yes, to a large extent) / WY (yes, to a small extent) / PN / N / NI	
Risk of bias (arising from measurement of exposure) in the estimated effect of exposure on the outcome	Low risk / Some concerns / High risk / Very high risk	
What is the predicted direction of bias<br><br>arising from measurement of exposure?	Towards benefit of (higher) exposure / Towards harm of (higher) exposure / Towards null / Away from null / Insufficient information available	
Is the risk of bias (arising from measurement of exposure) sufficiently high, in the context of its likely direction and the magnitude of the estimated exposure effect, to threaten conclusions about whether the exposure has an important effect on the outcome?	Yes / No / Cannot tell	

Y = Yes; PY = Probably yes; SN = Strong no; WN = Weak no; NA = Not applicable; NI = No information

Domain 3: Risk of bias in selection of participants into the study (or into the analysis)

Signalling questions	Response options	Comments
3.1 Did follow-up begin at (or close to) the start of the exposure window for most participants? [*The exposure window is specified in D3*]	Y	Follow-up began at the start of the physical activity trajectory period (1996), from which repeated exposure measurements were collected.
3.2 **If N/PN to 3.1**: Is the effect of exposure likely to be constant over the period of follow up analysed?	NA	
3.3 Was selection of participants into the study (or into the analysis) based on participant characteristics observed after the start of the exposure window being studied? [*The exposure window is specified in D3*]	PN	Participants were selected from the original cohort and the analysis was based on those with available longitudinal data; there is no clear evidence that selection was based on post-exposure participant characteristics.
3.4 **If Y/PY to 3.3**: Were these characteristics likely to be influenced by exposure or a cause of exposure?	NA	NA
3.5 **If Y/PY to 3.4**: Were these characteristics likely to be influenced by the outcome or a cause of the outcome?	NA	NA
3.6 **If N/PN to 3.2 or Y/PY to 3.5**: Is it likely that the analysis corrected for all of the potential selection biases identified in A and B above?	NA	NA
3.7 **If N/PN to 3.2 or Y/PY to 3.5**: Did sensitivity analyses demonstrate that the likely impact of the potential selection biases identified in A or B above was minimal?	NA	NA
Risk of bias (due to selection of participants into the study) in the estimated effect of exposure on the outcome	Low risk	Participants were recruited from a well-defined longitudinal cohort, and there was no clear evidence that inclusion in the analysis was based on post-exposure participant characteristics likely to introduce selection bias
What is the predicted direction of bias<br><br>due to selection of participants into the study?	Insufficient information available	There is no evidence that selection into the analysis introduced systematic bias in a specific direction.
Is the risk of bias (due to selection of participants into the study) sufficiently high, in the context of its likely direction and the magnitude of the estimated exposure effect, to	No	There is no evidence that selection into the analysis materially biased the estimated association between physical activity trajectories and frailty.

Signalling questions	Response options	Comments
threaten conclusions about whether the exposure has an important effect on the outcome?		

Y = Yes; PY = Probably yes; PN = Probably no; N = No; SN = Strong no; WN = Weak no; NA = Not applicable; NI = No information

Domain 4: Risk of bias due to post-exposure interventions

**Signalling questions**	**Response options**	**Comments**
4.1 Were there post-exposure interventions that were influenced by prior exposure during the follow-up period?	N	No post-exposure interventions influenced by prior physical activity trajectories were reported during follow-up.
4.2 **If Y/PY to 4.1**: Is it likely that the analysis corrected for the effect of post-exposure interventions that were influenced by prior exposure?	NA	
Risk of bias (due post-exposure interventions) in the estimated effect of exposure on the outcome	Low risk	No evidence of post-exposure interventions likely to affect the association between physical activity trajectories and frailty.
What is the predicted direction of bias due to confounding?	NA	Not applicable, as no relevant post-exposure interventions were identified.
Is the risk of bias (due post-exposure interventions) sufficiently high, in the context of its likely direction and the magnitude of the estimated exposure effect, to threaten conclusions about whether the exposure has an important effect on the outcome?	No	Bias due to post-exposure interventions is unlikely to threaten the validity of the estimated association

Y = Yes; PY = Probably yes; PN = Probably no; N = No; NA = Not applicable; NI = No information

Domain 5: Risk of bias due to missing data

Signalling questions	Response options	Comments
5.1 Were complete data on exposure status available for all, or nearly all, participants?	PN	Physical activity trajectories required repeated measurements; complete exposure data were not available for all original participants.
5.2 Were complete data on the outcome available for all, or nearly all, participants?	PN	Frailty was assessed only among participants with follow-up data in 2015.
5.3 Were complete data on confounding variables available for all, or nearly all, participants?	PY	Most confounders were available for participants included in the analysis.
5.4 **If N/PN/NI to 5.1, 5.2 or 5.3**: Is the result based on a complete case analysis?	Y	The analysis was conducted on participants with complete longitudinal data and outcome assessment
5.5 **If Y/PY/NI**: Was exclusion from the analysis because of missing data (in exposure, confounders or the outcome) likely to be related to the true value of the outcome?	WY	Participants lost to follow-up or deceased may have differed in frailty risk.
5.6 **If N/PN to 5.5**: Were all or most predictors of missingness (in exposure, confounders or the outcome) included in the analysis model?	PN	No formal analysis accounting for predictors of missingness was reported
5.7 **If N/PN to 5.4**: Was the analysis based on imputing missing values?	N	No imputation methods were reported.
5.8 **If Y/PY to 5.7**: Was imputation performed appropriately?	NA	
5.9 **If N/PN to 5.7**: Was an appropriate alternative method used to correct for bias due to missing data?	PN	No alternative statistical approach to address missing data was reported.
5.10 **If PN/N/NI to 5.1, 5.2 or 5.3**: Is there evidence that the result was not biased by missing data?	PN	Although participant flow was clearly reported, no statistical methods or sensitivity analyses were presented to demonstrate that missing data did not influence the results.

Signalling questions	Response options	Comments
Risk of bias (due to missing data) in the estimated effect of exposure on the outcome	Some concerns	Attrition occurred during long-term follow-up and the analysis was based on complete cases. Although participant flow was clearly reported, no formal methods to address missing data were applied.
What is the predicted direction of bias due to missing data?	Towards benefit of (higher) exposure	Participants lost to follow-up were likely to be older, frailer, and less physically active, which may have exaggerated the apparent protective effect of higher physical activity trajectories.
Is the risk of bias (due to missing data) sufficiently high, in the context of its likely direction and the magnitude of the estimated exposure effect, to threaten conclusions about whether the exposure has an important effect on the outcome?	No	Missing data may have introduced some uncertainty, but there is insufficient evidence that they substantially threatened the validity of the estimated association.

Y = Yes; PY = Probably yes; PN = Probably no; N = No; SY = Strong yes; WY = Weak yes; NA = Not applicable; NI = No information

Domain 6: Risk of bias arising from measurement of the outcome

**Signalling questions**	**Response options**	**Comments**
6.1 Could measurement or ascertainment of the outcome have differed between exposure groups or levels of exposure?	PN	Frailty was assessed using the same standardized multidimensional instrument and identical procedures across all physical activity trajectory groups.
6.2 Were outcome assessors aware of study participants' exposure history?	NI	The study does not report whether outcome assessors were blinded to participan physical activity trajectories.
6.3 **If Y/PY/NI to 6.2**: Could assessment of the outcome have been influenced by knowledge of participants' exposure history?	PN	Although blinding was not reported, outcome assessment followed a standardized protocol and differential assessment according to exposure history is unlikely.
Risk of bias (arising from measurement of outcomes) in the estimated effect of exposure on the outcome	Low risk	Frailty was measured using the same validated multidimensional instrument for all participants at follow-up. There is no evidence that outcome measurement differed between physical activity trajectory groups.
What is the predicted direction of bias arising from measurement of outcomes?	Towards null	Any non-differential misclassification of frailty would most likely attenuate the association between physical activity trajectories and frailty
Is the risk of bias (arising from measurement of outcomes) sufficiently high, in the context of its likely direction and the magnitude of the estimated exposure effect, to threaten conclusions about whether the exposure has an important effect on the outcome?	No	There is no evidence that outcome measurement bias was substantial enough to threaten the validity of the estimated association

Y = Yes; PY = Probably yes; PN = Probably no; N = No; SY = Strong yes; WY = Weak yes; NA = Not applicable; NI = No information

Domain 7: Risk of bias in selection of the reported result

Signalling questions	Response options	Comments
7.1 Was the result reported in accordance with an available, pre-determined analysis plan?	NI	No study protocol or pre-specified statistical analysis plan was available
7.2 **If N/PN/NI to 7.1**: Is the reported effect estimate likely to be selected, based on desirability of the magnitude (or statistical significance) of the estimated effect of exposure on outcome, from multiple *exposure measurements* within the exposure domain?	PN	Physical activity trajectories were predefined using repeated assessments across the follow-up period.
7.3 Is the reported effect estimate likely to be selected, based on desirability of the magnitude (or statistical significance) of the estimated effect of exposure on outcome, from multiple *outcome measurements* within the outcome domain?	PN	Frailty was assessed at follow-up using a single predefined multidimensional measure.
7.4 Is the reported effect estimate likely to be selected, based on desirability of the magnitude (or statistical significance) of the estimated effect of exposure on outcome, from multiple *analyses* of the exposure-outcome relationship?	PN	The primary multivariable logistic regression analysis was clearly described and consistently reported.
7.5 Is the reported effect estimate likely to be selected, based on the basis of desirability of the results (e.g. statistical significance), from different *subgroups*?	PN	There was no evidence that results were selectively reported according to statistical significance.
Risk of bias (due to selection of the reported result) in the estimated effect of exposure on the outcome	Low risk	There was no evidence of selective reporting of exposure, outcome, or analytical results. Although a pre-specified analysis plan was not available, the analyses appeared consistent with the study objectives.
What is the predicted direction of bias due to selection of the reported result?	Insufficient information available	No evidence of selective reporting was identified, and any potential direction of bias cannot be determined.
Is the risk of bias (due to selection of the reported result) sufficiently high, in the context of its likely direction and the magnitude of the estimated exposure effect, to	No	There is no evidence that selection of the reported results threatened the validity of the estimated association.

Signalling questions	Response options	Comments
threaten conclusions about whether the exposure has an important effect on the outcome?		

Y = Yes; PY = Probably yes; PN = Probably no; N = No; NA = Not applicable; NI = No information

Overall risk of bias

	Response options	Comments
Overall risk of bias	Some concerns	The main concerns arise from residual confounding (e.g., lack of adjustment for BMI and baseline frailty) and missing data due to long-term follow-up. However, no major concerns were identified in the remaining domains.
What is the predicted direction of bias?	Towards benefit of (higher) exposure	Residual confounding and selective loss to follow-up are more likely to exaggerate the protective association between higher physical activity trajectories and lower frailty risk.
Is the overall risk of bias sufficiently high, in the context of its likely direction and the magnitude of the estimated exposure effect, to threaten conclusions about whether the exposure has an important effect on the outcome?	No	Although some concerns remain regarding residual confounding and missing data, there is insufficient evidence that these biases were substantial enough to threaten the overall validity of the estimated association.

This work is licensed under a Creative Commons Attribution-NonCommercial-NoDerivatives 4.0 International License.

Jung et al.

preliminary considerations

A. Specify the result being assessed for risk of bias

A1. Specify the numerical result being assessed

Association between changes in physical activity and incident frailty estimated using multivariable generalized estimating equation (GEE) logistic regression, with the persistently inactive group as the reference category.

B. Decide whether to proceed with a risk-of-bias assessment

	Response options	Comments
B1. Did the authors make any attempt to control for confounding?	y	Adjusted for age, sex, education, smoking, alcohol use, BMI, chronic diseases, depressive symptoms and physical function.
B2. **If N/PN to B1**: Is there sufficient potential for confounding that an unadjusted result should not be considered further?	NA	
B3. Was the method of measuring exposure inappropriate?	N	Physical activity and frailty share common causes. The adjusted estimate was used.
B4. Was the method of measuring the outcome inappropriate?	N	Incident frailty was assessed using a predefined frailty index based on 34 age-related health deficits. The outcome assessment method was appropriate and applied consistently across participants.

If the answer to any of B2, B3 or B4 is 'Yes' or 'Probably yes', the result should be considered to be at very high risk of bias and no further assessment is required. Otherwise, proceed to section C.

C. Specify the analysis in the current study for which results are being assessed for risk of bias

C1. Specify the outcome to which this result relates.

Incident frailty

C2. Specify the participant group on which this result was based.

5,594 community-dwelling middle-aged and older adults from the Korean Longitudinal Study of Aging (KLoSA) who were free of frailty at baseline.

C3 to C8: Describe the exposure measurement(s) used to produce this result.

C3. What is the exposure being measured and how was it measured or assessed?	Changes in physical activity over time, assessed using repeated self-reported physical activity questionnaires (2006–2022) and classified into physical activity change groups (persistently inactive, decreasing, increasing, persistently active).
C4. Was exposure analysed as a quantitative (rather than a categorical) variable?	N
C5. Did repeated measurements of exposure over time (for each participant) contribute to the analysis that produced this result?	Y
C6. **If Y/PY to C5**, was a single estimate of each participant's exposure level derived from the repeated measurements of exposure over time?	Y
C7. **If N/PN to C6**, was the analysis based on splitting participants' follow up time according to exposure status and/or magnitude?	NA
C8. **If Y/PY to C7**, were changes in exposure status and/or magnitude likely to be related to factors that are predictive of the outcome?	NA

C9. **If N/PN to C7**, how were repeat measurements used?

Y = Yes; PY = Probably yes; PN = Probably no; N = No; NA = Not applicable

C10. Specify the relationship analysed to produce this result. For example, this may be a quadratic relationship of cumulative exposure with the log odds of the outcome, or a risk ratio for the outcome comparing exposed with unexposed individuals.

Association between changes in physical activity (2006–2022) and frailty status in 2022, estimated using generalized estimating equation (GEE) logistic regression adjusted for potential confounders.

D: Specify the causal effect of exposure being estimated by this result

D1. Specify the population of interest	Describe eligible participants (to whom the causal effect applies). These may be different from the study participants on whom the result was based (specified in C2). Such differences may give rise to selection biases.	5594 community-dwelling middle-aged and older Korean adults aged ≥45 years, free of frailty at baseline, for whom the causal effect of long-term changes in physical activity on incident frailty is being estimated.
Specification of the exposure metric of interest		
D2. Specify the exposure	This is the factor whose causal effect on the outcome of interest is the subject of the study result being assessed. It may be thought of as the 'true' exposure of interest. It is distinct from the method with which exposure was measured.	Long-term changes in physical activity, classified into four trajectories: persistently inactive, decreased, increased, and persistently active.
D3. Specify the exposure window	The exposure window of interest is the exposure period for which the result being assessed estimates the effect of exposure on the outcome. Specification of the exposure window is judged by the ROBINS-E user, who should aim to define a window that is both meaningful in answering the review question and broadly in line with when the study measured exposure. Specification should include both the time of onset and period of exposure. For example, it may be lifetime exposure (from birth or from conception), during ages 50-55, the period from first employment in a particular occupation, time from birth to age 10, or during pregnancy.<br><br>The specified exposure window is used to determine whether exposure data adequately reflect exposure during the window. Exposure before the start of the exposure window is addressed during the assessment of risk of bias due to confounding	Physical activity changes assessed between 2006 and 2022 using repeated measurements across the KLoSA waves. Participants were classified according to their long-term physical activity trajectory over the study period.
D4. Specify how exposure over time	This may, for example, be ever/never exposed, cumulative exposure, average exposure, or peak exposure during the exposure period, for each participant. Alternatively, there may be only a	Exposure was modelled as longitudinal trajectories of physical activity based on repeated assessments over time rather than a single baseline measurement.

should be summarized	single exposure event, or the exposure may be time invariant (such as a genetic variant or family history).	

E. Evaluation of confounding factors

Complete a row for each important confounding factor listed in advance (subsection (i)). In addition, consider any further confounding factors that are either relevant to the setting of this particular study or which the study authors identified as potentially important (subsection (ii)).

"Important" confounding factors are those for which, in the context of this study, adjustment is expected to lead to an important change in the estimated effect of the exposure.

(i) Important confounding factors listed in advance						
Confounding factor	Measured variable(s) for this factor, if any	Was this variable (or were these variables) controlled for in the analysis?<br>(Y / N)	If this confounding factor was controlled for, was it measured validly and reliably by this variable (or these variables)?*<br>(NA / Y / PY / PN / N / NI)	If this confounding factor was not controlled for, is there evidence that controlling for it was unnecessary?**<br>(NA / Y / PY / PN / N)	Is failure to adjust for this confounding factor expected to bias the effect estimate towards benefit or harm of (higher) exposure?***<br>(Benefit of (higher) exposure / Harm of (higher) exposure / Insufficient information available)	Comments
Age	Age groups (45–54, 55–64, 65–74, 75–84, ≥85 years)	Y	Y	NA	Towards benefit of higher exposure	Age was measured at baseline and included as an adjustment variable in all multivariable GEE models.
Sex	Male/Female	Y	Y	NA	Towards benefit of higher exposure	Sex was measured at baseline and adjusted for in the primary analyses; subgroup analyses were also stratified by sex.
BMI	Body mass index	N	NA	PN	Towards benefit of higher exposure	BMI was incorporated as one of the deficits composing the

						Frailty Index and was therefore not adjusted for separately.
Baseline frailty status	(FI)	Y	Y	NA	Towards benefit of higher exposure	Participants classified as frail at baseline (FI≥0.25) were excluded before follow-up, restricting analyses to non-frail individuals.
Lifestyle factors	Smoking status, alcohol intake	Y	Y	NA	Towards benefit of higher exposure	Smoking and alcohol consumption were measured at baseline and included as covariates in the adjusted analyses.
Comorbidities	Chronic conditions (included in FI)	N	NA	PN	Towards benefit of higher exposure	Chronic diseases contributed to the Frailty Index outcome and were therefore not entered as separate adjustment variables.
Socioeconomic status	Region, marital status, education, employment, income, participation in social activities	Y	Y	NA	Towards benefit of higher exposure	Sociodemographic characteristics were measured at baseline and adjusted for in the multivariable models.

(ii) Additional confounding factors relevant to the setting of this particular study, or identified by study authors and considered to be important, or which were identified since the protocol was written

Confounding factor	Measured variable(s) for this factor, if any	Was this variable (or were these variables) controlled for in the analysis? (Y / N)	If this confounding factor was controlled for, was it measured validly and reliably by this variable (or these variables)?* (NA / Y / PY / PN / N / NI)	If this confounding factor was not controlled for, is there evidence that controlling for it was unnecessary?** (NA / Y / PY / PN / N)	Is failure to adjust for this confounding factor expected to bias the effect estimate towards benefit or harm of (higher) exposure?*** (Benefit of (higher) exposure / Harm of (higher) exposure / Insufficient information available)	Comments

* "Validity" refers to whether the confounding variable or variables accurately measure the confounding factor, while "reliability" refers to the precision of the measurement (more measurement error means less reliability).

** In the context of a particular study, variables need not be included in the analysis: (a) if they are measured validly and reliably and are not associated with the outcome, conditional on exposure (noting that lack of a statistically significant association is not evidence of a lack of association); (b) if they are measured validly and reliably and are not associated with exposure; (c) if they are measured validly and reliably and adjustment makes no or minimal difference to the estimated effect of the primary parameter; (d) because the confounder was addressed in the study design, for example by restricting to individuals with the same value of the confounder; (e) because a negative control demonstrates that there was unlikely to have been confounding due to this variable or that uncontrolled confounding was likely to be minimal; or (f) because external evidence suggests that controlling for the variable is not necessary in the context of the study being assessed..

For each study: risk of bias assessment

Domain 1: Risk of bias due to confounding

Domain 1, Variant (a): ***If N/PN to C5 or Y/PY to C6 or N/PN to C7*** *(only baseline confounding needs to be addressed)*

**Signalling questions**	**Response options**	**Comments**
1.1 Did the authors control for all the important confounding factors for which this was necessary?	PY	Adjusted for major sociodemographic and lifestyle confounders, including age, sex, smoking, alcohol use, BMI and physical function..
1.2 **If Y/PY/WN to 1.1**: Were confounding factors that were controlled for (and for which control was necessary) measured validly and reliably by the variables available in this study?	PY	Most confounders were collected using the KLoSA protocol. Several variables were self-reported.
1.3 **If Y/PY/WN to 1.1**: Did the authors control for any variables after the start of the exposure period being studied that could have been affected by the exposure?	N	All covariates were measured before the exposure period. No post-exposure variables were adjusted for.
1.4 Did the use of negative controls, or other considerations, suggest serious uncontrolled confounding?	N	No negative control analyses were reported.
Risk of bias (due to confounding) in the estimated effect of exposure on the outcome	Some concerns	Most important confounders were adjusted for. Some residual confounding cannot be excluded
What is the predicted direction of bias due to confounding?	Insufficient information available	The direction of residual confounding cannot be determined.
Is the risk of bias (due to confounding) sufficiently high, in the context of its likely direction and the magnitude of the estimated exposure effect, to threaten conclusions about whether the exposure has an important effect on the outcome?	No	Residual confounding cannot be excluded, but it is unlikely to explain the observed association

Y = Yes; PY = Probably yes; PN = Probably no; N = No; SY = Strong yes; WY = Weak yes; SN = Strong no; WN = Weak no; NA = Not applicable; NI = No information

*Domain 1, variant (b): **If Y/PY to C7 and Y/PY to C8** (the analysis was based on splitting participants' follow up time according to exposure status and/or magnitude and changes in exposure status and/or magnitude likely to be related to factors that are predictive of the outcome, so both baseline and time-varying confounding need to be addressed)*

**Signalling questions**	**Response options**	**Comments**
1.1 Did the authors use an analysis method that was appropriate to control for time-varying as well as baseline confounding?	Y / PY / PN / N / NI	
1.2 **If Y/PY to 1.1**: Did the authors control for all the important baseline and time-varying confounding factors for which this was necessary?	NA / Y / PY / WN (no, but uncontrolled confounding was probably not substantial) / SN (no, and uncontrolled confounding was probably substantial) / NI	
1.3 **If Y/PY/WN to 1.2**: Were confounding factors that were controlled for (and for which control was necessary) measured validly and reliably by the variables available in this study?	NA / Y / WN (no, but the extent of measurement error in confounding factors was probably not substantial) / SN (no, and the extent of measurement error in confounding factors was probably substantial) / NI	
1.4 **If N/PN/NI to 1.1**: Did the authors control for time-varying factors or other variables measured after the start of the exposure window being studied?	NA / Y / PY / PN / N / NI	
1.5 Did the use of negative controls, or other considerations, suggest uncontrolled confounding?	Y / PY / PN / N	
Risk of bias (due to confounding) in the estimated effect of exposure on the outcome	Low risk / Some concerns / High risk / Very high risk	

Signalling questions	Response options	Comments
What is the predicted direction of bias due to confounding?	Towards benefit of (higher) exposure / Towards harm of (higher) exposure / Towards null / Away from null / Insufficient information available	
Is the risk of bias (due to confounding) sufficiently high, in the context of its likely direction and the magnitude of the estimated exposure effect, to threaten conclusions about whether the exposure has an important effect on the outcome?	Yes / No / Cannot tell	

Y = Yes; PY = Probably yes; PN = Probably no; N = No; SY = Strong yes; WY = Weak yes; SN = Strong no; WN = Weak no; NA = Not applicable; NI = No information

Domain 2: Risk of bias arising from measurement of the exposure

*Domain 2, Variant (a): **If N/PN to C5** (exposure was measured at a single point in time)*

Signalling questions	Response options	Comments
Mismeasurement or misclassification of the exposure.		
2.1 Does the measured exposure well-characterize the exposure metric specified to be of interest in this study? [*This was specified in the answers to D2, D3 and D4*]	Y / PY / WN (no, to a small extent) / SN (no, to a large extent) / NI	
2.2 Was the exposure likely to be measured with error, or misclassified?	SY (yes, probably a substantial amount) / WY (yes, but probably not a substantial amount) / PN / N / NI	
Bias in the estimated effect of exposure arising from mismeasurement or misclassification of the exposure		
2.3 If SY/WY to 2.2: Could mismeasurement or misclassification of exposure have been differential (i.e. related to the outcome or risk of the outcome)?	NA / SY (yes, to a large extent) / WY (yes, to a small extent) / PN / N / NI	
2.4 If SY/WY to 2.2 and N/PN/WY to 2.3: Is non-differential measurement error likely to bias the estimated effect of exposure on outcome?	NA / SY (yes, to a large extent) / WY (yes, to a small extent) / PN / N / NI	
Risk of bias (arising from measurement of exposure) in the estimated effect of exposure on the outcome	Low risk / Some concerns / High risk / Very high risk	
What is the predicted direction of bias arising from measurement of exposure?	Towards benefit of (higher) exposure / Towards harm of (higher) exposure / Towards null / Away from null / Insufficient information available	
Is the risk of bias (arising from measurement of exposure) sufficiently high, in the context of its likely direction and the magnitude of the estimated exposure effect, to	Yes / No / Cannot tell	

Signalling questions	Response options	Comments
threaten conclusions about whether the exposure has an important effect on the outcome?		

Y = Yes; PY = Probably yes; PN = Probably no; N = No; SY = Strong yes; WY = Weak yes; SN = Strong no; WN = Weak no; NA = Not applicable; NI = No information

*Domain 2, Variant (b): **If Y/PY to C5 and Y/PY to C6** (each individual's exposure level was estimated from measurements made at multiple time points)*

Signalling questions	Response options	Comments
2.1 Does the measured exposure (derived from measurements at multiple time points) well-characterize the exposure metric specified to be of interest in this study? [*This was specified in the answers to D2, D3 and D4*]	WN	Physical activity trajectories were based on repeated measurements across multiple waves.
2.2 Was there error in measurement, or misclassification, of the exposure, at each single time point?	WY	Physical activity was self-reported. Recall bias is possible.
2.3 If SY/WY to 2.2: Could mismeasurement or misclassification of exposure have been differential (i.e. related to the outcome or risk of the outcome)?	**PN**	Physical activity was assessed before frailty outcome assessment, making differential misclassification unlikely.
2.4 If SY/WY to 2.2 and N/PN/WY to 2.3: Is the nature of the (non-differential) measurement error likely to bias the estimated effect of exposure on outcome?	WY	Any remaining misclassification is likely to be non-differential and bias the results towards the null
Risk of bias (arising from measurement of exposure) in the estimated effect of exposure on the outcome	Some concerns	Repeated measurements improved exposure classification, although physical activity was self-reported.
What is the predicted direction of bias arising from measurement of exposure?	Towards null	Any remaining measurement error would likely bias the results towards the null.
Is the risk of bias (arising from measurement of exposure) sufficiently high, in the context of its likely direction and the magnitude of the estimated exposure effect, to threaten conclusions about whether the exposure has an important effect on the outcome?	No	Repeated measurements reduced exposure misclassification. The remaining measurement error is unlikely to have affected the results

Y = Yes; PY = Probably yes; SN = Strong no; WN = Weak no; NA = Not applicable; NI = No information

*Domain 2, Variant (c): **If Y/PY to C5, N/PN to C6 and Y/PY to C7** (the analysis was based on splitting participants' follow up time according to exposure status and/or magnitude):*

Signalling questions	Response options	Comments
2.1 Does the measured exposure (including changes over time) well-characterize the exposure metric specified to be of interest in this study? [*This was specified in the answers to D2, D3 and D4*]	Y / PY / WN (no, to a small extent) / SN (no, to a large extent) / NI	
2.2 Was there error in measurement, or misclassification, of the exposure, at each single time point?	SY (yes, probably a substantial amount) / WY (yes, but probably not a substantial amount) / PN / N / NI	
2.3 If SY/WY to 2.2: Could mismeasurement or misclassification of exposure have been differential (i.e. related to the outcome or risk of the outcome)?	NA / SY (yes, to a large extent) / WY (yes, to a small extent) / PN / N / NI	
2.4 If SY/WY to 2.2 and N/PN/WY to 2.3: Is the nature of the (non-differential) measurement error likely to bias the estimated effect of exposure on outcome?	NA / SY (yes, to a large extent) / WY (yes, to a small extent) / PN / N / NI	
Risk of bias (arising from measurement of exposure) in the estimated effect of exposure on the outcome	Low risk / Some concerns / High risk / Very high risk	
What is the predicted direction of bias arising from measurement of exposure?	Towards benefit of (higher) exposure / Towards harm of (higher) exposure / Towards null / Away from null / Insufficient information available	
Is the risk of bias (arising from measurement of exposure) sufficiently high, in the context of its likely direction and the magnitude of the estimated exposure effect, to threaten conclusions about whether the exposure has an important effect on the outcome?	Yes / No / Cannot tell	

Y = Yes; PY = Probably yes; SN = Strong no; WN = Weak no; NA = Not applicable; NI = No information

Domain 3: Risk of bias in selection of participants into the study (or into the analysis)

Signalling questions	Response options	Comments
3.1 Did follow-up begin at (or close to) the start of the exposure window for most participants? [*The exposure window is specified in D3*]	Y	Participants entered follow-up after physical activity was classified.
3.2 **If N/PN to 3.1**: Is the effect of exposure likely to be constant over the period of follow up analysed?	PN	Physical activity may have changed during follow-up.
3.3 Was selection of participants into the study (or into the analysis) based on participant characteristics observed after the start of the exposure window being studied? [*The exposure window is specified in D3*]	Y	Participants with prevalent frailty, missing data and loss to follow-up were excluded.
3.4 **If Y/PY to 3.3**: Were these characteristics likely to be influenced by exposure or a cause of exposure?	PN	Loss to follow-up may have been related to physical activity
3.5 **If Y/PY to 3.4**: Were these characteristics likely to be influenced by the outcome or a cause of the outcome?	PN	Participants with poorer health may have been more likely to be lost to follow-up.
3.6 **If N/PN to 3.2 or Y/PY to 3.5**: Is it likely that the analysis corrected for all of the potential selection biases identified in A and B above?	PN	No methods to address selection bias were reported.
3.7 **If N/PN to 3.2 or Y/PY to 3.5**: Did sensitivity analyses demonstrate that the likely impact of the potential selection biases identified in A or B above was minimal?	WN	No sensitivity analyses for selection bias were reported.
Risk of bias (due to selection of participants into the study) in the estimated effect of exposure on the outcome	Some concerns	Participants with prevalent frailty, missing data and loss to follow-up were excluded. No specific methods to address selection bias were reported.
What is the predicted direction of bias<br><br>due to selection of participants into the study?	Towards null	Participants lost to follow-up may have had poorer health and lower physical activity. This could have biased the results towards the null.
Is the risk of bias (due to selection of participants into the study) sufficiently high, in the context of its likely direction and the magnitude of the estimated exposure effect, to	No	Selection bias cannot be excluded, but it is unlikely to have substantially affected the results

Signalling questions	Response options	Comments
threaten conclusions about whether the exposure has an important effect on the outcome?		

Y = Yes; PY = Probably yes; PN = Probably no; N = No; SN = Strong no; WN = Weak no; NA = Not applicable; NI = No information

Domain 4: Risk of bias due to post-exposure interventions

Signalling questions	Response options	Comments
4.1 Were there post-exposure interventions that were influenced by prior exposure during the follow-up period?	PN	No post-exposure interventions influenced by prior physical activity status were reported or evaluated.
4.2 **If Y/PY to 4.1**: Is it likely that the analysis corrected for the effect of post-exposure interventions that were influenced by prior exposure?	NA	Not applicable
Risk of bias (due post-exposure interventions) in the estimated effect of exposure on the outcome	Low risk	No relevant post-exposure interventions were identified.
What is the predicted direction of bias due to confounding?	Insufficient information available	No clear direction of bias can be identified.
Is the risk of bias (due post-exposure interventions) sufficiently high, in the context of its likely direction and the magnitude of the estimated exposure effect, to threaten conclusions about whether the exposure has an important effect on the outcome?	No	Post-exposure interventions are unlikely to have affected the results.

Y = Yes; PY = Probably yes; PN = Probably no; N = No; NA = Not applicable; NI = No information

Domain 5: Risk of bias due to missing data

Signalling questions	Response options	Comments
5.1 Were complete data on exposure status available for all, or nearly all, participants?	PY	Physical activity trajectories were derived from repeated assessments. Participants with insufficient exposure data were excluded before analysis.
5.2 Were complete data on the outcome available for all, or nearly all, participants?	PN	Participants who died or were lost to follow-up before outcome assessment were exc[illegible] from the analysis.
5.3 Were complete data on confounding variables available for all, or nearly all, participants?	PN	Individuals with missing covariate information were excluded from the analytical sample.
5.4 **If N/PN/NI to 5.1, 5.2 or 5.3**: Is the result based on a complete case analysis?	Y	The final analyses were conducted on participants with complete exposure, outcome and covariate data after exclusions.
5.5 **If Y/PY/NI**: Was exclusion from the analysis because of missing data (in exposure, confounders or the outcome) likely to be related to the true value of the outcome?	WY	Loss to follow-up and death may be associated with frailty risk, making selection related to the outcome plausible.
5.6 **If N/PN to 5.5**: Were all or most predictors of missingness (in exposure, confounders or the outcome) included in the analysis model?	PN	No inverse probability weighting or modelling of missingness was reported.
5.7 **If N/PN to 5.4**: Was the analysis based on imputing missing values?	NI	No multiple imputation procedures were reported.
5.8 **If Y/PY to 5.7**: Was imputation performed appropriately?	NA	
5.9 **If N/PN to 5.7**: Was an appropriate alternative method used to correct for bias due to missing data?	NI	No alternative methods to address missing data (e.g., weighting or sensitivity a[illegible] were reported.
5.10 **If PN/N/NI to 5.1, 5.2 or 5.3**: Is there evidence that the result was not biased by missing data?	PN	No sensitivity analyses, multiple imputation or inverse probability weighting were reported.
Risk of bias (due to missing data) in the estimated effect of exposure on the outcome	Some concerns	Some participants were excluded because of missing data, loss to follow-up or death. No multiple imputation or sensitivity analyses were reported.

Signalling questions	Response options	Comments
What is the predicted direction of bias due to missing data?	Towards null	Participants lost to follow-up or who died may have been frailer and less physically active. This would likely bias the results towards the null.
Is the risk of bias (due to missing data) sufficiently high, in the context of its likely direction and the magnitude of the estimated exposure effect, to threaten conclusions about whether the exposure has an important effect on the outcome?	No	Missing data may have introduced some bias, but it is unlikely to have substantially affected the results

Y = Yes; PY = Probably yes; PN = Probably no; N = No; SY = Strong yes; WY = Weak yes; NA = Not applicable; NI = No information

Domain 6: Risk of bias arising from measurement of the outcome

Signalling questions	Response options	Comments
6.1 Could measurement or ascertainment of the outcome have differed between exposure groups or levels of exposure?	N	Frailty was assessed using the same Frailty Index for all participants
6.2 Were outcome assessors aware of study participants' exposure history?	PY	Blinding of outcome assessors was not reported.
6.3 **If Y/PY/NI to 6.2**: Could assessment of the outcome have been influenced by knowledge of participants' exposure history?	PN	Frailty was assessed using a standardized instrument. Differential assessment is unlikely
Risk of bias (arising from measurement of outcomes) in the estimated effect of exposure on the outcome	Low risk	Frailty was assessed using the same standardized method for all participants.
What is the predicted direction of bias arising from measurement of outcomes?	Insufficient information available	The direction of bias cannot be determined
Is the risk of bias (arising from measurement of outcomes) sufficiently high, in the context of its likely direction and the magnitude of the estimated exposure effect, to threaten conclusions about whether the exposure has an important effect on the outcome?	No	Outcome measurement is unlikely to have affected the results.

Y = Yes; PY = Probably yes; PN = Probably no; N = No; SY = Strong yes; WY = Weak yes; NA = Not applicable; NI = No information

Domain 7: Risk of bias in selection of the reported result

Signalling questions	Response options	Comments
7.1 Was the result reported in accordance with an available, pre-determined analysis plan?	PN	No published protocol or statistical analysis plan was available.
7.2 **If N/PN/NI to 7.1**: Is the reported effect estimate likely to be selected, based on desirability of the magnitude (or statistical significance) of the estimated effect of exposure on outcome, from multiple *exposure measurements* within the exposure domain?	N	Physical activity trajectories were predefined and all trajectory groups were reported.
7.3 Is the reported effect estimate likely to be selected, based on desirability of the magnitude (or statistical significance) of the estimated effect of exposure on outcome, from multiple *outcome measurements* within the outcome domain?	N	Frailty was assessed using a predefined Frailty Index and the primary outcome was consistently reported
7.4 Is the reported effect estimate likely to be selected, based on desirability of the magnitude (or statistical significance) of the estimated effect of exposure on outcome, from multiple *analyses* of the exposure-outcome relationship?	PN	Several subgroup analyses were reported.<br>No evidence of selective reporting was found.
7.5 Is the reported effect estimate likely to be selected, based on the basis of desirability of the results (e.g. statistical significance), from different *subgroups*?	PN	Subgroup analyses were reported systematically for predefined variables, with no evidence that only favourable subgroup results were selected.
Risk of bias (due to selection of the reported result) in the estimated effect of exposure on the outcome	Low risk	No protocol was available, but no evidence of selective reporting was identified.
What is the predicted direction of bias due to selection of the reported result?	Insufficient information available	The direction of bias cannot be determined.
Is the risk of bias (due to selection of the reported result) sufficiently high, in the context of its likely direction and the magnitude of the estimated exposure effect, to threaten conclusions about whether the exposure has an important effect on the outcome?	No	Selective reporting is unlikely to have affected the results.

Y = Yes; PY = Probably yes; PN = Probably no; N = No; NA = Not applicable; NI = No information

Overall risk of bias

	Response options	Comments
Overall risk of bias	Some concerns	The study adjusted for major confounders and used repeated assessments of physical activity. Some concerns remain because of residual confounding, self-reported physical activity, missing data and loss to follow-up.
What is the predicted direction of bias?	Towards null	Residual confounding, self-reported physical activity and loss to follow-up would most likely bias the results towards the null.
Is the overall risk of bias sufficiently high, in the context of its likely direction and the magnitude of the estimated exposure effect, to threaten conclusions about whether the exposure has an important effect on the outcome?	No	The identified sources of bias are unlikely to have substantially affected the study conclusions.

This work is licensed under a Creative Commons Attribution-NonCommercial-NoDerivatives 4.0 International License.

Haapanen et al.

For each study result: preliminary considerations

A. Specify the result being assessed for risk of bias

A1. Specify the numerical result being assessed

Association between change in regular physical activity and rate of change in frailty from late midlife to old age (fully adjusted β × Time estimates).

B. Decide whether to proceed with a risk-of-bias assessment

	Response options	Comments
B1. Did the authors make any attempt to control for confounding?	Y	Adjusted for sex, marital status, childhood and adult socioeconomic status, and changes in other lifestyle factors.
B2. **If N/PN to B1**: Is there sufficient potential for confounding that an unadjusted result should not be considered further?	NA	
B3. Was the method of measuring exposure inappropriate?	N	Physical activity was assessed using standardized questionnaires at repeated study visits.
B4. Was the method of measuring the outcome inappropriate?	N	Frailty was measured using a validated Frailty Index at repeated clinical assessment

If the answer to any of B2, B3 or B4 is 'Yes' or 'Probably yes', the result should be considered to be at very high risk of bias and no further assessment is required. Otherwise, proceed to section C.

C. Specify the analysis in the current study for which results are being assessed for risk of bias

C1. Specify the outcome to which this result relates.

Rate of change in frailty (Frailty Index) from late midlife to old age

C2. Specify the participant group on which this result was based.

2000 community-dwelling adults from the Helsinki Birth Cohort Study with repeated follow-up assessments.

C3 to C8: Describe the exposure measurement(s) used to produce this result.

C3. What is the exposure being measured and how was it measured or assessed?	Physical activity was self-reported using the Kuopio Ischemic Heart Disease Risk Factor Study questionnaire and classified according to the WHO recommendation (≥12.5 MET-h/week). Measurements were obtained at repeated follow-up visits
C4. Was exposure analysed as a quantitative (rather than a categorical) variable?	N
C5. Did repeated measurements of exposure over time (for each participant) contribute to the analysis that produced this result?	Y
C6. **If Y/PY to C5**, was a single estimate of each participant's exposure level derived from the repeated measurements of exposure over time?	Y
C7. **If N/PN to C6**, was the analysis based on splitting participants' follow up time according to exposure status and/or magnitude?	NA
C8. **If Y/PY to C7**, were changes in exposure status and/or magnitude likely to be related to factors that are predictive of the outcome?	NA
C9. **If N/PN to C7**, how were repeat measurements used?	

Y = Yes; PY = Probably yes; PN = Probably no; N = No; NA = Not applicable

C10. Specify the relationship analysed to produce this result. For example, this may be a quadratic relationship of cumulative exposure with the log odds of the outcome, or a risk ratio for the outcome comparing exposed with unexposed individuals.

Associations between changes in physical activity and the rate of frailty progression were analysed using linear mixed-effects models.

D: Specify the causal effect of exposure being estimated by this result

D1. Specify the population of interest	Describe eligible participants (to whom the causal effect applies). These may be different from the study participants on whom the result was based (specified in C2). Such differences may give rise to selection biases.	Community-dwelling adults aged 57–69 years from the Helsinki Birth Cohort Study.
Specification of the exposure metric of interest		
D2. Specify the exposure	This is the factor whose causal effect on the outcome of interest is the subject of the study result being assessed. It may be thought of as the 'true' exposure of interest. It is distinct from the method with which exposure was measured.	Long-term changes in regular physical activity.
D3. Specify the exposure window	The exposure window of interest is the exposure period for which the result being assessed estimates the effect of exposure on the outcome. Specification of the exposure window is judged by the ROBINS-E user, who should aim to define a window that is both meaningful in answering the review question and broadly in line with when the study measured exposure. Specification should include both the time of onset and period of exposure. For example, it may be lifetime exposure (from birth or from conception), during ages 50-55, the period from first employment in a particular occupation, time from birth to age 10, or during pregnancy.<br><br>The specified exposure window is used to determine whether exposure data adequately reflect exposure during the window. Exposure before the start of the exposure window is addressed during the assessment of risk of bias due to confounding	Physical activity changes assessed between baseline (2001–2004) and follow-up visits (2011–2013 and 2017–2018).
D4. Specify how exposure over time	This may, for example, be ever/never exposed, cumulative exposure, average exposure, or peak exposure during the exposure	Change in physical activity status across repeated follow-up assessments.

should be summarized	period, for each participant. Alternatively, there may be only a single exposure event, or the exposure may be time invariant (such as a genetic variant or family history).	

E. Evaluation of confounding factors

Complete a row for each important confounding factor listed in advance (subsection (i)). In addition, consider any further confounding factors that are either relevant to the setting of this particular study or which the study authors identified as potentially important (subsection (ii)).

"Important" confounding factors are those for which, in the context of this study, adjustment is expected to lead to an important change in the estimated effect of the exposure.

(i) Important confounding factors listed in advance						
Confounding factor	Measured variable(s) for this factor, if any	Was this variable (or were these variables) controlled for in the analysis?<br>(Y / N)	If this confounding factor was controlled for, was it measured validly and reliably by this variable (or these variables)?*<br>(NA / Y / PY / PN / N / NI)	If this confounding factor was not controlled for, is there evidence that controlling for it was unnecessary?**<br>(NA / Y / PY / PN / N)	Is failure to adjust for this confounding factor expected to bias the effect estimate towards benefit or harm of (higher) exposure?***<br>(Benefit of (higher) exposure / Harm of (higher) exposure / Insufficient information available)	Comments
Age	Age (years)	y		NA	Benefit of (higher) exposure	Used as the underlying time scale
Sex	Male/female	y		NA	Benefit of (higher) exposure	Recorded at baseline.
BMI	Not measured; percentage body fat was used instead	N	NA	Y	NA	Not measured. They used percentage body fat instead of BMI.
**Baseline frailty status**	Baseline Frailty Index	N	Y	PN	Benefit of (higher) exposure	Baseline FI was measured but not

	(37-item FI)					adjusted as a covariate.
**Comorbidities**	Diseases were included in the Frailty Index	N	NA	Y	Benefit of (higher) exposure	**Not adjusted individually.** Diseases were part of the Frailty Index but not included as covariates.
**Lifestyle factors**	Changes in the other lifestyle factors (diet, sleep, smoking, alcohol consumption, body fat)	Y	Y	NA	Benefit of (higher) exposure	Models adjusted for changes in the other lifestyle factors.
**Socioeconomic staus**	Childhood SES and adult SES	Y	Y	NA	Benefit of (higher) exposure	Childhood and adult SES were included in the models.

(ii) Additional confounding factors relevant to the setting of this particular study, or identified by study authors and considered to be important, or which were identified since the protocol was written						
Confounding factor	Measured variable(s) for this factor, if any	Was this variable (or were these variables) controlled for in the analysis? (Y / N)	If this confounding factor was controlled for, was it measured validly and reliably by this variable (or these variables)?* (NA / Y / PY / PN / N / NI)	If this confounding factor was not controlled for, is there evidence that controlling for it was unnecessary?** (NA / Y / PY / PN / N)	Is failure to adjust for this confounding factor expected to bias the effect estimate towards benefit or harm of (higher) exposure?*** (Benefit of (higher) exposure / Harm of (higher) exposure / Insufficient information available)	Comments

Marital status	Marital status (married, not married, divorced, widowed)	Y	Y	NA	Benefit of (higher) exposure	Included in the fully adjusted models.
						Included in the fully adjusted models.

* “Validity” refers to whether the confounding variable or variables accurately measure the confounding factor, while “reliability” refers to the precision of the measurement (more measurement error means less reliability).

** In the context of a particular study, variables need not be included in the analysis: (a) if they are measured validly and reliably and are not associated with the outcome, conditional on exposure (noting that lack of a statistically significant association is not evidence of a lack of association); (b) if they are measured validly and reliably and are not associated with exposure; (c) if they are measured validly and reliably and adjustment makes no or minimal difference to the estimated effect of the primary parameter; (d) because the confounder was addressed in the study design, for example by restricting to individuals with the same value of the confounder; (e) because a negative control demonstrates that there was unlikely to have been confounding due to this variable or that uncontrolled confounding was likely to be minimal; or (f) because external evidence suggests that controlling for the variable is not necessary in the context of the study being assessed..

For each study: risk of bias assessment

Domain 1: Risk of bias due to confounding

Domain 1, Variant (a): ***If N/PN to C5 or Y/PY to C6 or N/PN to C7*** *(only baseline confounding needs to be addressed)*

Signalling questions	Response options	Comments
1.1 Did the authors control for all the important confounding factors for which this was necessary?	WN	The analyses were adjusted for age, sex, marital status, childhood and adult socioeconomic status, and changes in the other lifestyle factors. However, baseline frailty and comorbidities were not adjusted separately.
1.2 **If Y/PY/WN to 1.1**: Were confounding factors that were controlled for (and for which control was necessary) measured validly and reliably by the variables available in this study?	Y	Confounders were clearly defined and measured using standardized methods
1.3 **If Y/PY/WN to 1.1**: Did the authors control for any variables after the start of the exposure period being studied that could have been affected by the exposure?	Y	Models adjusted for changes in other lifestyle factors during follow-up.
1.4 Did the use of negative controls, or other considerations, suggest serious uncontrolled confounding?	N	No evidence of serious uncontrolled confounding.
Risk of bias (due to confounding) in the estimated effect of exposure on the outcome	Some concerns	Most important confounders were adjusted for, but baseline frailty and comorbidities were not included. Residual confounding remains possible.
What is the predicted direction of bias due to confounding?	Towards benefit of (higher) exposure	Uncontrolled confounding would likely overestimate the protective effect of healthy lifestyle.
Is the risk of bias (due to confounding) sufficiently high, in the context of its likely direction and the magnitude of the estimated exposure effect, to threaten conclusions about whether the exposure has an important effect on the outcome?	No	Residual confounding is unlikely to materially affect the main conclusions.

Y = Yes; PY = Probably yes; PN = Probably no; N = No; SY = Strong yes; WY = Weak yes; SN = Strong no; WN = Weak no; NA = Not applicable; NI = No information

*Domain 1, variant (b): **If Y/PY to C7 and Y/PY to C8** (the analysis was based on splitting participants' follow up time according to exposure status and/or magnitude and changes in exposure status and/or magnitude likely to be related to factors that are predictive of the outcome, so both baseline and time-varying confounding need to be addressed)*

**Signalling questions**	**Response options**	**Comments**
1.1 Did the authors use an analysis method that was appropriate to control for time-varying as well as baseline confounding?	Y / PY / PN / N / NI	
1.2 **If Y/PY to 1.1**: Did the authors control for all the important baseline and time-varying confounding factors for which this was necessary?	NA / Y / PY / WN (no, but uncontrolled confounding was probably not substantial) / SN (no, and uncontrolled confounding was probably substantial) / NI	
1.3 **If Y/PY/WN to 1.2**: Were confounding factors that were controlled for (and for which control was necessary) measured validly and reliably by the variables available in this study?	NA / Y / WN (no, but the extent of measurement error in confounding factors was probably not substantial) / SN (no, and the extent of measurement error in confounding factors was probably substantial) / NI	
1.4 **If N/PN/NI to 1.1**: Did the authors control for time-varying factors or other variables measured after the start of the exposure window being studied?	NA / Y / PY / PN / N / NI	
1.5 Did the use of negative controls, or other considerations, suggest uncontrolled confounding?	Y / PY / PN / N	
Risk of bias (due to confounding) in the estimated effect of exposure on the outcome	Low risk / Some concerns / High risk / Very high risk	

Signalling questions	Response options	Comments
What is the predicted direction of bias due to confounding?	Towards benefit of (higher) exposure / Towards harm of (higher) exposure / Towards null / Away from null / Insufficient information available	
Is the risk of bias (due to confounding) sufficiently high, in the context of its likely direction and the magnitude of the estimated exposure effect, to threaten conclusions about whether the exposure has an important effect on the outcome?	Yes / No / Cannot tell	

Y = Yes; PY = Probably yes; PN = Probably no; N = No; SY = Strong yes; WY = Weak yes; SN = Strong no; WN = Weak no; NA = Not applicable; NI = No information

Domain 2: Risk of bias arising from measurement of the exposure

Domain 2, Variant (a): ***If N/PN to C5*** *(exposure was measured at a single point in time)*

Signalling questions	Response options	Comments
Mismeasurement or misclassification of the exposure.		
2.1 Does the measured exposure well-characterize the exposure metric specified to be of interest in this study? [*This was specified in the answers to D2, D3 and D4*]	Y / PY / WN (no, to a small extent) / SN (no, to a large extent) / NI	
2.2 Was the exposure likely to be measured with error, or misclassified?	SY (yes, probably a substantial amount) / WY (yes, but probably not a substantial amount) / PN / N / NI	
Bias in the estimated effect of exposure arising from mismeasurement or misclassification of the exposure		
2.3 If SY/WY to 2.2: Could mismeasurement or misclassification of exposure have been differential (i.e. related to the outcome or risk of the outcome)?	NA / SY (yes, to a large extent) / WY (yes, to a small extent) / PN / N / NI	
2.4 If SY/WY to 2.2 and N/PN/WY to 2.3: Is non-differential measurement error likely to bias the estimated effect of exposure on outcome?	NA / SY (yes, to a large extent) / WY (yes, to a small extent) / PN / N / NI	
Risk of bias (arising from measurement of exposure) in the estimated effect of exposure on the outcome	Low risk / Some concerns / High risk / Very high risk	
What is the predicted direction of bias arising from measurement of exposure?	Towards benefit of (higher) exposure / Towards harm of (higher) exposure / Towards null / Away from null / Insufficient information available	
Is the risk of bias (arising from measurement of exposure) sufficiently high, in the context of its likely direction and the magnitude of the estimated exposure effect, to threaten conclusions about whether the exposure has an important effect on the outcome?	Yes / No / Cannot tell	

Y = Yes; PY = Probably yes; PN = Probably no; N = No; SY = Strong yes; WY = Weak yes; SN = Strong no; WN = Weak no; NA = Not applicable; NI = No information

*Domain 2, Variant (b): **If Y/PY to C5 and Y/PY to C6** (each individual's exposure level was estimated from measurements made at multiple time points)*

**Signalling questions**	**Response options**	**Comments**
2.1 Does the measured exposure (derived from measurements at multiple time points) well-characterize the exposure metric specified to be of interest in this study? [*This was specified in the answers to D2, D3 and D4*]	Y	Repeated exposure measurements during follow-up.
2.2 Was there error in measurement, or misclassification, of the exposure, at each single time point?	WY	Lifestyle factors were mainly self-reported.
2.3 If SY/WY to 2.2: Could mismeasurement or misclassification of exposure have been differential (i.e. related to the outcome or risk of the outcome)?	N	Exposure was measured before outcome assessment
2.4 If SY/WY to 2.2 and N/PN/WY to 2.3: Is the nature of the (non-differential) measurement error likely to bias the estimated effect of exposure on outcome?	WN	Non-differential misclassification may have attenuated the associations.
Risk of bias (arising from measurement of exposure) in the estimated effect of exposure on the outcome	Some concerns	Exposure was self-reported, but assessed repeatedly using standardized questionnaires
What is the predicted direction of bias arising from measurement of exposure?	Towards null	Non-differential misclassification would likely dilute the associations.
Is the risk of bias (arising from measurement of exposure) sufficiently high, in the context of its likely direction and the magnitude of the estimated exposure effect, to threaten conclusions about whether the exposure has an important effect on the outcome?	No	Exposure misclassification is unlikely to materially affect the main conclusions.

Y = Yes; PY = Probably yes; SN = Strong no; WN = Weak no; NA = Not applicable; NI = No information

*Domain 2, Variant (c): **If Y/PY to C5, N/PN to C6 and Y/PY to C7** (the analysis was based on splitting participants' follow up time according to exposure status and/or magnitude):*

**Signalling questions**	**Response options**	**Comments**
2.1 Does the measured exposure (including changes over time) well-characterize the exposure metric specified to be of interest in this study? [*This was specified in the answers to D2, D3 and D4*]	Y / PY / WN (no, to a small extent) / SN (no, to a large extent) / NI	
2.2 Was there error in measurement, or misclassification, of the exposure, at each single time point?	SY (yes, probably a substantial amount) / WY (yes, but probably not a substantial amount) / PN / N / NI	
2.3 If SY/WY to 2.2: Could mismeasurement or misclassification of exposure have been differential (i.e. related to the outcome or risk of the outcome)?	NA / SY (yes, to a large extent) / WY (yes, to a small extent) / PN / N / NI	
2.4 If SY/WY to 2.2 and N/PN/WY to 2.3: Is the nature of the (non-differential) measurement error likely to bias the estimated effect of exposure on outcome?	NA / SY (yes, to a large extent) / WY (yes, to a small extent) / PN / N / NI	
Risk of bias (arising from measurement of exposure) in the estimated effect of exposure on the outcome	Low risk / Some concerns / High risk / Very high risk	
What is the predicted direction of bias arising from measurement of exposure?	Towards benefit of (higher) exposure / Towards harm of (higher) exposure / Towards null / Away from null / Insufficient information available	
Is the risk of bias (arising from measurement of exposure) sufficiently high, in the context of its likely direction and the magnitude of the estimated exposure effect, to threaten conclusions about whether the exposure has an important effect on the outcome?	Yes / No / Cannot tell	

Y = Yes; PY = Probably yes; SN = Strong no; WN = Weak no; NA = Not applicable; NI = No information

Domain 3: Risk of bias in selection of participants into the study (or into the analysis)

Signalling questions	Response options	Comments
3.1 Did follow-up begin at (or close to) the start of the exposure window for most participants? [*The exposure window is specified in D3*]	Y	Follow-up began close to exposure assessment.
3.2 **If N/PN to 3.1**: Is the effect of exposure likely to be constant over the period of follow up analysed?	NA	
3.3 Was selection of participants into the study (or into the analysis) based on participant characteristics observed after the start of the exposure window being studied? [*The exposure window is specified in D3*]	N	Selection was not based on post-exposure characteristics.
3.4 **If Y/PY to 3.3**: Were these characteristics likely to be influenced by exposure or a cause of exposure?	NA	
3.5 **If Y/PY to 3.4**: Were these characteristics likely to be influenced by the outcome or a cause of the outcome?	NA	
3.6 **If N/PN to 3.2 or Y/PY to 3.5**: Is it likely that the analysis corrected for all of the potential selection biases identified in A and B above?	NA	
3.7 **If N/PN to 3.2 or Y/PY to 3.5**: Did sensitivity analyses demonstrate that the likely impact of the potential selection biases identified in A or B above was minimal?	NA	
Risk of bias (due to selection of participants into the study) in the estimated effect of exposure on the outcome	Low risk	Participants were selected before outcome assessment using a cohort design.
What is the predicted direction of bias<br><br>due to selection of participants into the study?	Towards benefit of (higher) exposure	Attrition may have favoured healthier participants.
Is the risk of bias (due to selection of participants into the study) sufficiently high, in the context of its likely direction and the magnitude of the estimated exposure effect, to	No	Selection bias is unlikely to materially affect the main conclusions.

Signalling questions	Response options	Comments
threaten conclusions about whether the exposure has an important effect on the outcome?		

Y = Yes; PY = Probably yes; PN = Probably no; N = No; SN = Strong no; WN = Weak no; NA = Not applicable; NI = No information

Domain 4: Risk of bias due to post-exposure interventions

Signalling questions	Response options	Comments
4.1 Were there post-exposure interventions that were influenced by prior exposure during the follow-up period?	N	No post-exposure interventions were reported.
4.2 **If Y/PY to 4.1**: Is it likely that the analysis corrected for the effect of post-exposure interventions that were influenced by prior exposure?	NA	
Risk of bias (due post-exposure interventions) in the estimated effect of exposure on the outcome	Low risk	No post-exposure interventions likely to affect the results.
What is the predicted direction of bias due to confounding?	Insufficient information available	No clear direction of bias.
Is the risk of bias (due post-exposure interventions) sufficiently high, in the context of its likely direction and the magnitude of the estimated exposure effect, to threaten conclusions about whether the exposure has an important effect on the outcome?	No	No evidence of bias due to post-exposure interventions.

Y = Yes; PY = Probably yes; PN = Probably no; N = No; NA = Not applicable; NI = No information

Domain 5: Risk of bias due to missing data

Signalling questions	Response options	Comments
5.1 Were complete data on exposure status available for all, or nearly all, participants?	PN	Exposure data were incomplete because of loss to follow-up.
5.2 Were complete data on the outcome available for all, or nearly all, participants?	PN	Outcome data were incomplete at follow-up.
5.3 Were complete data on confounding variables available for all, or nearly all, participants?	PN	Some confounder data were missing.
5.4 **If N/PN/NI to 5.1, 5.2 or 5.3**: Is the result based on a complete case analysis?	N	Analysis was not based on a complete-case sample.
5.5 **If Y/PY/NI**: Was exclusion from the analysis because of missing data (in exposure, confounders or the outcome) likely to be related to the true value of the outcome?	WY	Missingness was likely related to participants' characteristics.
5.6 **If N/PN to 5.5**: Were all or most predictors of missingness (in exposure, confounders or the outcome) included in the analysis model?	PN	Not all predictors of missingness were included.
5.7 **If N/PN to 5.4**: Was the analysis based on imputing missing values?	N	No imputation was reported.
5.8 **If Y/PY to 5.7**: Was imputation performed appropriately?	NA	
5.9 **If N/PN to 5.7**: Was an appropriate alternative method used to correct for bias due to missing data?	NA	
5.10 **If PN/N/NI to 5.1, 5.2 or 5.3**: Is there evidence that the result was not biased by missing data?	PN	
Risk of bias (due to missing data) in the estimated effect of exposure on the outcome	Some concerns	Loss to follow-up may have introduced bias.

Signalling questions	Response options	Comments
What is the predicted direction of bias due to missing data?	Towards benefit of (higher) exposure	Healthier participants were more likely to remain in the study.
Is the risk of bias (due to missing data) sufficiently high, in the context of its likely direction and the magnitude of the estimated exposure effect, to threaten conclusions about whether the exposure has an important effect on the outcome?	No	Missing data are unlikely to materially affect the main conclusions.

Y = Yes; PY = Probably yes; PN = Probably no; N = No; SY = Strong yes; WY = Weak yes; NA = Not applicable; NI = No information

Domain 6: Risk of bias arising from measurement of the outcome

Signalling questions	Response options	Comments
6.1 Could measurement or ascertainment of the outcome have differed between exposure groups or levels of exposure?	PN	Same Frailty Index used for all participants.
6.2 Were outcome assessors aware of study participants' exposure history?	PN	Outcome assessment was standardized.
6.3 **If Y/PY/NI to 6.2**: Could assessment of the outcome have been influenced by knowledge of participants' exposure history?	NA	
Risk of bias (arising from measurement of outcomes) in the estimated effect of exposure on the outcome	Low risk	Standardized FI assessment minimized measurement bias.
What is the predicted direction of bias arising from measurement of outcomes?	Insufficient information available	No clear direction of bias.
Is the risk of bias (arising from measurement of outcomes) sufficiently high, in the context of its likely direction and the magnitude of the estimated exposure effect, to threaten conclusions about whether the exposure has an important effect on the outcome?	No	Outcome measurement is unlikely to affect the conclusions

Y = Yes; PY = Probably yes; PN = Probably no; N = No; SY = Strong yes; WY = Weak yes; NA = Not applicable; NI = No information

Domain 7: Risk of bias in selection of the reported result

Signalling questions	Response options	Comments
7.1 Was the result reported in accordance with an available, pre-determined analysis plan?	NI	No protocol/analysis plan reported.
7.2 **If N/PN/NI to 7.1**: Is the reported effect estimate likely to be selected, based on desirability of the magnitude (or statistical significance) of the estimated effect of exposure on outcome, from multiple *exposure measurements* within the exposure domain?	PN	Exposure assessment was predefined.
7.3 Is the reported effect estimate likely to be selected, based on desirability of the magnitude (or statistical significance) of the estimated effect of exposure on outcome, from multiple *outcome measurements* within the outcome domain?	PN	Frailty Index predefined.
7.4 Is the reported effect estimate likely to be selected, based on desirability of the magnitude (or statistical significance) of the estimated effect of exposure on outcome, from multiple *analyses* of the exposure-outcome relationship?	PN	No evidence of selective analysis.
7.5 Is the reported effect estimate likely to be selected, based on the basis of desirability of the results (e.g. statistical significance), from different *subgroups*?	PN	No evidence of selective subgroup reporting.
Risk of bias (due to selection of the reported result) in the estimated effect of exposure on the outcome	Low risk	Reported results appear consistent with the study objectives and analyses.
What is the predicted direction of bias due to selection of the reported result?	*Insufficient information available*	No clear direction of bias.
Is the risk of bias (due to selection of the reported result) sufficiently high, in the context of its likely direction and the magnitude of the estimated exposure effect, to	No	No evidence of selective reporting that would meaningfully affect the estimated association or study conclusions.

Signalling questions	Response options	Comments
threaten conclusions about whether the exposure has an important effect on the outcome?		

Y = Yes; PY = Probably yes; PN = Probably no; N = No; NA = Not applicable; NI = No information

Overall risk of bias

	Response options	Comments
Overall risk of bias	Low risk of bias except for concerns about uncontrolled confounding	Most bias domains were low risk. Residual confounding cannot be fully excluded.
What is the predicted direction of bias?	Insufficient information available	No clear overall direction of bias.
Is the overall risk of bias sufficiently high, in the context of its likely direction and the magnitude of the estimated exposure effect, to threaten conclusions about whether the exposure has an important effect on the outcome?	No	Remaining limitations are unlikely to materially affect the estimated association or the study conclusions.

This work is licensed under a Creative Commons Attribution-NonCommercial-NoDerivatives 4.0 International License.

Hayashi et al.

For each study result: preliminary considerations

A. Specify the result being assessed for risk of bias

A1. Specify the numerical result being assessed

Longitudinal association between duration of participation in community-based resistance exercise and progression of frailty measured by repeated KCL scores (linear mixed-effects model; β coefficients and estimated marginal means).

B. Decide whether to proceed with a risk-of-bias assessment

	Response options	Comments
B1. Did the authors make any attempt to control for confounding?	Y	Adjusted analyses included age, sex, systolic blood pressure, pulse rate, and change in baseline KCL score.
B2. **If N/PN to B1**: Is there sufficient potential for confounding that an unadjusted result should not be considered further?	Y	Confounding is likely because participation in community exercise was not randomized and participant characteristics could influence both exercise duration and frailty progression.
B3. Was the method of measuring exposure inappropriate?	N	Exercise exposure was objectively classified according to repeated participation in standardized physical fitness tests conducted throughout follow-up.
B4. Was the method of measuring the outcome inappropriate?	N	Frailty was repeatedly assessed using the validated Kihon Checklist (KCL), a standardized instrument for frailty assessment

If the answer to any of B2, B3 or B4 is 'Yes' or 'Probably yes', the result should be considered to be at very high risk of bias and no further assessment is required. Otherwise, proceed to section C.

C. Specify the analysis in the current study for which results are being assessed for risk of bias

C1. Specify the outcome to which this result relates.

Longitudinal change in frailty measured by the continuous Kihon Checklist (KCL) score.

C2. Specify the participant group on which this result was based.

2567 community-dwelling older adults participating in the "Lively 100-year-old physical exercise" program

C3 to C8: Describe the exposure measurement(s) used to produce this result.

C3. What is the exposure being measured and how was it measured or assessed?	Duration of participation in a community-based resistance exercise program, measured objectively from repeated physical fitness test records and classified into short-, mid-, and long-term participation groups according to the number of completed assessments.
C4. Was exposure analysed as a quantitative (rather than a categorical) variable?	N
C5. Did repeated measurements of exposure over time (for each participant) contribute to the analysis that produced this result?	Y
C6. **If Y/PY to C5**, was a single estimate of each participant's exposure level derived from the repeated measurements of exposure over time?	Y
C7. **If N/PN to C6**, was the analysis based on splitting participants' follow up time according to exposure status and/or magnitude?	NA
C8. **If Y/PY to C7**, were changes in exposure status and/or magnitude likely to be related to factors that are predictive of the outcome?	NA
C9. **If N/PN to C7**, how were repeat measurements used?	

Y = Yes; PY = Probably yes; PN = Probably no; N = No; NA = Not applicable

C10. Specify the relationship analysed to produce this result. For example, this may be a quadratic relationship of cumulative exposure with the log odds of the outcome, or a risk ratio for the outcome comparing exposed with unexposed individuals.

Association between cumulative duration of participation in community-based resistance exercise (short-, mid-, and long-term) and longitudinal change in continuous KCL frailty score estimated using linear mixed-effects models.

D: Specify the causal effect of exposure being estimated by this result

D1. Specify the population of interest	Describe eligible participants (to whom the causal effect applies). These may be different from the study participants on whom the result was based (specified in C2). Such differences may give rise to selection biases.	Community-dwelling older adults eligible to participate in community-based resistance exercise programmes.
Specification of the exposure metric of interest		
D2. Specify the exposure	This is the factor whose causal effect on the outcome of interest is the subject of the study result being assessed. It may be thought of as the 'true' exposure of interest. It is distinct from the method with which exposure was measured.	Duration of participation in community-based group resistance exercise.
D3. Specify the exposure window	The exposure window of interest is the exposure period for which the result being assessed estimates the effect of exposure on the outcome. Specification of the exposure window is judged by the ROBINS-E user, who should aim to define a window that is both meaningful in answering the review question and broadly in line with when the study measured exposure. Specification should include both the time of onset and period of exposure. For example, it may be lifetime exposure (from birth or from conception), during ages 50-55, the period from first employment in a particular occupation, time from birth to age 10, or during pregnancy.<br><br>The specified exposure window is used to determine whether exposure data adequately reflect exposure during the window. Exposure before the start of the exposure window is addressed during the assessment of risk of bias due to confounding	From initiation of participation in the exercise programme until the last completed physical fitness assessment (April 2010–December 2019).

D4. Specify how exposure over time should be summarized	This may, for example, be ever/never exposed, cumulative exposure, average exposure, or peak exposure during the exposure period, for each participant. Alternatively, there may be only a single exposure event, or the exposure may be time invariant (such as a genetic variant or family history).	Cumulative participation over time, categorized as short-term (<3 assessments), mid-term (4–6 assessments), and long-term (7–13 assessments).

E. Evaluation of confounding factors

Complete a row for each important confounding factor listed in advance (subsection (i)). In addition, consider any further confounding factors that are either relevant to the setting of this particular study or which the study authors identified as potentially important (subsection (ii)).

"Important" confounding factors are those for which, in the context of this study, adjustment is expected to lead to an important change in the estimated effect of the exposure.

(i) Important confounding factors listed in advance						
Confounding factor	Measured variable(s) for this factor, if any	Was this variable (or were these variables) controlled for in the analysis?<br>(Y / N)	If this confounding factor was controlled for, was it measured validly and reliably by this variable (or these variables)?*<br>(NA / Y / PY / PN / N / NI)	If this confounding factor was not controlled for, is there evidence that controlling for it was unnecessary?**<br>(NA / Y / PY / PN / N)	Is failure to adjust for this confounding factor expected to bias the effect estimate towards benefit or harm of (higher) exposure?***<br>(Benefit of (higher) exposure / Harm of (higher) exposure / Insufficient information available)	Comments
Age	Age(years)	Y	Y	NA	NA	Age was objectively recorded and adjusted for in all multivariable mixed-effects models.
Sex	Male/female	Y	Y	NA	NA	Sex was recorded at baseline and included as a covariate.
BMI	Body mass index	N	N		Towards benefit of higher exposure	BMI was not collected. Residual confounding by body composition cannot be excluded

Baseline health/comorbidities	Chronic diseases	N	NA	PN	Towards benefit of higher exposure	History of diseases and comorbidities were unavailable because secondary data were used, as acknowledged by the authors.
Baseline frailty / functional status	Baseline KCL score	Y	Y	NA	NA	Baseline KCL score (change from baseline) was incorporated into the adjusted model, accounting for initial frailty status.
Lifestyle factors	Smoking/alcohol intake	N	NA	PN	Towards benefit of higher exposure	Lifestyle factors (e.g. smoking, alcohol, diet, habitual physical activity outside the programme) were not assessed.
Socioeconomic status	Not measured	N	NA	PN	Towards benefit of higher exposure	Socioeconomic characteristics were not collected and may have influenced sustained participation.

(ii) Additional confounding factors relevant to the setting of this particular study, or identified by study authors and considered to be important, or which were identified since the protocol was written						
Confounding factor	Measured variable(s) for this factor, if any	Was this variable (or were these variables) controlled for in the analysis?<br>(Y / N)	If this confounding factor was controlled for, was it measured validly and reliably by this variable (or these variables)?*<br>(NA / Y / PY / PN / N / NI)	If this confounding factor was not controlled for, is there evidence that controlling for it was unnecessary?**<br>(NA / Y / PY / PN / N)	Is failure to adjust for this confounding factor expected to bias the effect estimate towards benefit or harm of (higher) exposure?*** (Benefit of (higher) exposure / Harm of (higher) exposure / Insufficient information available)	Comments

* “Validity” refers to whether the confounding variable or variables accurately measure the confounding factor, while “reliability” refers to the precision of the measurement (more measurement error means less reliability).

** In the context of a particular study, variables need not be included in the analysis: (a) if they are measured validly and reliably and are not associated with the outcome, conditional on exposure (noting that lack of a statistically significant association is not evidence of a lack of association); (b) if they are measured validly and reliably and are not associated with exposure; (c) if they are measured validly and reliably and adjustment makes no or minimal difference to the estimated effect of the primary parameter; (d) because the confounder was addressed in the study design, for example by restricting to individuals with the same value of the confounder; (e) because a negative control demonstrates that there was unlikely to have been confounding due to this variable or that uncontrolled confounding was likely to be minimal; or (f) because external evidence suggests that controlling for the variable is not necessary in the context of the study being assessed..

For each study: risk of bias assessment

Domain 1: Risk of bias due to confounding

Domain 1, Variant (a): ***If N/PN to C5 or Y/PY to C6 or N/PN to C7*** *(only baseline confounding needs to be addressed)*

Signalling questions	Response options	Comments
1.1 Did the authors control for all the important confounding factors for which this was necessary?	WN	The analyses adjusted for age, sex, systolic blood pressure, pulse rate and baseline KCL score. However, important confounders such as comorbidities and lifestyle factors were unavailable.
1.2 **If Y/PY/WN to 1.1**: Were confounding factors that were controlled for (and for which control was necessary) measured validly and reliably by the variables available in this study?	PY	Measured confounders were obtained from standardized assessments and routine records. However, some important confounders were unavailable
1.3 **If Y/PY/WN to 1.1**: Did the authors control for any variables after the start of the exposure period being studied that could have been affected by the exposure?	N	No post-exposure variables were adjusted for in the primary analyses.
1.4 Did the use of negative controls, or other considerations, suggest serious uncontrolled confounding?	PN	No negative control analyses were reported, but there was no direct evidence of serious uncontrolled confounding.
Risk of bias (due to confounding) in the estimated effect of exposure on the outcome	Some concerns	Participants with longer exercise participation may have been healthier and more health-conscious at baseline, potentially overestimating the protective effect of long-term exercise.
What is the predicted direction of bias due to confounding?	(Towards benefit of (higher) exposure	Participants with longer exercise participation may have been healthier and more health-conscious at baseline, potentially overestimating the protective effect of long-term exercise
Is the risk of bias (due to confounding) sufficiently high, in the context of its likely direction and the magnitude of the estimated exposure effect, to threaten conclusions about whether the exposure has an important effect on the outcome?	No	Although some residual confounding may remain, adjustment for major baseline confounders makes it unlikely that the observed association is entirely explained by bias.

Y = Yes; PY = Probably yes; PN = Probably no; N = No; SY = Strong yes; WY = Weak yes; SN = Strong no; WN = Weak no; NA = Not applicable; NI = No information

*Domain 1, variant (b): **If Y/PY to C7 and Y/PY to C8** (the analysis was based on splitting participants' follow up time according to exposure status and/or magnitude and changes in exposure status and/or magnitude likely to be related to factors that are predictive of the outcome, so both baseline and time-varying confounding need to be addressed)*

**Signalling questions**	**Response options**	**Comments**
1.1 Did the authors use an analysis method that was appropriate to control for time-varying as well as baseline confounding?	Y / PY / PN / N / NI	
1.2 **If Y/PY to 1.1**: Did the authors control for all the important baseline and time-varying confounding factors for which this was necessary?	NA / Y / PY / WN (no, but uncontrolled confounding was probably not substantial) / SN (no, and uncontrolled confounding was probably substantial) / NI	
1.3 **If Y/PY/WN to 1.2**: Were confounding factors that were controlled for (and for which control was necessary) measured validly and reliably by the variables available in this study?	NA / Y / WN (no, but the extent of measurement error in confounding factors was probably not substantial) / SN (no, and the extent of measurement error in confounding factors was probably substantial) / NI	
1.4 **If N/PN/NI to 1.1**: Did the authors control for time-varying factors or other variables measured after the start of the exposure window being studied?	NA / Y / PY / PN / N / NI	
1.5 Did the use of negative controls, or other considerations, suggest uncontrolled confounding?	Y / PY / PN / N	
Risk of bias (due to confounding) in the estimated effect of exposure on the outcome	Low risk / Some concerns / High risk / Very high risk	

Signalling questions	Response options	Comments
What is the predicted direction of bias due to confounding?	Towards benefit of (higher) exposure / Towards harm of (higher) exposure / Towards null / Away from null / Insufficient information available	
Is the risk of bias (due to confounding) sufficiently high, in the context of its likely direction and the magnitude of the estimated exposure effect, to threaten conclusions about whether the exposure has an important effect on the outcome?	Yes / No / Cannot tell	

Y = Yes; PY = Probably yes; PN = Probably no; N = No; SY = Strong yes; WY = Weak yes; SN = Strong no; WN = Weak no; NA = Not applicable; NI = No information

Domain 2: Risk of bias arising from measurement of the exposure

*Domain 2, Variant (a): **If N/PN to C5** (exposure was measured at a single point in time)*

Signalling questions	Response options	Comments
Mismeasurement or misclassification of the exposure.		
2.1 Does the measured exposure well-characterize the exposure metric specified to be of interest in this study? [*This was specified in the answers to D2, D3 and D4*]	Y / PY / WN (no, to a small extent) / SN (no, to a large extent) / NI	
2.2 Was the exposure likely to be measured with error, or misclassified?	SY (yes, probably a substantial amount) / WY (yes, but probably not a substantial amount) / PN / N / NI	
Bias in the estimated effect of exposure arising from mismeasurement or misclassification of the exposure		
2.3 If SY/WY to 2.2: Could mismeasurement or misclassification of exposure have been differential (i.e. related to the outcome or risk of the outcome)?	NA / SY (yes, to a large extent) / WY (yes, to a small extent) / PN / N / NI	
2.4 If SY/WY to 2.2 and N/PN/WY to 2.3: Is non-differential measurement error likely to bias the estimated effect of exposure on outcome?	NA / SY (yes, to a large extent) / WY (yes, to a small extent) / PN / N / NI	
Risk of bias (arising from measurement of exposure) in the estimated effect of exposure on the outcome	Low risk / Some concerns / High risk / Very high risk	
What is the predicted direction of bias arising from measurement of exposure?	Towards benefit of (higher) exposure / Towards harm of (higher) exposure / Towards null / Away from null / Insufficient information available	
Is the risk of bias (arising from measurement of exposure) sufficiently high, in the context of its likely direction and the magnitude of the estimated exposure effect, to	Yes / No / Cannot tell	

Signalling questions	Response options	Comments
threaten conclusions about whether the exposure has an important effect on the outcome?		

Y = Yes; PY = Probably yes; PN = Probably no; N = No; SY = Strong yes; WY = Weak yes; SN = Strong no; WN = Weak no; NA = Not applicable; NI = No information

*Domain 2, Variant (b): **If Y/PY to C5 and Y/PY to C6** (each individual's exposure level was estimated from measurements made at multiple time points)*

Signalling questions	Response options	Comments
2.1 Does the measured exposure (derived from measurements at multiple time points) well-characterize the exposure metric specified to be of interest in this study? [*This was specified in the answers to D2, D3 and D4*]	Y	Exercise participation was derived from repeated objectively recorded physical test sessions over the study period and used to classify participants into short-, mid-, and long-term participation groups.
2.2 Was there error in measurement, or misclassification, of the exposure, at each single time point?	WN	Participation was recorded objectively through programme attendance/physical test records, making substantial misclassification unlikely
2.3 If SY/WY to 2.2: Could mismeasurement or misclassification of exposure have been differential (i.e. related to the outcome or risk of the outcome)?	N	Exposure was recorded before outcome assessment and was unlikely to depend on participants' frailty progression.
2.4 If SY/WY to 2.2 and N/PN/WY to 2.3: Is the nature of the (non-differential) measurement error likely to bias the estimated effect of exposure on outcome?	PN	Any remaining misclassification is likely to be minimal and unlikely to materially affect the estimated association
Risk of bias (arising from measurement of exposure) in the estimated effect of exposure on the outcome	Low risk	Exposure was derived from repeated objectively recorded participation in the exercise programme over multiple time points. This approach is likely to have accurately captured long-term exposure, resulting in a low risk of exposure measurement bias.
What is the predicted direction of bias arising from measurement of exposure?	Towards null	Exposure was assessed objectively from repeated programme records. Any remaining non-differential misclassification would most likely attenuate the observed association.
Is the risk of bias (arising from measurement of exposure) sufficiently high, in the context of its likely direction and the magnitude of the estimated exposure effect, to threaten conclusions about whether the exposure has an important effect on the outcome?	No	The objective and repeated assessment of exposure makes substantial measurement bias unlikely; therefore, the study conclusions are unlikely to be materially affected.

Y = Yes; PY = Probably yes; SN = Strong no; WN = Weak no; NA = Not applicable; NI = No information

Domain 2, Variant (c): ***If Y/PY to C5, N/PN to C6 and Y/PY to C7*** *(the analysis was based on splitting participants' follow up time according to exposure status and/or magnitude):*

Signalling questions	Response options	Comments
2.1 Does the measured exposure (including changes over time) well-characterize the exposure metric specified to be of interest in this study? [*This was specified in the answers to D2, D3 and D4*]	Y / PY / WN (no, to a small extent) / SN (no, to a large extent) / NI	
2.2 Was there error in measurement, or misclassification, of the exposure, at each single time point?	SY (yes, probably a substantial amount) / WY (yes, but probably not a substantial amount) / PN / N / NI	
2.3 If SY/WY to 2.2: Could mismeasurement or misclassification of exposure have been differential (i.e. related to the outcome or risk of the outcome)?	NA / SY (yes, to a large extent) / WY (yes, to a small extent) / PN / N / NI	
2.4 If SY/WY to 2.2 and N/PN/WY to 2.3: Is the nature of the (non-differential) measurement error likely to bias the estimated effect of exposure on outcome?	NA / SY (yes, to a large extent) / WY (yes, to a small extent) / PN / N / NI	
Risk of bias (arising from measurement of exposure) in the estimated effect of exposure on the outcome	Low risk / Some concerns / High risk / Very high risk	
What is the predicted direction of bias<br><br>arising from measurement of exposure?	Towards benefit of (higher) exposure / Towards harm of (higher) exposure / Towards null / Away from null / Insufficient information available	
Is the risk of bias (arising from measurement of exposure) sufficiently high, in the context of its likely direction and the magnitude of the estimated exposure effect, to threaten conclusions about whether the exposure has an important effect on the outcome?	Yes / No / Cannot tell	

Y = Yes; PY = Probably yes; SN = Strong no; WN = Weak no; NA = Not applicable; NI = No information

Domain 3: Risk of bias in selection of participants into the study (or into the analysis)

Signalling questions	Response options	Comments
3.1 Did follow-up begin at (or close to) the start of the exposure window for most participants? [*The exposure window is specified in D3*]	Y	Participants entered follow-up when they started participating in the community exercise programme.
3.2 **If N/PN to 3.1**: Is the effect of exposure likely to be constant over the period of follow up analysed?	Y	The effect of sustained exercise participation was assumed to accumulate over time and was modelled longitudinally.
3.3 Was selection of participants into the study (or into the analysis) based on participant characteristics observed after the start of the exposure window being studied? [*The exposure window is specified in D3*]	Y	Participants were classified according to the total number of completed exercise sessions, and only individuals with available repeated assessments were included in the analysis.
3.4 **If Y/PY to 3.3**: Were these characteristics likely to be influenced by exposure or a cause of exposure?	Y	Continued participation and the number of completed assessments were directly influenced by exposure duration.
3.5 **If Y/PY to 3.4**: Were these characteristics likely to be influenced by the outcome or a cause of the outcome?	PY	Participants experiencing worsening frailty may have been less likely to continue attending the programme or complete follow-up assessments.
3.6 **If N/PN to 3.2 or Y/PY to 3.5**: Is it likely that the analysis corrected for all of the potential selection biases identified in A and B above?	PY	Selection bias related to continued participation may not be fully accounted for.
3.7 **If N/PN to 3.2 or Y/PY to 3.5**: Did sensitivity analyses demonstrate that the likely impact of the potential selection biases identified in A or B above was minimal?	WN	Matched sensitivity analyses produced similar results, although they did not fully address potential selection bias due to attrition.
Risk of bias (due to selection of participants into the study) in the estimated effect of exposure on the outcome	Some concerns	Selection into the analysis depended on continued participation and availability of repeated assessments. Although sensitivity analyses yielded similar results, selection bias related to attrition cannot be excluded.
What is the predicted direction of bias due to selection of participants into the study?	Towards benefit of (higher) exposure	Participants who remained in the programme and completed repeated assessments were likely healthier and less frail, potentially leading to an overestimation of the protective effect of long-term participation.

Signalling questions	Response options	Comments
Is the risk of bias (due to selection of participants into the study) sufficiently high, in the context of its likely direction and the magnitude of the estimated exposure effect, to threaten conclusions about whether the exposure has an important effect on the outcome?	No	Potential selection bias due to attrition is unlikely to be large enough to threaten the study conclusions, as the main findings remained consistent in sensitivity analyses.

Y = Yes; PY = Probably yes; PN = Probably no; N = No; SN = Strong no; WN = Weak no; NA = Not applicable; NI = No information

Domain 4: Risk of bias due to post-exposure interventions

Signalling questions	Response options	Comments
4.1 Were there post-exposure interventions that were influenced by prior exposure during the follow-up period?	n	No post-exposure interventions influenced by exposure status were reported during follow-up
4.2 **If Y/PY to 4.1**: Is it likely that the analysis corrected for the effect of post-exposure interventions that were influenced by prior exposure?	NA	Not applicable.
Risk of bias (due post-exposure interventions) in the estimated effect of exposure on the outcome	Low risk	No evidence of post-exposure interventions that could have biased the estimated association.
What is the predicted direction of bias due to confounding?	Insufficient information available	No relevant post-exposure interventions were identified.
Is the risk of bias (due post-exposure interventions) sufficiently high, in the context of its likely direction and the magnitude of the estimated exposure effect, to threaten conclusions about whether the exposure has an important effect on the outcome?	No	No post-exposure interventions likely to influence the observed association were identified.

Y = Yes; PY = Probably yes; PN = Probably no; N = No; NA = Not applicable; NI = No information

Domain 5: Risk of bias due to missing data

Signalling questions	Response options	Comments
5.1 Were complete data on exposure status available for all, or nearly all, participants?	PY	No imputation of missing data was reported.
5.2 Were complete data on the outcome available for all, or nearly all, participants?	PY	Outcome data were available for nearly all participants included in the analysis.
5.3 Were complete data on confounding variables available for all, or nearly all, participants?	PY	Most confounder data were available for analysed participants.
5.4 **If N/PN/NI to 5.1, 5.2 or 5.3**: Is the result based on a complete case analysis?	NA	
5.5 **If Y/PY/NI**: Was exclusion from the analysis because of missing data (in exposure, confounders or the outcome) likely to be related to the true value of the outcome?	NA	
5.6 **If N/PN to 5.5**: Were all or most predictors of missingness (in exposure, confounders or the outcome) included in the analysis model?	NA	
5.7 **If N/PN to 5.4**: Was the analysis based on imputing missing values?	N	No imputation of missing data was reported.
5.8 **If Y/PY to 5.7**: Was imputation performed appropriately?	NA	
5.9 **If N/PN to 5.7**: Was an appropriate alternative method used to correct for bias due to missing data?	N	*No alternative statistical method for correcting missing data bias was reported*
5.10 **If PN/N/NI to 5.1, 5.2 or 5.3**: Is there evidence that the result was not biased by missing data?	N	No evidence was provided demonstrating that missing data could not have biased the results.
Risk of bias (due to missing data) in the estimated effect of exposure on the outcome	Some concerns	The analysis was based on participants with available repeated assessments. Missing data handling methods (e.g., multiple imputation) were not reported, and evidence that missing data did not bias the results was limited.

Signalling questions	Response options	Comments
What is the predicted direction of bias due to missing data?	Insufficient information available	The potential impact and direction of bias due to missing data cannot be determined from the reported information.
Is the risk of bias (due to missing data) sufficiently high, in the context of its likely direction and the magnitude of the estimated exposure effect, to threaten conclusions about whether the exposure has an important effect on the outcome?	No	Although missing data handling was limited, there is no clear evidence that missing data were sufficient to threaten the study conclusions

Y = Yes; PY = Probably yes; PN = Probably no; N = No; SY = Strong yes; WY = Weak yes; NA = Not applicable; NI = No information

Domain 6: Risk of bias arising from measurement of the outcome

Signalling questions	Response options	Comments
6.1 Could measurement or ascertainment of the outcome have differed between exposure groups or levels of exposure?	N	The outcome was assessed using the same standardized instrument and procedures across all exposure groups.
6.2 Were outcome assessors aware of study participants' exposure history?	NI	Blinding of outcome assessors was not reported
6.3 **If Y/PY/NI to 6.2**: Could assessment of the outcome have been influenced by knowledge of participants' exposure history?	NI	There is insufficient information to determine whether knowledge of exposure influenced outcome assessment.
Risk of bias (arising from measurement of outcomes) in the estimated effect of exposure on the outcome	Low risk	Frailty was measured using a standardized and validated instrument applied uniformly across participants. There is no evidence that outcome measurement differed according to exposure status
What is the predicted direction of bias arising from measurement of outcomes?	Insufficient information available	No evidence suggests systematic differential outcome measurement, and the direction of any potential bias cannot be determined.
Is the risk of bias (arising from measurement of outcomes) sufficiently high, in the context of its likely direction and the magnitude of the estimated exposure effect, to threaten conclusions about whether the exposure has an important effect on the outcome?	No	Any potential bias arising from outcome measurement is unlikely to threaten the study conclusions.

Y = Yes; PY = Probably yes; PN = Probably no; N = No; SY = Strong yes; WY = Weak yes; NA = Not applicable; NI = No information

Domain 7: Risk of bias in selection of the reported result

Signalling questions	Response options	Comments
7.1 Was the result reported in accordance with an available, pre-determined analysis plan?	PN	No pre-specified protocol or statistical analysis plan was available to verify that the reported analyses were pre-defined.
7.2 **If N/PN/NI to 7.1**: Is the reported effect estimate likely to be selected, based on desirability of the magnitude (or statistical significance) of the estimated effect of exposure on outcome, from multiple *exposure measurements* within the exposure domain?	NI	Insufficient information to determine whether the reported result was selected from multiple exposure measurements.
7.3 Is the reported effect estimate likely to be selected, based on desirability of the magnitude (or statistical significance) of the estimated effect of exposure on outcome, from multiple *outcome measurements* within the outcome domain?	NI	Insufficient information to determine whether the reported result was selected from multiple exposure measurements.
7.4 Is the reported effect estimate likely to be selected, based on desirability of the magnitude (or statistical significance) of the estimated effect of exposure on outcome, from multiple *analyses* of the exposure-outcome relationship?	NI	Insufficient information to determine whether the reported result was selected from multiple analyses of the exposure–outcome relationship.
7.5 Is the reported effect estimate likely to be selected, based on the basis of desirability of the results (e.g. statistical significance), from different *subgroups*?	PN	No evidence of selective reporting based on subgroup analyses was identified.
Risk of bias (due to selection of the reported result) in the estimated effect of exposure on the outcome	Low risk	Although no pre-specified analysis plan was available, no evidence of selective reporting was identified. Therefore, the risk of bias due to selection of the reported result was judged to be low.
What is the predicted direction of bias due to selection of the reported result?	Insufficient information available	The direction of any potential bias due to selective reporting cannot be determined.

Signalling questions	Response options	Comments
Is the risk of bias (due to selection of the reported result) sufficiently high, in the context of its likely direction and the magnitude of the estimated exposure effect, to threaten conclusions about whether the exposure has an important effect on the outcome?	No	Although a pre-specified analysis plan was unavailable, there is no clear evidence that selective reporting was sufficient to threaten the study conclusions.

Y = Yes; PY = Probably yes; PN = Probably no; N = No; NA = Not applicable; NI = No information

Overall risk of bias

	Response options	Comments
Overall risk of bias	Some concerns	The study was generally well conducted, with standardized exposure and outcome assessment and adjustment for major baseline confounders. However, some concerns remain due to potential residual confounding, limited information on missing data handling, and the absence of a pre-specified analysis plan.
What is the predicted direction of bias?	Towards benefit of (higher) exposure	Residual confounding and participant selection may have favoured healthier individuals with longer participation, potentially overestimating the protective effect of sustained physical activity.
Is the overall risk of bias sufficiently high, in the context of its likely direction and the magnitude of the estimated exposure effect, to threaten conclusions about whether the exposure has an important effect on the outcome?	No	Some concerns remained due to the possibility of residual confounding, potential selection bias related to participant retention over repeated assessments, limited information regarding the impact of missing data, and the absence of a pre-specified analysis plan to rule out selective reporting. However, these limitations were not considered sufficient to threaten the overall study conclusions.

This work is licensed under a Creative Commons Attribution-NonCommercial-NoDerivatives 4.0 International License.

Zhang et al.

For each study result: preliminary considerations

A. Specify the result being assessed for risk of bias

A1. Specify the numerical result being assessed

B. Decide whether to proceed with a risk-of-bias assessment

	Response options	Comments
B1. Did the authors make any attempt to control for confounding?	Y	Adjusted for age, sex, smoking, drinking history, years of schooling, BMI, comorbidity, living arrangement and dietary protein intake diversity.
B2. **If N/PN to B1**: Is there sufficient potential for confounding that an unadjusted result should not be considered further?	NA	NA
B3. Was the method of measuring exposure inappropriate?	PN	Physical activity was self-reported using two simple questions without information on frequency, duration or intensity. Although exposure measurement was crude and susceptible to misclassification, it was applied consistently to all participants and was not inappropriate enough to preclude risk-of-bias assessment.
B4. Was the method of measuring the outcome inappropriate?	N	Incident frailty was assessed using a standardized Frailty Index with baseline frailty excluded before follow-up.

If the answer to any of B2, B3 or B4 is 'Yes' or 'Probably yes', the result should be considered to be at very high risk of bias and no further assessment is required. Otherwise, proceed to section C.

C. Specify the analysis in the current study for which results are being assessed for risk of bias

C1. Specify the outcome to which this result relates.

Incident frailty

C2. Specify the participant group on which this result was based.

7006 community-dwelling Chinese adults aged ≥60 years who were non-frail at baseline and participated in the Chinese Longitudinal Healthy Longevity Survey (CLHLS).

C3 to C8: Describe the exposure measurement(s) used to produce this result.

C3. What is the exposure being measured and how was it measured or assessed?	Persistent aerobic exercise, assessed by self-report using two interviewer-administered questions ("Do you exercise regularly at present?" and "Did you exercise regularly in the past?"). Participants answering "yes" to both questions were classified as engaging in persistent aerobic exercise.
C4. Was exposure analysed as a quantitative (rather than a categorical) variable?	N
C5. Did repeated measurements of exposure over time (for each participant) contribute to the analysis that produced this result?	N
C6. **If Y/PY to C5**, was a single estimate of each participant's exposure level derived from the repeated measurements of exposure over time?	NA
C7. **If N/PN to C6**, was the analysis based on splitting participants' follow up time according to exposure status and/or magnitude?	NA
C8. **If Y/PY to C7**, were changes in exposure status and/or magnitude likely to be related to factors that are predictive of the outcome?	NA
C9. **If N/PN to C7**, how were repeat measurements used?	

Y = Yes; PY = Probably yes; PN = Probably no; N = No; NA = Not applicable

C10. Specify the relationship analysed to produce this result. For example, this may be a quadratic relationship of cumulative exposure with the log odds of the outcome, or a risk ratio for the outcome comparing exposed with unexposed individuals.

Hazard ratio for incident frailty comparing participants with persistent aerobic exercise to those without persistent aerobic exercise, estimated using Cox proportional hazards regression.

D: Specify the causal effect of exposure being estimated by this result

D1. Specify the population of interest	Describe eligible participants (to whom the causal effect applies). These may be different from the study participants on whom the result was based (specified in C2). Such differences may give rise to selection biases.	7006 community-dwelling Chinese adults aged ≥60 years who were non-frail at baseline
Specification of the exposure metric of interest		
D2. Specify the exposure	This is the factor whose causal effect on the outcome of interest is the subject of the study result being assessed. It may be thought of as the 'true' exposure of interest. It is distinct from the method with which exposure was measured.	Persistent aerobic exercise.
D3. Specify the exposure window	The exposure window of interest is the exposure period for which the result being assessed estimates the effect of exposure on the outcome. Specification of the exposure window is judged by the ROBINS-E user, who should aim to define a window that is both meaningful in answering the review question and broadly in line with when the study measured exposure. Specification should include both the time of onset and period of exposure. For example, it may be lifetime exposure (from birth or from conception), during ages 50-55, the period from first employment in a particular occupation, time from birth to age 10, or during pregnancy.<br><br>The specified exposure window is used to determine whether exposure data adequately reflect exposure during the window. Exposure before the start of the exposure window is addressed during the assessment of risk of bias due to confounding	Past and current regular aerobic exercise before baseline, classified according to participants' self-reported history of exercising regularly in both the past and at present
D4. Specify how exposure over time	This may, for example, be ever/never exposed, cumulative exposure, average exposure, or peak exposure during the exposure	Persistent versus non-persistent aerobic exercise (categorical exposure).

should be summarized	period, for each participant. Alternatively, there may be only a single exposure event, or the exposure may be time invariant (such as a genetic variant or family history).	

E. Evaluation of confounding factors

Complete a row for each important confounding factor listed in advance (subsection (i)). In addition, consider any further confounding factors that are either relevant to the setting of this particular study or which the study authors identified as potentially important (subsection (ii)).

"Important" confounding factors are those for which, in the context of this study, adjustment is expected to lead to an important change in the estimated effect of the exposure.

(i) Important confounding factors listed in advance						
Confounding factor	Measured variable(s) for this factor, if any	Was this variable (or were these variables) controlled for in the analysis?<br><br>(Y / N)	If this confounding factor was controlled for, was it measured validly and reliably by this variable (or these variables)?*<br><br>(NA / Y / PY / PN / N / NI)	If this confounding factor was not controlled for, is there evidence that controlling for it was unnecessary?**<br><br>(NA / Y / PY / PN / N)	Is failure to adjust for this confounding factor expected to bias the effect estimate towards benefit or harm of (higher) exposure?***<br><br>(Benefit of (higher) exposure / Harm of (higher) exposure / Insufficient information available)	Comments
Age	Age (years)	Y	PY	NA	Benefit of higher exposure	Age was adjusted for in all multivariable Cox regression models.
Sex	Male/female	Y	Y	NA	Benefit of higher exposure	Sex was included as an important demographic confounder.
BMI	Body mass index	Y	Y	NA	Benefit of higher exposure	BMI was measured at baseline and included in adjusted analyses.

Baseline frailty/functional status	FI	N	Y	PY	Insufficient information	Participants with frailty at baseline were excluded, so only non-frail individuals entered the cohort. Baseline frailty was not included as an adjustment variable.
Chronic diseases/comorbiidities	Comorbidity status/index	Y	Y	NA	Benefit of higher exposure	Comorbidities were adjusted for in the multivariable model
Socioeconomic status	Education years	Y	Y	NA	Benefit of higher exposure	Years of schooling were included in the adjusted model.
Lifestyle factors	Smoking & drinking	Y	PY	NA	Benefit of higher exposure	Smoking and history was adjusted for in the multivariable models.

(ii) Additional confounding factors relevant to the setting of this particular study, or identified by study authors and considered to be important, or which were identified since the protocol was written						
Confounding factor	Measured variable(s) for this factor, if any	Was this variable (or were these variables) controlled for in the analysis? (Y / N)	If this confounding factor was controlled for, was it measured validly and reliably by this variable (or these variables)?* (NA / Y / PY / PN / N / NI)	If this confounding factor was not controlled for, is there evidence that controlling for it was unnecessary?** (NA / Y / PY / PN / N)	Is failure to adjust for this confounding factor expected to bias the effect estimate towards benefit or harm of (higher) exposure?*** (Benefit of (higher) exposure / Harm of (higher) exposure / Insufficient information available)	Comments

Living arrangement	Whether alone	Y	PY	NA	Benefit of higher exposure	Living alone was Adjusted.<br>.
High protein intake	Dietary protein intake diversity	Y	PY	NA	Benefit of higher exposure	High protein intake diversity was included as a lifestyle confounder

* "Validity" refers to whether the confounding variable or variables accurately measure the confounding factor, while "reliability" refers to the precision of the measurement (more measurement error means less reliability).

** In the context of a particular study, variables need not be included in the analysis: (a) if they are measured validly and reliably and are not associated with the outcome, conditional on exposure (noting that lack of a statistically significant association is not evidence of a lack of association); (b) if they are measured validly and reliably and are not associated with exposure; (c) if they are measured validly and reliably and adjustment makes no or minimal difference to the estimated effect of the primary parameter; (d) because the confounder was addressed in the study design, for example by restricting to individuals with the same value of the confounder; (e) because a negative control demonstrates that there was unlikely to have been confounding due to this variable or that uncontrolled confounding was likely to be minimal; or (f) because external evidence suggests that controlling for the variable is not necessary in the context of the study being assessed..

For each study: risk of bias assessment

Domain 1: Risk of bias due to confounding

Domain 1, Variant (a): ***If N/PN to C5 or Y/PY to C6 or N/PN to C7*** *(only baseline confounding needs to be addressed)*

**Signalling questions**	**Response options**	**Comments**
1.1 Did the authors control for all the important confounding factors for which this was necessary?	PY	The authors adjusted for most of the major baseline confounders, including age, sex, BMI, smoking, drinking history, education, comorbidity, living arrangement and dietary protein intake.
1.2 **If Y/PY/WN to 1.1**: Were confounding factors that were controlled for (and for which control was necessary) measured validly and reliably by the variables available in this study?	Y	Most confounders were measured using standardized questionnaires or clinical assessments, making their measurement generally reliable.
1.3 **If Y/PY/WN to 1.1**: Did the authors control for any variables after the start of the exposure period being studied that could have been affected by the exposure?	N	Only baseline confounders were included in the adjusted analyses; no post-exposure variables were controlled for.
1.4 Did the use of negative controls, or other considerations, suggest serious uncontrolled confounding?	N	There was no evidence suggesting serious uncontrolled confounding.
Risk of bias (due to confounding) in the estimated effect of exposure on the outcome	Low risk	Major baseline confounders were appropriately measured and adjusted for in the multivariable analyses, with no evidence of substantial residual confounding.
What is the predicted direction of bias due to confounding?	(Towards benefit of (higher) exposure	Any residual confounding would most likely favour the physically active group, potentially overestimating the protective association between regular aerobic exercise and incident frailty.
Is the risk of bias (due to confounding) sufficiently high, in the context of its likely direction and the magnitude of the estimated exposure effect, to threaten conclusions about whether the exposure has an important effect on the outcome?	No	Although some residual confounding cannot be entirely excluded, it is unlikely to be substantial enough to affect the estimated association or threaten the study conclusions.

Y = Yes; PY = Probably yes; PN = Probably no; N = No; SY = Strong yes; WY = Weak yes; SN = Strong no; WN = Weak no; NA = Not applicable; NI = No information

*Domain 1, variant (b): **If Y/PY to C7 and Y/PY to C8** (the analysis was based on splitting participants' follow up time according to exposure status and/or magnitude and changes in exposure status and/or magnitude likely to be related to factors that are predictive of the outcome, so both baseline and time-varying confounding need to be addressed)*

**Signalling questions**	**Response options**	**Comments**
1.1 Did the authors use an analysis method that was appropriate to control for time-varying as well as baseline confounding?	Y / PY / PN / N / NI	
1.2 **If Y/PY to 1.1**: Did the authors control for all the important baseline and time-varying confounding factors for which this was necessary?	NA / Y / PY / WN (no, but uncontrolled confounding was probably not substantial) / SN (no, and uncontrolled confounding was probably substantial) / NI	
1.3 **If Y/PY/WN to 1.2**: Were confounding factors that were controlled for (and for which control was necessary) measured validly and reliably by the variables available in this study?	NA / Y / WN (no, but the extent of measurement error in confounding factors was probably not substantial) / SN (no, and the extent of measurement error in confounding factors was probably substantial) / NI	
1.4 **If N/PN/NI to 1.1**: Did the authors control for time-varying factors or other variables measured after the start of the exposure window being studied?	NA / Y / PY / PN / N / NI	
1.5 Did the use of negative controls, or other considerations, suggest uncontrolled confounding?	Y / PY / PN / N	
Risk of bias (due to confounding) in the estimated effect of exposure on the outcome	Low risk / Some concerns / High risk / Very high risk	

Signalling questions	Response options	Comments
What is the predicted direction of bias due to confounding?	Towards benefit of (higher) exposure / Towards harm of (higher) exposure / Towards null / Away from null / Insufficient information available	
Is the risk of bias (due to confounding) sufficiently high, in the context of its likely direction and the magnitude of the estimated exposure effect, to threaten conclusions about whether the exposure has an important effect on the outcome?	Yes / No / Cannot tell	

Y = Yes; PY = Probably yes; PN = Probably no; N = No; SY = Strong yes; WY = Weak yes; SN = Strong no; WN = Weak no; NA = Not applicable; NI = No information

Domain 2: Risk of bias arising from measurement of the exposure

*Domain 2, Variant (a): **If N/PN to C5** (exposure was measured at a single point in time)*

Signalling questions	Response options	Comments
Mismeasurement or misclassification of the exposure.		
2.1 Does the measured exposure well-characterize the exposure metric specified to be of interest in this study? [*This was specified in the answers to D2, D3 and D4*]	WN	Regular aerobic exercise was assessed using two self-reported questions at baseline but was measured only once and did not capture changes in physical activity over time.
2.2 Was the exposure likely to be measured with error, or misclassified?	WY	Exposure was self-reported at baseline and may be subject to recall error and non-differential misclassification
Bias in the estimated effect of exposure arising from mismeasurement or misclassification of the exposure		
2.3 If SY/WY to 2.2: Could mismeasurement or misclassification of exposure have been differential (i.e. related to the outcome or risk of the outcome)?	PN	Physical activity was assessed before incident frailty occurred, making differential misclassification unlikely.
2.4 If SY/WY to 2.2 and N/PN/WY to 2.3: Is non-differential measurement error likely to bias the estimated effect of exposure on outcome?	WY	Non-differential exposure misclassification is likely to attenuate the association, biasing the estimate towards the null.
Risk of bias (arising from measurement of exposure) in the estimated effect of exposure on the outcome	Some concerns	Physical activity was self-reported only once at baseline, which may have introduced some exposure misclassification.
What is the predicted direction of bias arising from measurement of exposure?	Towards null	Self-reported physical activity is likely to cause non-differential misclassification, which would bias the association towards the null.
Is the risk of bias (arising from measurement of exposure) sufficiently high, in the context of its likely direction and the magnitude of the estimated exposure effect, to threaten conclusions about whether the exposure has an important effect on the outcome?	No	Although physical activity was self-reported only once at baseline, any resulting misclassification is unlikely to materially affect the study conclusions.

Y = Yes; PY = Probably yes; PN = Probably no; N = No; SY = Strong yes; WY = Weak yes; SN = Strong no; WN = Weak no; NA = Not applicable; NI = No information

*Domain 2, Variant (b): **If Y/PY to C5 and Y/PY to C6** (each individual's exposure level was estimated from measurements made at multiple time points)*

Signalling questions	Response options	Comments
2.1 Does the measured exposure (derived from measurements at multiple time points) well-characterize the exposure metric specified to be of interest in this study? [*This was specified in the answers to D2, D3 and D4*]	Y / PY / WN (no, to a small extent) / SN (no, to a large extent) / NI	
2.2 Was there error in measurement, or misclassification, of the exposure, at each single time point?	SY (yes, probably a substantial amount) / WY (yes, but probably not a substantial amount) / PN / N / NI	
2.3 If SY/WY to 2.2: Could mismeasurement or misclassification of exposure have been differential (i.e. related to the outcome or risk of the outcome)?	NA / SY (yes, to a large extent) / WY (yes, to a small extent) / PN / N / NI	
2.4 If SY/WY to 2.2 and N/PN/WY to 2.3: Is the nature of the (non-differential) measurement error likely to bias the estimated effect of exposure on outcome?	NA / SY (yes, to a large extent) / WY (yes, to a small extent) / PN / N / NI	
Risk of bias (arising from measurement of exposure) in the estimated effect of exposure on the outcome	Low risk / Some concerns / High risk / Very high risk	
What is the predicted direction of bias arising from measurement of exposure?	Towards benefit of (higher) exposure / Towards harm of (higher) exposure / Towards null / Away from null / Insufficient information available	
Is the risk of bias (arising from measurement of exposure) sufficiently high, in the context of its likely direction and the magnitude of the estimated exposure effect, to threaten conclusions about whether the exposure has an important effect on the outcome?	Yes / No / Cannot tell	

Y = Yes; PY = Probably yes; SN = Strong no; WN = Weak no; NA = Not applicable; NI = No information

*Domain 2, Variant (c): **If Y/PY to C5, N/PN to C6 and Y/PY to C7** (the analysis was based on splitting participants' follow up time according to exposure status and/or magnitude):*

Signalling questions	Response options	Comments
2.1 Does the measured exposure (including changes over time) well-characterize the exposure metric specified to be of interest in this study? [*This was specified in the answers to D2, D3 and D4*]	Y / PY / WN (no, to a small extent) / SN (no, to a large extent) / NI	
2.2 Was there error in measurement, or misclassification, of the exposure, at each single time point?	SY (yes, probably a substantial amount) / WY (yes, but probably not a substantial amount) / PN / N / NI	
2.3 If SY/WY to 2.2: Could mismeasurement or misclassification of exposure have been differential (i.e. related to the outcome or risk of the outcome)?	NA / SY (yes, to a large extent) / WY (yes, to a small extent) / PN / N / NI	
2.4 If SY/WY to 2.2 and N/PN/WY to 2.3: Is the nature of the (non-differential) measurement error likely to bias the estimated effect of exposure on outcome?	NA / SY (yes, to a large extent) / WY (yes, to a small extent) / PN / N / NI	
Risk of bias (arising from measurement of exposure) in the estimated effect of exposure on the outcome	Low risk / Some concerns / High risk / Very high risk	
What is the predicted direction of bias<br><br>arising from measurement of exposure?	Towards benefit of (higher) exposure / Towards harm of (higher) exposure / Towards null / Away from null / Insufficient information available	
Is the risk of bias (arising from measurement of exposure) sufficiently high, in the context of its likely direction and the magnitude of the estimated exposure effect, to threaten conclusions about whether the exposure has an important effect on the outcome?	Yes / No / Cannot tell	

Y = Yes; PY = Probably yes; SN = Strong no; WN = Weak no; NA = Not applicable; NI = No information

Domain 3: Risk of bias in selection of participants into the study (or into the analysis)

Signalling questions	Response options	Comments
3.1 Did follow-up begin at (or close to) the start of the exposure window for most participants? [*The exposure window is specified in D3*]	Y	Follow-up started immediately after baseline assessment, when aerobic exercise status was recorded.
3.2 **If N/PN to 3.1**: Is the effect of exposure likely to be constant over the period of follow up analysed?	PY	Persistent aerobic exercise was intended to represent habitual exercise status during follow-up, although measured only at baselin
3.3 Was selection of participants into the study (or into the analysis) based on participant characteristics observed after the start of the exposure window being studied? [*The exposure window is specified in D3*]	N	Participants were selected based on baseline eligibility criteria rather than characteristics arising after the exposure period
3.4 **If Y/PY to 3.3**: Were these characteristics likely to be influenced by exposure or a cause of exposure?	NA	
3.5 **If Y/PY to 3.4**: Were these characteristics likely to be influenced by the outcome or a cause of the outcome?	NA	
3.6 **If N/PN to 3.2 or Y/PY to 3.5**: Is it likely that the analysis corrected for all of the potential selection biases identified in A and B above?	NA	
3.7 **If N/PN to 3.2 or Y/PY to 3.5**: Did sensitivity analyses demonstrate that the likely impact of the potential selection biases identified in A or B above was minimal?	NA	
Risk of bias (due to selection of participants into the study) in the estimated effect of exposure on the outcome	Low risk	Participants were included according to predefined baseline eligibility criteria. There was no evidence that selection into the analysis depended on post-exposure characteristics that could introduce selection bias.
What is the predicted direction of bias<br><br>due to selection of participants into the study?	Insufficient information available	
Is the risk of bias (due to selection of participants into the study) sufficiently high, in the context of its likely direction and the magnitude of the estimated exposure effect, to	No	Although a substantial number of participants were excluded because of loss to follow-up or death, these issues relate primarily to missing data rather than selection into the analysis.

Signalling questions	Response options	Comments
threaten conclusions about whether the exposure has an important effect on the outcome?		

Y = Yes; PY = Probably yes; PN = Probably no; N = No; SN = Strong no; WN = Weak no; NA = Not applicable; NI = No information

Domain 4: Risk of bias due to post-exposure interventions

**Signalling questions**	**Response options**	**Comments**
4.1 Were there post-exposure interventions that were influenced by prior exposure during the follow-up period?	N	No post-exposure interventions influenced by baseline exercise status were reported during follow-up.
4.2 **If Y/PY to 4.1**: Is it likely that the analysis corrected for the effect of post-exposure interventions that were influenced by prior exposure?	NA	
Risk of bias (due post-exposure interventions) in the estimated effect of exposure on the outcome	Low risk	No post-exposure interventions influenced by prior exercise status were reported; therefore, bias due to post-exposure interventions is unlikely.
What is the predicted direction of bias due to confounding?	Insufficient information available	No post-exposure interventions were identified; therefore, there is no evidence to determine a specific direction of bias
Is the risk of bias (due post-exposure interventions) sufficiently high, in the context of its likely direction and the magnitude of the estimated exposure effect, to threaten conclusions about whether the exposure has an important effect on the outcome?	No	There was no evidence that post-exposure interventions threatened the validity of the estimated association between baseline exercise and incident frailty.

Y = Yes; PY = Probably yes; PN = Probably no; N = No; NA = Not applicable; NI = No information

Domain 5: Risk of bias due to missing data

Signalling questions	Response options	Comments
5.1 Were complete data on exposure status available for all, or nearly all, participants?	Y	Baseline exercise status was available for all participants included at study entry.
5.2 Were complete data on the outcome available for all, or nearly all, participants?	PN	Outcome data were unavailable for a substantial proportion of participants because of loss to follow-up and death.
5.3 Were complete data on confounding variables available for all, or nearly all, participants?	Y	Baseline confounders were collected at study entry for participants included in the analyses.
5.4 **If N/PN/NI to 5.1, 5.2 or 5.3**: Is the result based on a complete case analysis?	Y	The analyses were based on participants with complete available data.
5.5 **If Y/PY/NI**: Was exclusion from the analysis because of missing data (in exposure, confounders or the outcome) likely to be related to the true value of the outcome?	WY	Exclusion because of missing outcome data was likely related to participants' true hea [illegible] status, particularly due to advanced age and mortality.
5.6 **If N/PN to 5.5**: Were all or most predictors of missingness (in exposure, confounders or the outcome) included in the analysis model?	PN	The analysis model did not include predictors of missingness or methods to account f [illegible] informative missing data.
5.7 **If N/PN to 5.4**: Was the analysis based on imputing missing values?	N	No imputation of missing outcome data was reported.
5.8 **If Y/PY to 5.7**: Was imputation performed appropriately?	NA	
5.9 **If N/PN to 5.7**: Was an appropriate alternative method used to correct for bias due to missing data?	NA	No alternative statistical approach to address missing data was reported.
5.10 **If PN/N/NI to 5.1, 5.2 or 5.3**: Is there evidence that the result was not biased by missing data?	NA	There was no evidence demonstrating that missing data did not bias the reported results

Signalling questions	Response options	Comments
Risk of bias (due to missing data) in the estimated effect of exposure on the outcome	High risk	A substantial proportion of participants were lost to follow-up or died during follow-up. No imputation or alternative methods to address missing outcome data were reported, making bias due to missing data likely
What is the predicted direction of bias due to missing data?	Insufficient information available	Because participants lost to follow-up or who died were likely to differ systematically in frailty risk, the direction of bias cannot be determined with confidence.
Is the risk of bias (due to missing data) sufficiently high, in the context of its likely direction and the magnitude of the estimated exposure effect, to threaten conclusions about whether the exposure has an important effect on the outcome?	Yes	The extent of missing outcome data and the absence of methods to address informative missingness could materially affect the estimated association between exercise and incident frailty.

Y = Yes; PY = Probably yes; PN = Probably no; N = No; SY = Strong yes; WY = Weak yes; NA = Not applicable; NI = No information

Domain 6: Risk of bias arising from measurement of the outcome

Signalling questions	Response options	Comments
6.1 Could measurement or ascertainment of the outcome have differed between exposure groups or levels of exposure?	N	Incident frailty was assessed using the same predefined Frailty Index and identical procedures for all participants, regardless of exercise status.
6.2 Were outcome assessors aware of study participants' exposure history?	NI	The study did not report whether outcome assessors were aware of participants' baseline exercise status.
6.3 **If Y/PY/NI to 6.2**: Could assessment of the outcome have been influenced by knowledge of participants' exposure history?	PN	Even if assessors were aware of exposure status, the use of a standardized Frailty Ind[illegible] differential outcome assessment unlikely.
Risk of bias (arising from measurement of outcomes) in the estimated effect of exposure on the outcome	Low risk	Frailty was assessed using a standardized Frailty Index applied uniformly across exposure groups, making differential outcome measurement unlikely.
What is the predicted direction of bias arising from measurement of outcomes?	Insufficient information available	There is no evidence suggesting systematic differential measurement of frailty according to baseline exercise status.
Is the risk of bias (arising from measurement of outcomes) sufficiently high, in the context of its likely direction and the magnitude of the estimated exposure effect, to threaten conclusions about whether the exposure has an important effect on the outcome?	No	Outcome assessment was standardized and unlikely to introduce bias sufficient to materially affect the estimated association.

Y = Yes; PY = Probably yes; PN = Probably no; N = No; SY = Strong yes; WY = Weak yes; NA = Not applicable; NI = No information

Domain 7: Risk of bias in selection of the reported result

Signalling questions	Response options	Comments
7.1 Was the result reported in accordance with an available, pre-determined analysis plan?	NI	No pre-specified analysis plan or study protocol was available to verify whether analy[illegible] pre-defined.
7.2 **If N/PN/NI to 7.1**: Is the reported effect estimate likely to be selected, based on desirability of the magnitude (or statistical significance) of the estimated effect of exposure on outcome, from multiple *exposure measurements* within the exposure domain?	PN	There was no evidence that the reported estimate was selected from multiple exposu[illegible] measurements based on statistical significance.
7.3 Is the reported effect estimate likely to be selected, based on desirability of the magnitude (or statistical significance) of the estimated effect of exposure on outcome, from multiple *outcome measurements* within the outcome domain?	PN	There was no indication that outcome measurements were selectively reported.
7.4 Is the reported effect estimate likely to be selected, based on desirability of the magnitude (or statistical significance) of the estimated effect of exposure on outcome, from multiple *analyses* of the exposure-outcome relationship?	PN	The reported analyses appeared consistent with the study objectives, with no eviden[illegible] selective reporting of favourable analyses.
7.5 Is the reported effect estimate likely to be selected, based on the basis of desirability of the results (e.g. statistical significance), from different *subgroups*?	PN	No evidence suggested selective reporting of subgroup analyses based on statistical significance.
Risk of bias (due to selection of the reported result) in the estimated effect of exposure on the outcome	Low risk	There was no evidence of selective reporting of exposure, outcome, analytical approach, or subgroup results. Although no pre-specified analysis plan was available, the reported analyses were consistent with the study objectives.
What is the predicted direction of bias due to selection of the reported result?	Insufficient information available	There is no evidence suggesting a predictable direction of bias arising from selective reporting.

Signalling questions	Response options	Comments
Is the risk of bias (due to selection of the reported result) sufficiently high, in the context of its likely direction and the magnitude of the estimated exposure effect, to threaten conclusions about whether the exposure has an important effect on the outcome?	No	There is no indication that selective reporting was sufficient to threaten the validity of the reported association.

Y = Yes; PY = Probably yes; PN = Probably no; N = No; NA = Not applicable; NI = No information

Overall risk of bias

	Response options	Comments
Overall risk of bias	High risk	High risk of bias due to exposure misclassification and substantial loss to follow-up. Physical activity was assessed only once at baseline using a self-reported questionnaire, which may have introduced non-differential misclassification. In addition, the considerable attrition during follow-up may have resulted in selection bias despite the authors' analytical approach.
What is the predicted direction of bias?	Towards benefit of (higher) exposure	The combination of self-reported baseline exposure assessment and selective loss to follow-up is likely to have overestimated the protective association between higher physical activity and incident frailty.
Is the overall risk of bias sufficiently high, in the context of its likely direction and the magnitude of the estimated exposure effect, to threaten conclusions about whether the exposure has an important effect on the outcome?	No	The identified biases may have affected the magnitude of the estimated association; however, they are unlikely to completely undermine the overall study conclusions.

Huang et al.

For each study result: preliminary considerations

A. Specify the result being assessed for risk of bias

A1. Specify the numerical result being assessed

Physical activity was assessed prospectively using standardized self-reported questionnaires before outcome assessment. Although self-report may introduce non-differential measurement error, the exposure assessment was appropriate for this longitudinal cohort study.

B. Decide whether to proceed with a risk-of-bias assessment

	Response options	Comments
B1. Did the authors make any attempt to control for confounding?	Y	The authors adjusted for demographic, socioeconomic, lifestyle and clinical confounders using multivariable logistic regression models.
B2. **If N/PN to B1**: Is there sufficient potential for confounding that an unadjusted result should not be considered further?	NA	
B3. Was the method of measuring exposure inappropriate?	N	Physical activity was assessed prospectively using standardized self-reported questionnaires before outcome assessment.Although self-report may introduce non-differential misclassification, the exposure assessment was appropriate for a prospective cohort study.
B4. Was the method of measuring the outcome inappropriate?	N	Frailty was assessed using a standardized frailty index (32-item FI) applied uniformly to all participants.

If the answer to any of B2, B3 or B4 is 'Yes' or 'Probably yes', the result should be considered to be at very high risk of bias and no further assessment is required. Otherwise, proceed to section C.

C. Specify the analysis in the current study for which results are being assessed for risk of bias

C1. Specify the outcome to which this result relates.

Frailty transitions (frailty worsening and frailty improvement.

C2. Specify the participant group on which this result was based.

4,943 community-dwelling adults aged ≥50 years

(median age 67, 54 .9% female), from the English Longitudinal Study of Ageing (ELSA)

C3 to C8: Describe the exposure measurement(s) used to produce this result.

C3. What is the exposure being measured and how was it measured or assessed?	Self-reported physical activity (vigorous, moderate and mild intensity), assessed using standardized questionnaires at baseline and follow-up and categorized according to weekly participation frequency.
C4. Was exposure analysed as a quantitative (rather than a categorical) variable?	N
C5. Did repeated measurements of exposure over time (for each participant) contribute to the analysis that produced this result?	Y
C6. **If Y/PY to C5**, was a single estimate of each participant's exposure level derived from the repeated measurements of exposure over time?	Y
C7. **If N/PN to C6**, was the analysis based on splitting participants' follow up time according to exposure status and/or magnitude?	NA
C8. **If Y/PY to C7**, were changes in exposure status and/or magnitude likely to be related to factors that are predictive of the outcome?	NA
C9. **If N/PN to C7**, how were repeat measurements used?	

Y = Yes; PY = Probably yes; PN = Probably no; N = No; NA = Not applicable

C10. Specify the relationship analysed to produce this result. For example, this may be a quadratic relationship of cumulative exposure with the log odds of the outcome, or a risk ratio for the outcome comparing exposed with unexposed individuals.

Association between physical activity (frequency and changes over time) and frailty transitions (frailty worsening and frailty improvement), estimated using multivariable logistic regression.

D: Specify the causal effect of exposure being estimated by this result

D1. Specify the population of interest	Describe eligible participants (to whom the causal effect applies). These may be different from the study participants on whom the result was based (specified in C2). Such differences may give rise to selection biases.	Community-dwelling adults aged ≥50 years from the ELSA with complete physical activity and frailty data
Specification of the exposure metric of interest		
D2. Specify the exposure	This is the factor whose causal effect on the outcome of interest is the subject of the study result being assessed. It may be thought of as the 'true' exposure of interest. It is distinct from the method with which exposure was measured.	Physical activity (vigorous, moderate and mild).
D3. Specify the exposure window	The exposure window of interest is the exposure period for which the result being assessed estimates the effect of exposure on the outcome. Specification of the exposure window is judged by the ROBINS-E user, who should aim to define a window that is both meaningful in answering the review question and broadly in line with when the study measured exposure. Specification should include both the time of onset and period of exposure. For example, it may be lifetime exposure (from birth or from conception), during ages 50-55, the period from first employment in a particular occupation, time from birth to age 10, or during pregnancy.<br><br>The specified exposure window is used to determine whether exposure data adequately reflect exposure during the window. Exposure before the start of the exposure window is addressed during the assessment of risk of bias due to confounding	Physical activity assessed at baseline and over a mean follow-up of 3.5 years.
D4. Specify how exposure over time	This may, for example, be ever/never exposed, cumulative exposure, average exposure, or peak exposure during the exposure	Baseline physical activity category and longitudinal change in physical activity (inactive, inactive to active, active to inactive, consistently active).

should be summarized	period, for each participant. Alternatively, there may be only a single exposure event, or the exposure may be time invariant (such as a genetic variant or family history).	

E. Evaluation of confounding factors

Complete a row for each important confounding factor listed in advance (subsection (i)). In addition, consider any further confounding factors that are either relevant to the setting of this particular study or which the study authors identified as potentially important (subsection (ii)).

"Important" confounding factors are those for which, in the context of this study, adjustment is expected to lead to an important change in the estimated effect of the exposure.

(i) Important confounding factors listed in advance						
Confounding factor	Measured variable(s) for this factor, if any	Was this variable (or were these variables) controlled for in the analysis?<br>(Y / N)	If this confounding factor was controlled for, was it measured validly and reliably by this variable (or these variables)?*<br>(NA / Y / PY / PN / N / NI)	If this confounding factor was not controlled for, is there evidence that controlling for it was unnecessary?**<br>(NA / Y / PY / PN / N)	Is failure to adjust for this confounding factor expected to bias the effect estimate towards benefit or harm of (higher) exposure?***<br>(Benefit of (higher) exposure / Harm of (higher) exposure / Insufficient information available)	Comments
Age	Years	Y	Y	NA	NA	Adjusted in all multivariable models.
Sex	Male/female	Y	Y	NA	NA	Adjusted in all multivariable models.
BMI	Body mass index		Y	NA	NA	Adjusted in all multivariable models.
Baseline health/comorbidities	Diabetes/ High blood pressure	Y	Y	NA	NA	Chronic diseases were included in adjusted models.

Baseline frailty/functional statu	Baseline frailty status (FI	Y	Y	NA	NA	Baseline frailty status was accounted for by analysing frailty worsening among non-frail participants and frailty improvement among frail participants at baseline.
Lifestyle factors	Smoking, alcohol consumption	Y	Y	NA	NA	Included in adjusted models.
Socioeconomic status	Education level	Y	Y	NA	NA	Included in adjusted models.

(ii) Additional confounding factors relevant to the setting of this particular study, or identified by study authors and considered to be important, or which were identified since the protocol was written						
Confounding factor	Measured variable(s) for this factor, if any	Was this variable (or were these variables) controlled for in the analysis?<br>(Y / N)	If this confounding factor was controlled for, was it measured validly and reliably by this variable (or these variables)?*<br>(NA / Y / PY / PN / N / NI)	If this confounding factor was not controlled for, is there evidence that controlling for it was unnecessary?**<br>(NA / Y / PY / PN / N)	Is failure to adjust for this confounding factor expected to bias the effect estimate towards benefit or harm of (higher) exposure?*** (Benefit of (higher) exposure / Harm of (higher) exposure / Insufficient information available)	Comments
Ethnicity	White/non white	Y	Y	NA	NA	Included as an additional covariate.
Marital Status	Married or partnered /unmarried, separated,	Y	Y	NA	NA	Included as an additional covariate.

	divorced, or widowed					
Coronavirus symptoms	high temperature, a new continuous cough, shortness of breath or trouble breathing, fatigue, loss of sense of smell or taste, diarrhoea, abdominal pain, and loss of appetite	Y	PY	NA	NA	Included as an additional covariate in a sensitivity analysis.

* "Validity" refers to whether the confounding variable or variables accurately measure the confounding factor, while "reliability" refers to the precision of the measurement (more measurement error means less reliability).

** In the context of a particular study, variables need not be included in the analysis: (a) if they are measured validly and reliably and are not associated with the outcome, conditional on exposure (noting that lack of a statistically significant association is not evidence of a lack of association); (b) if they are measured validly and reliably and are not associated with exposure; (c) if they are measured validly and reliably and adjustment makes no or minimal difference to the estimated effect of the primary parameter; (d) because the confounder was addressed in the study design, for example by restricting to individuals with the same value of the confounder; (e) because a negative control demonstrates that there was unlikely to have been confounding due to this variable or that uncontrolled confounding was likely to be minimal; or (f) because external evidence suggests that controlling for the variable is not necessary in the context of the study being assessed..

For each study: risk of bias assessment

Domain 1: Risk of bias due to confounding

Domain 1, Variant (a): ***If N/PN to C5 or Y/PY to C6 or N/PN to C7*** *(only baseline confounding needs to be addressed)*

Signalling questions	Response options	Comments
1.1 Did the authors control for all the important confounding factors for which this was necessary?	Y	The analyses adjusted for the major baseline demographic, lifestyle and health-related confounding factors.
1.2 **If Y/PY/WN to 1.1**: Were confounding factors that were controlled for (and for which control was necessary) measured validly and reliably by the variables available in this study?	Y	Confounding factors were measured using standardized demographic and health questionnaires and established study procedures.
1.3 **If Y/PY/WN to 1.1**: Did the authors control for any variables after the start of the exposure period being studied that could have been affected by the exposure?	N	Adjustment was restricted to baseline confounding factors; no post-exposure variables were included in the primary models.
1.4 Did the use of negative controls, or other considerations, suggest serious uncontrolled confounding?	PN	Sensitivity analyses, including additional adjustment for COVID-19 symptoms, did not materially change the results. There was no clear evidence suggesting serious uncontrolled confounding.
Risk of bias (due to confounding) in the estimated effect of exposure on the outcome	Low risk	Major baseline confounding factors were appropriately measured and adjusted for, with no evidence of substantial residual confounding.
What is the predicted direction of bias due to confounding?	Insufficient information available	No important uncontrolled baseline confounding was identified; therefore, no specific direction of residual confounding can be determined.
Is the risk of bias (due to confounding) sufficiently high, in the context of its likely direction and the magnitude of the estimated exposure effect, to threaten conclusions about whether the exposure has an important effect on the outcome?	No	The identified risk of bias due to confounding is unlikely to materially affect the study conclusions.

Y = Yes; PY = Probably yes; PN = Probably no; N = No; SY = Strong yes; WY = Weak yes; SN = Strong no; WN = Weak no; NA = Not applicable; NI = No information

*Domain 1, variant (b): **If Y/PY to C7 and Y/PY to C8** (the analysis was based on splitting participants' follow up time according to exposure status and/or magnitude and changes in exposure status and/or magnitude likely to be related to factors that are predictive of the outcome, so both baseline and time-varying confounding need to be addressed)*

Signalling questions	Response options	Comments
1.1 Did the authors use an analysis method that was appropriate to control for time-varying as well as baseline confounding?	Y / PY / PN / N / NI	
1.2 **If Y/PY to 1.1**: Did the authors control for all the important baseline and time-varying confounding factors for which this was necessary?	NA / Y / PY / WN (no, but uncontrolled confounding was probably not substantial) / SN (no, and uncontrolled confounding was probably substantial) / NI	
1.3 **If Y/PY/WN to 1.2**: Were confounding factors that were controlled for (and for which control was necessary) measured validly and reliably by the variables available in this study?	NA / Y / WN (no, but the extent of measurement error in confounding factors was probably not substantial) / SN (no, and the extent of measurement error in confounding factors was probably substantial) / NI	
1.4 **If N/PN/NI to 1.1**: Did the authors control for time-varying factors or other variables measured after the start of the exposure window being studied?	NA / Y / PY / PN / N / NI	
1.5 Did the use of negative controls, or other considerations, suggest uncontrolled confounding?	Y / PY / PN / N	
Risk of bias (due to confounding) in the estimated effect of exposure on the outcome	Low risk / Some concerns / High risk / Very high risk	

Signalling questions	Response options	Comments
What is the predicted direction of bias due to confounding?	Towards benefit of (higher) exposure / Towards harm of (higher) exposure / Towards null / Away from null / Insufficient information available	
Is the risk of bias (due to confounding) sufficiently high, in the context of its likely direction and the magnitude of the estimated exposure effect, to threaten conclusions about whether the exposure has an important effect on the outcome?	Yes / No / Cannot tell	

Y = Yes; PY = Probably yes; PN = Probably no; N = No; SY = Strong yes; WY = Weak yes; SN = Strong no; WN = Weak no; NA = Not applicable; NI = No information

Domain 2: Risk of bias arising from measurement of the exposure

Domain 2, Variant (a): ***If N/PN to C5*** *(exposure was measured at a single point in time)*

Signalling questions	Response options	Comments
Mismeasurement or misclassification of the exposure.		
2.1 Does the measured exposure well-characterize the exposure metric specified to be of interest in this study? [*This was specified in the answers to D2, D3 and D4*]	Y / PY / WN (no, to a small extent) / SN (no, to a large extent) / NI	
2.2 Was the exposure likely to be measured with error, or misclassified?	SY (yes, probably a substantial amount) / WY (yes, but probably not a substantial amount) / PN / N / NI	
Bias in the estimated effect of exposure arising from mismeasurement or misclassification of the exposure		
2.3 If SY/WY to 2.2: Could mismeasurement or misclassification of exposure have been differential (i.e. related to the outcome or risk of the outcome)?	NA / SY (yes, to a large extent) / WY (yes, to a small extent) / PN / N / NI	
2.4 If SY/WY to 2.2 and N/PN/WY to 2.3: Is non-differential measurement error likely to bias the estimated effect of exposure on outcome?	NA / SY (yes, to a large extent) / WY (yes, to a small extent) / PN / N / NI	
Risk of bias (arising from measurement of exposure) in the estimated effect of exposure on the outcome	Low risk / Some concerns / High risk / Very high risk	
What is the predicted direction of bias arising from measurement of exposure?	Towards benefit of (higher) exposure / Towards harm of (higher) exposure / Towards null / Away from null / Insufficient information available	
Is the risk of bias (arising from measurement of exposure) sufficiently high, in the context of its likely direction and the magnitude of the estimated exposure effect, to threaten conclusions about whether the exposure has an important effect on the outcome?	Yes / No / Cannot tell	

Y = Yes; PY = Probably yes; PN = Probably no; N = No; SY = Strong yes; WY = Weak yes; SN = Strong no; WN = Weak no; NA = Not applicable; NI = No information

*Domain 2, Variant (b): **If Y/PY to C5 and Y/PY to C6** (each individual's exposure level was estimated from measurements made at multiple time points)*

Signalling questions	Response options	Comments
2.1 Does the measured exposure (derived from measurements at multiple time points) well-characterize the exposure metric specified to be of interest in this study? [*This was specified in the answers to D2, D3 and D4*]	Y	Repeated measurements of physical activity were used to characterize the exposure over the study period.
2.2 Was there error in measurement, or misclassification, of the exposure, at each single time point?	WY	Physical activity was self-reported at repeated time points, which may introduce some measurement error, but this is unlikely to be substantial.
2.3 If SY/WY to 2.2: Could mismeasurement or misclassification of exposure have been differential (i.e. related to the outcome or risk of the outcome)?	WY	Because physical activity was assessed repeatedly over time, later exposure measurements may have been influenced by early functional decline or preclinical frailty, making some differential exposure misclassification possible.
2.4 If SY/WY to 2.2 and N/PN/WY to 2.3: Is the nature of the (non-differential) measurement error likely to bias the estimated effect of exposure on outcome?	WY	Any remaining measurement error is likely non-differential and would attenuate the association.
Risk of bias (arising from measurement of exposure) in the estimated effect of exposure on the outcome	Some concerns	Physical activity was assessed repeatedly using self-reported measures. Although this approach adequately characterized longitudinal physical activity patterns, reporting error may have occurred, and later exposure assessments may have been influenced by existing or developing frailty.
What is the predicted direction of bias arising from measurement of exposure?	Away from null	Existing or developing frailty may have reduced participants' physical activity, potentially exaggerating the observed protective association and biasing the estimate away from the null.
Is the risk of bias (arising from measurement of exposure) sufficiently high, in the context of its likely direction and the magnitude of the estimated exposure effect, to threaten conclusions about whether the exposure has an important effect on the outcome?	No	Although some differential exposure misclassification is possible, it is unlikely to be sufficiently large to materially threaten the study conclusions.

Y = Yes; PY = Probably yes; SN = Strong no; WN = Weak no; NA = Not applicable; NI = No information

*Domain 2, Variant (c): **If Y/PY to C5, N/PN to C6 and Y/PY to C7** (the analysis was based on splitting participants' follow up time according to exposure status and/or magnitude):*

**Signalling questions**	**Response options**	**Comments**
2.1 Does the measured exposure (including changes over time) well-characterize the exposure metric specified to be of interest in this study? [*This was specified in the answers to D2, D3 and D4*]	Y / PY / WN (no, to a small extent) / SN (no, to a large extent) / NI	
2.2 Was there error in measurement, or misclassification, of the exposure, at each single time point?	SY (yes, probably a substantial amount) / WY (yes, but probably not a substantial amount) / PN / N / NI	
2.3 If SY/WY to 2.2: Could mismeasurement or misclassification of exposure have been differential (i.e. related to the outcome or risk of the outcome)?	NA / SY (yes, to a large extent) / WY (yes, to a small extent) / PN / N / NI	
2.4 If SY/WY to 2.2 and N/PN/WY to 2.3: Is the nature of the (non-differential) measurement error likely to bias the estimated effect of exposure on outcome?	NA / SY (yes, to a large extent) / WY (yes, to a small extent) / PN / N / NI	
Risk of bias (arising from measurement of exposure) in the estimated effect of exposure on the outcome	Low risk / Some concerns / High risk / Very high risk	
What is the predicted direction of bias<br><br>arising from measurement of exposure?	Towards benefit of (higher) exposure / Towards harm of (higher) exposure / Towards null / Away from null / Insufficient information available	
Is the risk of bias (arising from measurement of exposure) sufficiently high, in the context of its likely direction and the magnitude of the estimated exposure effect, to threaten conclusions about whether the exposure has an important effect on the outcome?	Yes / No / Cannot tell	

Y = Yes; PY = Probably yes; SN = Strong no; WN = Weak no; NA = Not applicable; NI = No information

Domain 3: Risk of bias in selection of participants into the study (or into the analysis)

Signalling questions	Response options	Comments
3.1 Did follow-up begin at (or close to) the start of the exposure window for most participants? [*The exposure window is specified in D3*]	Y	Follow-up began at the start of the exposure assessment period for most participants.
3.2 **If N/PN to 3.1**: Is the effect of exposure likely to be constant over the period of follow up analysed?	NA	
3.3 Was selection of participants into the study (or into the analysis) based on participant characteristics observed after the start of the exposure window being studied? [*The exposure window is specified in D3*]	PY	Participants with missing exposure or outcome data and those lost to follow-up were excluded from the analysis
3.4 **If Y/PY to 3.3**: Were these characteristics likely to be influenced by exposure or a cause of exposure?	PY	Retention in the analytical sample may have been influenced by physical activity and related health characteristics, as less active participants may have been less likely to complete follow-up.
3.5 **If Y/PY to 3.4**: Were these characteristics likely to be influenced by the outcome or a cause of the outcome?	PY	Selection occurred before the outcome assessment.
3.6 **If N/PN to 3.2 or Y/PY to 3.5**: Is it likely that the analysis corrected for all of the potential selection biases identified in A and B above?	PN	No methods were used to address selection bias
3.7 **If N/PN to 3.2 or Y/PY to 3.5**: Did sensitivity analyses demonstrate that the likely impact of the potential selection biases identified in A or B above was minimal?	WN	Sensitivity analyses did not specifically assess selection bias
Risk of bias (due to selection of participants into the study) in the estimated effect of exposure on the outcome	High risk	Substantial attrition and restriction of the analysis to participants with complete follow-up data may have introduced important selection bias, particularly because continued participation was likely related to both physical activity and frailty.
What is the predicted direction of bias due to selection of participants into the study?	Towards null	Less active and frailer participants may have been more likely to be lost to follow-up. Their exclusion may have attenuated the observed protective association between physical activity and frailty.

Signalling questions	Response options	Comments
Is the risk of bias (due to selection of participants into the study) sufficiently high, in the context of its likely direction and the magnitude of the estimated exposure effect, to threaten conclusions about whether the exposure has an important effect on the outcome?	No	Although substantial attrition may have introduced selection bias, the direction and magnitude of its impact are uncertain and are unlikely to invalidate the overall study conclusions.

Y = Yes; PY = Probably yes; PN = Probably no; N = No; SN = Strong no; WN = Weak no; NA = Not applicable; NI = No information

Domain 4: Risk of bias due to post-exposure interventions

Signalling questions	Response options	Comments
4.1 Were there post-exposure interventions that were influenced by prior exposure during the follow-up period?	N	No relevant post-exposure interventions influenced by prior physical activity were identified during follow-up.
4.2 **If Y/PY to 4.1**: Is it likely that the analysis corrected for the effect of post-exposure interventions that were influenced by prior exposure?	NA	
Risk of bias (due post-exposure interventions) in the estimated effect of exposure on the outcome	Low risk	No relevant post-exposure interventions were identified that could have affected the association between physical activity and frailty.
What is the predicted direction of bias due to confounding?	Insufficient information available	No bias due to post-exposure interventions was identified; therefore, no direction of bias can be predicted.
Is the risk of bias (due post-exposure interventions) sufficiently high, in the context of its likely direction and the magnitude of the estimated exposure effect, to threaten conclusions about whether the exposure has an important effect on the outcome?	No	Bias due to post-exposure interventions is unlikely to affect the study conclusions.

Y = Yes; PY = Probably yes; PN = Probably no; N = No; NA = Not applicable; NI = No information

Domain 5: Risk of bias due to missing data

Signalling questions	Response options	Comments
5.1 Were complete data on exposure status available for all, or nearly all, participants?	PY	Physical activity data were available for the analytical sample; however, some participants were excluded because of missing exposure or follow-up data.
5.2 Were complete data on the outcome available for all, or nearly all, participants?	PY	Frailty was assessed prospectively; however, participants lost to follow-up did not have outcome data available.
5.3 Were complete data on confounding variables available for all, or nearly all, participants?	PY	Confounding variables were comprehensively assessed, although complete availability for all participants was not explicitly reported.
5.4 **If N/PN/NI to 5.1, 5.2 or 5.3**: Is the result based on a complete case analysis?	NA	
5.5 **If Y/PY/NI**: Was exclusion from the analysis because of missing data (in exposure, confounders or the outcome) likely to be related to the true value of the outcome?	WY	Participants excluded because of missing data or loss to follow-up may have differed in frailty risk, making outcome-related missingness plausible.
5.6 **If N/PN to 5.5**: Were all or most predictors of missingness (in exposure, confounders or the outcome) included in the analysis model?	NA	
5.7 **If N/PN to 5.4**: Was the analysis based on imputing missing values?	NA	
5.8 **If Y/PY to 5.7**: Was imputation performed appropriately?	NA	
5.9 **If N/PN to 5.7**: Was an appropriate alternative method used to correct for bias due to missing data?	NA	
5.10 **If PN/N/NI to 5.1, 5.2 or 5.3**: Is there evidence that the result was not biased by missing data?	NA	
Risk of bias (due to missing data) in the estimated effect of exposure on the outcome	Some concerns	Participants with missing data or incomplete follow-up were excluded from the analysis. Although this complete-case approach may have introduced some bias

Signalling questions	Response options	Comments
What is the predicted direction of bias due to missing data?	Towards null	Loss to follow-up is more likely to attenuate than exaggerate the observed association.
Is the risk of bias (due to missing data) sufficiently high, in the context of its likely direction and the magnitude of the estimated exposure effect, to threaten conclusions about whether the exposure has an important effect on the outcome?	No	Although some bias due to missing data cannot be excluded, it is unlikely to materially alter the overall study conclusions.

Y = Yes; PY = Probably yes; PN = Probably no; N = No; SY = Strong yes; WY = Weak yes; NA = Not applicable; NI = No information

Domain 6: Risk of bias arising from measurement of the outcome

**Signalling questions**	**Response options**	**Comments**
6.1 Could measurement or ascertainment of the outcome have differed between exposure groups or levels of exposure?	N	Frailty was assessed using the same standardized 32-item Frailty Index for all participants, regardless of physical activity level.
6.2 Were outcome assessors aware of study participants' exposure history?	PN	Outcome assessment was based on standardized questionnaires and predefined scoring procedures, making assessor awareness of participants' physical activity unlikely to influence outcome ascertainment.
6.3 **If Y/PY/NI to 6.2**: Could assessment of the outcome have been influenced by knowledge of participants' exposure history?	NA	
Risk of bias (arising from measurement of outcomes) in the estimated effect of exposure on the outcome	Low risk	Frailty was measured using a standardized and validated Frailty Index applied consistently across all participants.
What is the predicted direction of bias arising from measurement of outcomes?	Insufficient information available	There is no evidence suggesting a specific direction of bias due to outcome measurement.
Is the risk of bias (arising from measurement of outcomes) sufficiently high, in the context of its likely direction and the magnitude of the estimated exposure effect, to threaten conclusions about whether the exposure has an important effect on the outcome?	No	Bias arising from outcome measurement is unlikely to affect the study conclusions.

Y = Yes; PY = Probably yes; PN = Probably no; N = No; SY = Strong yes; WY = Weak yes; NA = Not applicable; NI = No information

Domain 7: Risk of bias in selection of the reported result

Signalling questions	Response options	Comments
7.1 Was the result reported in accordance with an available, pre-determined analysis plan?	PN	No study protocol or prespecified analysis plan was available, but the reported analyses were consistent with the study objectives.
7.2 **If N/PN/NI to 7.1**: Is the reported effect estimate likely to be selected, based on desirability of the magnitude (or statistical significance) of the estimated effect of exposure on outcome, from multiple *exposure measurements* within the exposure domain?	N	There was no evidence that results were selected based on statistical significance from multiple exposure measurements.
7.3 Is the reported effect estimate likely to be selected, based on desirability of the magnitude (or statistical significance) of the estimated effect of exposure on outcome, from multiple *outcome measurements* within the outcome domain?	N	There was no evidence that results were selected from multiple outcome measurements.
7.4 Is the reported effect estimate likely to be selected, based on desirability of the magnitude (or statistical significance) of the estimated effect of exposure on outcome, from multiple *analyses* of the exposure-outcome relationship?	N	There was no evidence of selective reporting from multiple statistical analyses.
7.5 Is the reported effect estimate likely to be selected, based on the basis of desirability of the results (e.g. statistical significance), from different *subgroups*?	N	Although subgroup analyses were presented, there was no evidence that results were selectively reported based on statistical significance.
Risk of bias (due to selection of the reported result) in the estimated effect of exposure on the outcome	Low risk	No evidence of selective reporting of the study results was identified.
What is the predicted direction of bias due to selection of the reported result?	Insufficient information available	No important bias due to selective reporting was identified.
Is the risk of bias (due to selection of the reported result) sufficiently high, in the context of its likely direction and the magnitude of the estimated exposure effect, to	No	Bias due to selective reporting is unlikely to affect the study conclusions.

Signalling questions	Response options	Comments
threaten conclusions about whether the exposure has an important effect on the outcome?		

Y = Yes; PY = Probably yes; PN = Probably no; N = No; NA = Not applicable; NI = No information

Overall risk of bias

	Response options	Comments
Overall risk of bias	Some concerns	Some concerns remain because of potential bias related to participant selection, self-reported physical activity, and missing data.
What is the predicted direction of bias?	Towards null	Loss to follow-up of older and frailer participants may have attenuated the observed protective association between physical activity and frailty.
Is the overall risk of bias sufficiently high, in the context of its likely direction and the magnitude of the estimated exposure effect, to threaten conclusions about whether the exposure has an important effect on the outcome?	No	The identified sources of bias are unlikely to be large enough to change the overall study conclusions.

This work is licensed under a Creative Commons Attribution-NonCommercial-NoDerivatives 4.0 International License.

Supplemental Table S7: GRADE assessment of the certainty of evidence delivered in the meta-analysis for the impact of persistent physical activity through midlife on risk of future frailty development

**Domain**	Judgement / Evidence Summary	Grade Change
**Limitations in studies**	6 studies; all but one had moderate risk of bias; exclusion of the high-risk study did not change RR	No downgrade
**Indirectness**	Exposure and outcome measured by similar, validated tools in all studies ; tools are self-rated	No downgrade
**Inconsistency**	High heterogeneity largely explained by length of follow-up	No downgrade
**Imprecision**	nationally representative cohorts in 4/6 studies ; Systematic review studies involving 106,771 subjects and Meta-analysis studies involving 95,195 subjects ; 80% prediction interval does not include 1 for any of the examined exposures; Individual study confidence intervals did not include 1 except in 1 study	No downgrade
**Publication Bias**	Insufficient number of studies to assess asymmetry (n = 6)	No downgrade
**Large Effect Size**	Summary RRs for the different exposures range 0.51 – 0.61, a moderate effect size very close to the 0.50 cut-off for a large effect	No upgrade
**Plausible residual confounding toward null**	Estimates derived by multivariable models adjusting for most important confounders; adequate precision in the data ; at least moderate-quality, nationally representative longitudinal aging cohorts modestly treated as low-quality evidence at baseline	Upgrade 1 level
**Dose–response relation**	Although trajectory modelling and long follow-up was used in 4/6 studies, a clear dose-response analysis was not included in this data	No upgrade
**GRADE Conclusion**	Moderate-certainty evidence; mean RR=0.56 for persistent physical activity during midlife on future incidence of frailty	—

Domain	Judgement / Evidence Summary	Grade Change
**Limitations in studies**	6 studies; all but one had moderate risk of bias; exclusion of the high-risk study did not change RR	No downgrade
**Indirectness**	Exposure and outcome measured by similar, validated tools in all studies ; tools are self-rated	No downgrade
**Inconsistency**	High heterogeneity not explained by subgroup analyses	Downgrade 1 level
**Imprecision**	nationally representative cohorts in 4/6 studies ; Systematic review studies involving 106,771 subjects and Meta-analysis studies involving 95,195 subjects ; 80% prediction interval does not include 1 for any of the examined exposures; Individual study confidence intervals did not include 1 except in 1 study	No downgrade
**Publication Bias**	Insufficient number of studies to assess asymmetry (n = 6)	No downgrade
**Large Effect Size**	Summary RRs for the different exposures range 0.51 – 0.61, a moderate effect size very close to the 0.50 cut-off for a large effect	No upgrade
**Plausible residual confounding toward null**	Estimates derived by multivariable models adjusting for most important confounders; adequate precision in the data ; at least moderate-quality, nationally representative longitudinal aging cohorts modestly treated as low-quality evidence at baseline	Upgrade 1 level
**Dose–response relation**	Although trajectory modelling and long follow-up was used in 4/6 studies, a clear dose-response analysis was not included in this data	No upgrade
**GRADE Conclusion**	Low-certainty evidence; mean RR=0.62 for inclining physical activity during midlife on future incidence of frailty	—

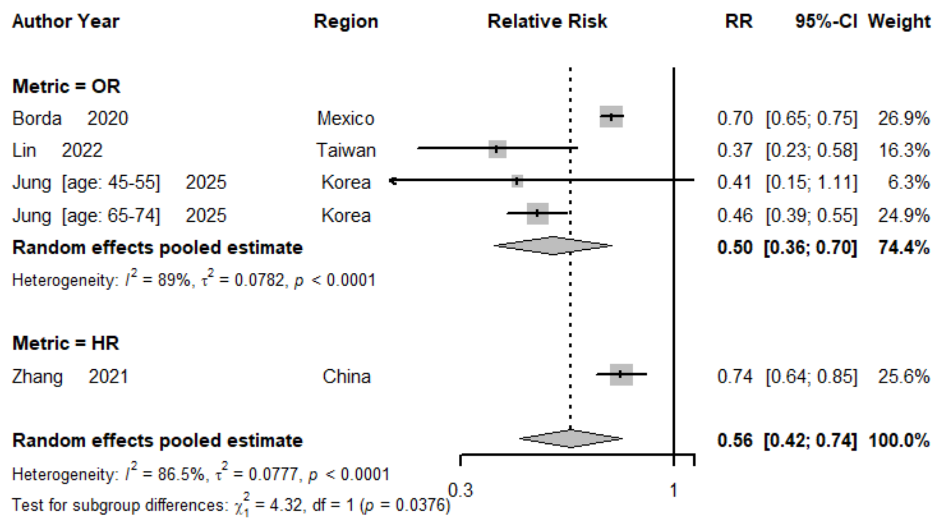

Supplemental Figure S1. persistent/consistent PA vs low BY Metric

.

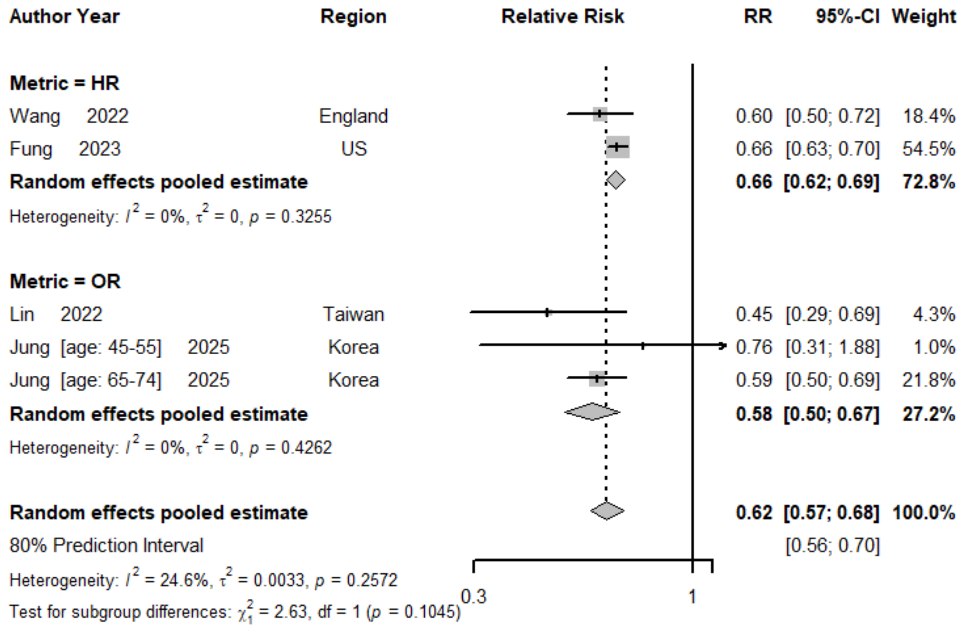

Supplemental Figure S2. inclining/increasing PA vs low by metric

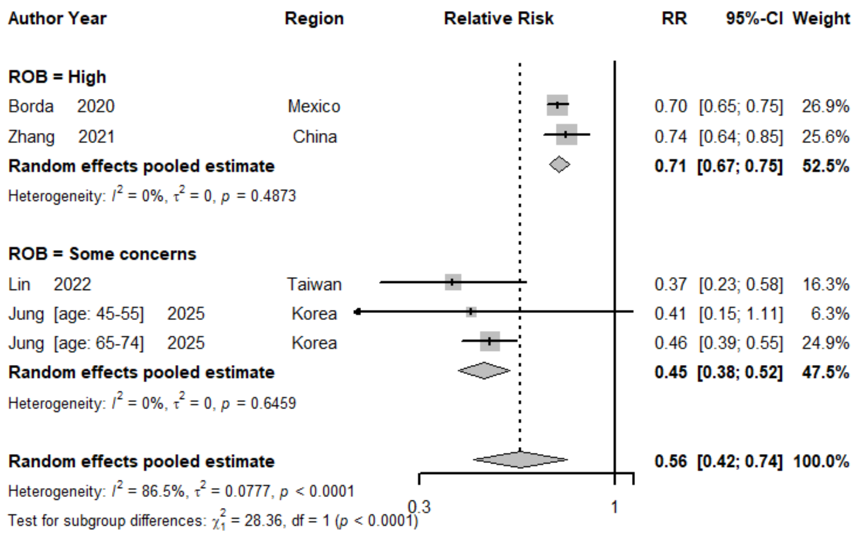

Supplemental Figure S3. persistent/consistent PA vs low BY ROB

inclining/increasing PA vs low for ROB → ALL some concerns, no subgroup
